# Picosecond infrared laser mass spectrometry for 10-second identification of lymphoproliferative imposter tumours in patient-derived xenografts

**DOI:** 10.1038/s41598-025-33064-w

**Published:** 2026-01-07

**Authors:** Lan-Anna Ye, Darah Vlaminck, Alexa Fiorante, Laurentiu G. Dabija, Francis Talbot, Julia Froment, Alhareth Azaizeh, Likun Hou, Ming Li, Yuhui Wang, Pinjiang Cao, Dani Shouk, Rani Shouk, Ming-Sound Tsao, Laurie Ailles, Catherine O’Brien, Benjamin H. Lok, Nhu-An Pham, Arash Zarrine-Afsar

**Affiliations:** 1 Princess Margaret Cancer Centre, University Health Network, 101 College Street, Room 7-207, MaRS Building, Princess Margaret Cancer Research Tower, 7th floor (STTARR), Toronto, ON M5G 1L7 Canada; 2 Department of Medical Biophysics, University of Toronto, 101 College Street, Toronto, ON M5G 1L7 Canada; 3 Present Address: Jordanian Royal Medical Services, Amman, Jordan; 4 Department of Surgery, University of Toronto, 149 College Street, Toronto, ON M5T 1P5 Canada; 5 Keenan Research Center for Biomedical Science and the Li Ka Shing Knowledge Institute, St. Michael’s Hospital, 30 Bond Street, Toronto, ON M5B 1W8 Canada

**Keywords:** Rapid diagnosis, PDX quality control, PDX failure, Lymphoproliferative tumours, Mass spectrometry, Picosecond infrared laser mass spectrometry, Molecular profiling, Patient-derived xenografts, Biomarkers, Cancer, Medical research, Oncology

## Abstract

Patient derived xenografts (PDXs) are widely used in preclinical research. However, lymphoproliferative ‘outgrowths’ at the site of tumour xenoplantation are a common source of failure in the creation of the disease model. In this work, we assessed the performance of 10-second molecular profiling of xenoplanted tissue with picosecond infrared laser mass spectrometry (PIRL-MS) as a new method for rapid identification of lymphoproliferative ‘outgrowths’ in serial passages to streamline the quality control workflow. PIRL-MS can identify ‘imposter’ lymphoproliferative tumours with sensitivity and specificity values of > 99%. This observation is established over *n* = 258 independent PDX specimens and *n* = 3,393 ten-second mass spectral data points used for building and validating (blind assessment) a classifier multivariate model to enable discrimination. We first established a classifier model based on principal component analysis coupled with linear discriminant analysis (PCA-LDA) to discriminate between true solid tumour PDXs (of 5 common epithelioid cancer types originating from lung, pancreas, ovarian, colon and head & neck as well as imposter tumours of lymphoproliferative origin. Implementation of the classifier only requires 10 seconds of mass spectral data collection (using a hand-held probe) and less than an additional second for data processing and evaluation against the model towards a classification. In addition, PIRL-MS analysis does not require any tissue preparation before analysis, and from previous research can also be deployed *in situ/in vivo* to save time. These attributes, coupled with its reported high sensitivity and specificity for identification of imposter lymphoproliferative tumours, position PIRL-MS as a rapid quality control method for fidelity assessment of xenoplanted tissues. These observations motivate follow-on work to reduce the cost and the footprint of the PIRL-MS platform towards lowering the adoption barrier for routine use.

**Supplementary Information:**

The online version contains supplementary material available at 10.1038/s41598-025-33064-w.

## Introduction

Patient-derived xenograft (PDX) tumours created in immunodeficient mice have become indispensable tools for preclinical studies of cancer^1–8^. A central tenet of their widespread use relies on the accuracy with which these xenograft models represent their parent patient tumours in both molecular and various biological characteristics. Despite their widespread use and promising utility, however, the formation of ‘lymphocytic outgrowths’ at the site of engraftment continues to be a problem that has indiscriminately plagued PDX generation across many common cancer types^9–13^. The presence of such lymphoproliferative ‘imposter tumours’ in a study is highly problematic, and their identification is costly and time-consuming. As a strategy to shield against lymphoproliferative outgrowths, for example, multiple implants are often initiated. To rationally develop a suitable screening strategy, many studies aimed to advance our mechanistic understanding of the exhibited lymphoproliferative behaviour. Building on previous demonstrations of the role of the donor Epstein-Barr virus (EBV) in the expansion of human origin B cells^14–17^, a number of screening methods have been proposed^18,19^. However, an activation of spontaneous murine origin lymphoma at the transplant site has also been seen^20^ (as a source of lymphoproliferative tissue), among others^21^. While the proposed mechanisms are diverse and debated, all the studies cited above point to a clear need for rigorous quality control (QC) and fidelity assessment in xenograft generation. Further buttressing the need for this QC assessment is the staggeringly high reports of up to 30% of some xenoplanted growths in immunodeficient mice being of lymphoproliferative nature^22^.

Currently, the fidelity assessment of a generated PDX (compared to its parent, the expected tumour type) is achieved through histological slide analysis with hematoxylin and eosin (H&E)-stained cells and sometimes by genetic sequencing. These methods are attractive because of their relatively widespread availability at preclinical centres and their low running costs (especially for H&E-based assays). However, due to their long analysis times (several hours to a few days, depending on the availability of technical or human resources), the QC evaluation step often takes place in parallel with the PDX study, or after the study interventions have commenced. As such, the lack of a decision support method for rapid assessment of PDX fidelity often results in wasted study resources, additional costs, and efforts. With PDX models projected to find novel utilities for personalized medicine in certain clinical workflows (e.g. therapeutic drug sensitivity assessment^3^ or for combinatorial treatment planning^23)^, it is imperative that a new method of PDX fidelity assessment with a short turnaround time is achieved to prevent wasted resources. Here, a promising new technology capable of characterizing tumour tissues, their types, and quality (e.g. necrotic or viable) in approximately 10-seconds (or less) utilizes an ultrafast mid-infrared laser to bring tissue molecules to the gas phase for subsequent ‘profiling’ of tissue molecular content using on-line mass spectrometry, generating a unique fingerprint for each tumour type^24^. The method, entitled picosecond infrared laser mass spectrometry (PIRL-MS), has shown promise for the 10-second differentiation of pediatric^25,26^ and adult brain cancer types^27^, including morphometrically identical yet molecularly distinct subtypes thereof^25,27,28^ as well as types and subtypes of skin cancer^29^, including actionable BRAF-V600E mutation status^30^. Here, the differentiation is achieved by comparing the mass spectral fingerprint signature of a query specimen (e.g., a xenograft tumour) as demonstrated^25,27–29,31^ to a fingerprint library of previously studied tumour or tissue types using statistical analysis methods (typically via dimensionality reduction followed by supervised multivariate approaches) in ~ 10 s of data collection and analysis time (excluding the time required to catalogue and establish the fingerprint library itself)^28^. Notably, the PIRL-MS method can perform reliable classifications from measurements performed *in vivo* and *in situ* in murine xenografts^29,32^. In this study, we examined whether PIRL-MS analysis could be used to distinguish between various commonly used PDXs of epithelioid origin from undesirable lymphocytic outgrowths with only 10 s of data collection and analysis time.

## Materials and methods

### Ethics statement

Princess Margaret Living Biobank (PMLB) is a centralized repository for PDX models banking for research uses at Princess Margaret (PM) Cancer Centre using protocols approved by the PM human research ethics committee (REB# 17-5518), following guidelines of participant informed consent, and animal care committee (AUP#5555), consistent with ARRIVE guidelines for reporting and documenting animal research^33^. In summary, all methods were performed in accordance with relevant guidelines and regulations, including human tissue data re-analyzed from a co-pending publication in submission [40].

### Generation of PDX

A total of *n* = 258 patient-derived xenograft (PDX) models of common epithelioid cancer types of lung, pancreas, ovarian, head & neck, and colon were accessed from the Princess Margaret Living Biobank (PMLB, University Health Network, Toronto, Canada). Banked < 3 mm^3^ snap frozen, or freshly harvested PDX fragments were used for PIRL-MS analysis. A serial mouse propagation of a PDX model was established from a donor banked cryopreserved or fresh PDX tissue fragment (< 3 mm^3^) implanted in a subcutaneous pocket at the flank of an immunodeficient mouse host (non-obese diabetic severe combined immune-deficient (NOD SCID) or its gamma strain (NSG)), and growth was monitored up to 6 months. Once a humane endpoint was reached, including the maximum of the largest tumour diameter of 1.5 cm with skin fold, a PDX tumour was harvested for quality control tests, downstream studies, and banking. Standard specimens collected include the largest cross-section cut for formalin-fixed and paraffin-embedded block generation for histological assessment, snap frozen fragment, viable tissue cryopreservation and fresh tumour fragments for immediate implantation into new mouse hosts for downstream PDX studies.

### PIRL-MS data collection and analysis

Sampling for PIRL-MS analysis was performed using the reported handheld probe^32^ used previously in our studies^25–27,29,30^. A 2-meter-long Tygon tube connected the inlet (150 degrees Celsius) of a Xevo G2-XS Time-of-Flight Mass Spectrometer (Waters Corp., Milford, MA, USA, operating at a duty cycle of one mass spectrum per second) to a custom-made handpiece bearing the tip of a 2-meter-long sapphire fiber (diameter 425 micrometers) delivering PIRL ablation (1 kHz, average power ~ 380–400 mW, PIRL-IV, Light Matter Interaction Inc., Etobicoke, Ontario, Canada) to the specimen (snap frozen or fresh PDX tissue fragment). Laser power was measured before and after each experiment, with a ≲ 10% drift allowed, as it has previously been shown not to influence spectral quality. The mass analyzer was calibrated per manufacturer (Waters) instructions at recommended intervals, and system health was verified by performing 10 s measurements of mouse liver and considered a pass if classified correctly, against a historic multivariate model of various mouse tissues collected over an 8-year period in the group as recommended^28^. In this study, the data was collected over a period of 46 days over a 2-year period (Jan. 2023-Dec. 2024) and processed together. Further spectral QC criteria applied per those published previously^25^ are signal duration of more than 3 seconds and total ion count (TIC) of more than 10^3^.

To address mass drift on our time-of-flight instrument, we used an internal lock-mass. Mass shifts were corrected on MassLynx (Waters Corp., Milford, MA, USA) using *m/z* 717.5070 as internal standard. The mass spectra were recorded in the negative ion mode over the range of 100-1,000 Daltons (Da) (for singly charged species) and binned to 0.1 Da, generating 9,000 independent mass-to-charge (*m/z*) values and their associated intensity (arbitrary units) and normalized to total ion count (TIC) prior to analysis. Each PDX specimen (*ex vivo* fresh and analyzed within 30 min of harvest, or previously frozen and banked at -80 degrees Celsius thawed at room temperature for 3–5 min prior to analysis) was sampled for ~ 10 s. Multiple samplings were performed across the specimen’s surface to capture intrinsic spatial heterogeneity, ensuring spatially invariant concordant predictions are achieved when the data is used. The multivariate modeling was performed on Abstract Model Builder (AMX) from Waters Research Centre (Budapest, Hungary)^34^ using principal component analysis, linear discriminant analysis (PCA-LDA), as done previously for binary PIRL-MS modeling^30^. In addition, PCA-LDA analysis in Python was conducted on the same data matrix outlined above using the following packages: SciKit-learn (model development using PCA-LDA and StratifiedGroupKFold cross-validation); imblearn (for Synthetic Minority Oversampling Technique, SMOTE). For PCA analysis the number of components that captured 95% of the variance were kept for subsequent LDA analysis. Additionally, Receiver Operating Characteristic (ROC) curve plot analysis and confusion matrices also used SciKit-learn package. Plots were generated using Matplotlib and Seaborn. Python 3.11.11 version was used for our coding.

Cross-validation of the model used a 5-fold ‘full-group’ leave-out test on AMX using standard deviation of *n* = 4 as done previously^26,27^. The classification of the unknowns in a blind manner also used AMX (recognition function) with a standard deviation of *n* = 4 and the following parameters for sampling event recognition: “number of scans per spectrum” set to 13; “wait for good spectrum timeout” of 13 s and a TIC intensity [to initiate counting the scans] of > 10^4^. The probability associated with class predictions (from AMX) is calculated from Mahalanobis distances from the cluster centre^35^. Calculations of sensitivity and specificity used standard definitions of true positive and true negative rates (utilized data points with rendered classification, excluding unclassifiable and ‘bad’ data based on which no predictions are made). For clarity, three criteria were used to define ‘bad’ data: (1) spectra in which no Lock-mass peak (*m/z* 717.5070) was found, (2) spectra with less than 3s of signal duration and, (3) spectra with less than 10^3 intensity. Similarly, unclassifiable data also affect the duty cycle of the method and are reported. Uniform manifold approximation and projection (UMAP) plots were created with R’s “UMAP” package utilizing Euclidian distance measurements, and visualized with “ggplot2”, using the number of components of 2, with the number of “epochs” set to 200, utilizing a random seed of 123, and the number of iterations of 250. All plots were visualized using *n* = 25 neighbours.

We further performed correlation coefficient analysis using mass spectral data (binned to 1 Da) as an attempt to rationalize the failures observed when classifying selected unknown specimen. This generated 900 features (from *m/z* 100 to 1000) that were subjected to a Pearson correlation coefficient analysis (in Python using Pandas’ corr() function) to generate correlation maps that can be visually compared to selected control. The control correlation maps were based on unknown specimens in which all sampling events were correctly classified (100%).

## Results

To assess the utility of PIRL-MS analysis as a novel tool for rapid assessment of engraftment fidelity during PDX generation, we subjected *n* = 50 true lymphoproliferative tissue (referred to as ‘lymphoma’for brevity) and *n* = 208 commonly utilized solid tumour PDXs of epithelioid origin to *ex vivo* 10-second PIRL-MS analysis. A subset of these specimens was used to create a classifier model for identifying imposter lymphoproliferative tumours and the rest for the validation of the model using blind tests. For each PDX, we first recorded the ‘fingerprint’ spectra over 100-1000 Da range (Fig. S1). As shown in Fig. S1, 10-second sampling with PIRL-MS generates fingerprint spectra containing several mass-to-charge (*m/z*) values unique to each tumour type. We subsequently created a multivariate model to predict lymphocytic outgrowths based on such unique fingerprints. The top 100 mass spectral features most important for this differentiation are listed in Table S1, partly further highlighted in the representative Fig. S1 spectra if visible. From our previous works^25–27,29^ and the tight coupling between lipid metabolism and cancer^36,37^, the said discrimination is likely to utilize tissue metabolites and lipids whose identities (with no bearing on the efficiency or accuracy of discrimination) will be established in a follow-up study in which we must also consider the non-trivial contributing role of murine stroma or adopt a sorted cancer cell approach.

**Fig. 1:**
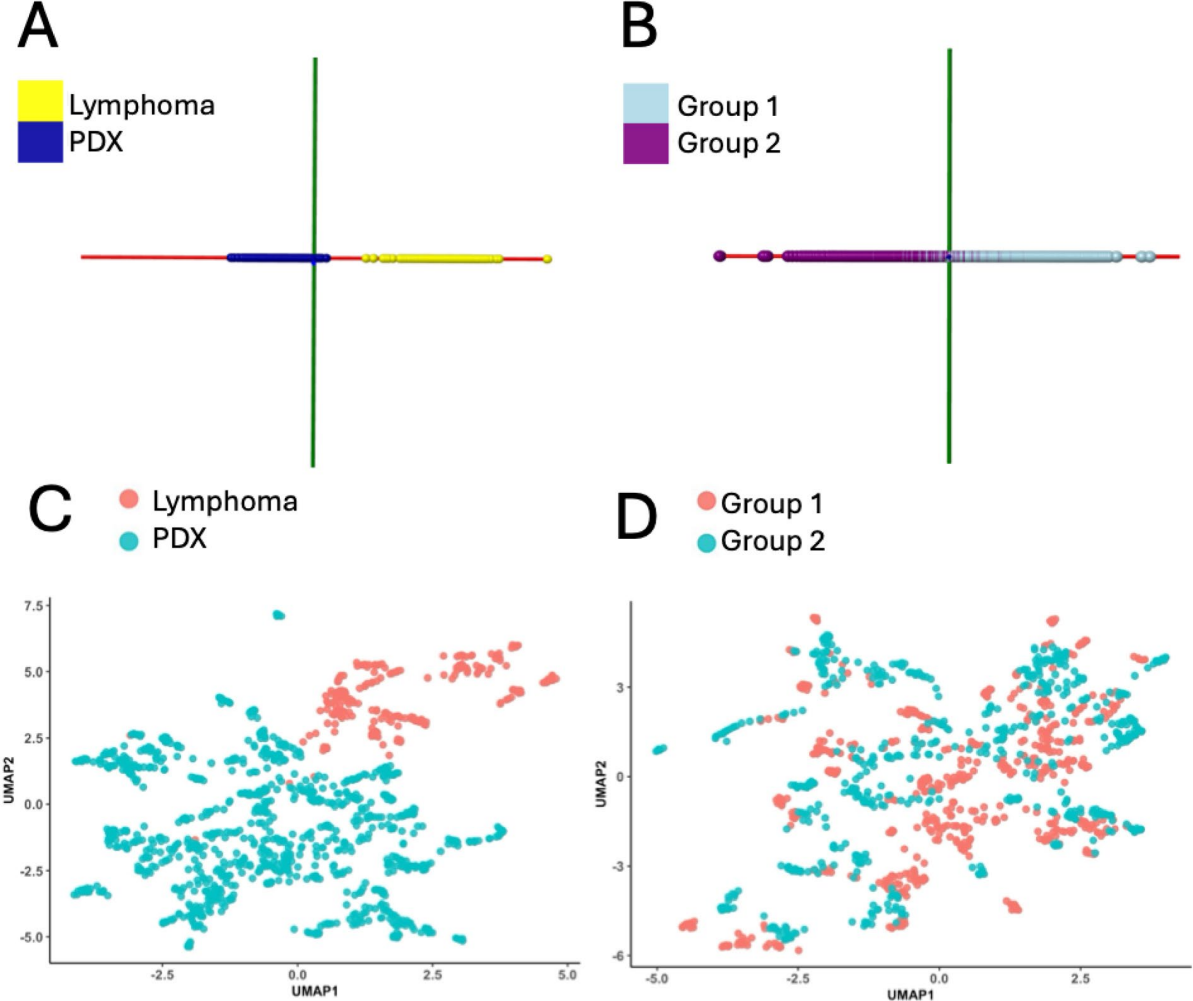
Data modeling distinguishes between lymphocytic outgrowths and true solid tumour PDXs. We show two scores plots, one for a PCA-LDA model bearing true annotation (Panel **A**) and one for a permutated version of the model in this panel bearing false annotations (Panel **B**). These PCA-LDA models (using AMX^34^ were created from *n* = 20 independent lymphocytic outgrowths (*n* = 269 data points) and *n* = 90 common solid tumour PDXs (*n* = 1,045 data points). The maximum number of PC components for these models was *n* = 262, and the permutated model (containing ~ same amount of data from both lymphocytic class and true PDX class in its 2 groups was built with *n* = 55 specimens per group 1 and 2 (*n* = 670 and *n =* 644 data points per group, respectively). Panel (**A**) model (true annotations) shows a complete separation of data from lymphocytic and control true PDX group, but the permutated model (Panel **B**) shows more mixing of data (albeit, pulled apart somewhat by the supervised LDA analysis). Consistent with Tables 1 and 2 results that describe the cross-validation accuracies of these two models using 5-fold leave-out test (100% for Panel **A** and only 48.78% for Panel **B**), 10-second PIRL-MS analysis appears to effectively distinguish between lymphocytic outgrowths and true solid tumour PDXs. The solid tumour PDX specimen numbers and sampling events (mass spectral data points, as a result of multiple samplings performed per each specimen) were as follows: colon cancer *n* = 19 (*n* = 198 data points), head & neck *n* = 18 (*n* = 227 data points), lung *n* = 16 (*n* = 213 data points), ovarian *n* = 18 (*n* = 189 data points), pancreas *n* = 19 (*n* = 218 data points). These sum up to *n* = 90 independent PDX with *n* = 1,045 data points (or sampling events). Panels (**C**) and (**D**) show the UMAP of data points used in panels (**A**) and (**B**), respectively generated through unsupervised analysis. In terms of separation of data points (or mass spectral data similarity) the UMAP results mirror those presented in panels A and B using supervised analysis with more mixing of data in the permutated model.

To further support the statements above, expanding the discussion, Fig. 1A shows the ‘molecular model’ for lymphoma recognition created with PCA-LDA that formed the basis for the classifier list (i.e., important mass-to-charge values to distinguish solid tumours from lymphoproliferative outgrowths) provided in Table S1. This multivariate model uses *n* = 269 mass spectra from *n* = 20 independent lymphocytic outgrowths and aims to distinguish them from the PIRL-MS profiles of *n* = 90 common independent epithelioid solid tumour PDXs (*n* = 1,045 mass spectra), utilizing a total of 1,314 data points. The ‘control’ epithelioid PDX tumours represented those of colon, head & neck, lung, ovarian and pancreas origins, commonly generated for various preclinical studies at our site. Consistent with the separation seen in the Fig. 1A model between lymphocytic outgrowths and solid tumour PDX control cohort, Table 1’s cross-validation statistics predict 100% accuracy using a 5-fold leave-out test. Here, as no decision will be made based on unclassifiable predictions, they were removed from our calculations of accuracy. Nevertheless, only *n* = 68 sampling attempts out of *n* = 1,314 total spectra resulted in no predictions. This confirms a ~ 95% duty cycle for the method with only ~ 5% of sampling attempts resulting in no predictions (likely because of data quality and/or heterogeneity at the sampling site). It must be emphasized that our hand-held sampling approach uses visual guidance on the mesoscale (0.5–1.0 mm range) and as such it is possible that a given sampling event reports data from e.g., blood vessel, a stroma rich region or even murine skin among other forms of gross tissue heterogeneity. While spatially resolved mass spectrometry imaging methods exist to better correlate mass spectra to various forms of intrinsic specimen heterogeneity using histologic guidance, these methods require tissue sectioning, which significantly increase the turnaround time. Therefore, our approach relies on performing multiple sampling events across each specimen’s surface to capture as much of the intraspecimen heterogeneity as possible during classifier building. However, as described above, this approach is not without flaws, and ~ 5% of all sampling attempts result in unclassifiable data.

**Table 1:** Cross-validation accuracy assessment of Fig. 1A PCA-LDA model.

Data point groups						Correct classification rate	
Group	PIRL-MS spectra data points	Correctly classified data points	Misclassified data points	Unclassifiable data points	Classifiable data points	Per classifiable data points (%)	Per all (classifiable and unclassifiable) data points (%)
1	285	270	0	15	270	100.00	94.74
2	269	254	0	15	254	100.00	94.42
3	249	239	0	10	239	100.00	95.98
4	253	231	0	22	231	100.00	91.30
5	258	252	0	6	252	100.00	97.67
**Total**	**1314**	**1246**	**0**	**68**	**1246**	**100.00**	**94.82**

	Lymphoma	PDX tumour	Unclassifiable	Total			
Lymphoma	260	0	9	**269**			
PDX tumour	0	986	59	**1045**			
**Total**	**260**	**986**	**68**	**1314**			

Here, we show the results of a 5-fold leave-out cross-validation test wherein 5 data groups chosen to iteratively form 5 PCA-LDA models (using 80% of data) scoring them using the rest 20% of the data as a test set. The analysis used AMX^34^ ‘full-group’ leave-out test that ensures dependent measurements from a given specimen are only scored against other independent specimen data. As can be seen here, out of the total of *n* = 1,314 data points in Fig. 1A model, *n* = 1,246 were correctly classified, none were misclassified and only *n* = 68 data points fell outside user-determined model’s standard deviation to render classification. Further opportunities exist to reduce the unclassified data points, improving the duty cycle from its current ~ 95%, calculated from the ratio of unclassifiable (and bad data points if applicable, in this case a total of *n* = 68) to total sampling data points (*n* = 1,314). However, this was not pursued in our work here due to the satisfactory performance and the already reasonable duty cycle of the method without further optimization.

Significance value bold.

**Table 2:** Cross-validation accuracy assessment of the permutated Fig. 1B PCA-LDA model.

Data point groups						Correct classification rate	
Group	PIRL-MS spectra data points	Correctly classified data points	Misclassified data points	Unclassifiable data points	Classifiable data points	Per classifiable data points (%)	Per all (classifiable and unclassifiable) data points (%)
1	283	143	140	0	283	50.53	50.53
2	259	131	128	0	259	50.58	50.58
3	255	110	139	6	249	44.18	43.14
4	261	128	133	0	261	49.04	49.04
5	256	126	130	0	256	49.22	49.22
**Total**	**1314**	**638**	**670**	**6**	**1308**	**48.78**	**48.55**

	Lymphoma	PDX tumour	Unclassifiable	Total			
Lymphoma	300	365	5	**670**			
PDX tumour	305	338	1	**644**			
**Total**	**605**	**703**	**6**	**1314**			

The permutated model was created using the same total *n* = 1,314 now being split into two groups of ~ equal representation of lymphocytic and true solid tumour PDX groups (*n* = 55 independent specimens per group with *n* = 670 and *n =* 644 data points each). Interestingly, this model only possessed *n* = 6 unclassifiable data and false annotations significantly increased the number of misclassified data points from *n* = 0 in the true class model (Fig. 1A; Table 1) to *n* = 670 which is more than half of the total dataset. Consistent with this, and in keeping with the mixing of data points seen in Fig. 1B model, the 5-fold cross-validation accuracy of this permutated model was reduced from 100% (Fig. 1A; Table 1) to 48.78%, averaged across each of the 5-fold iterations of the leave-out test. Here, 5 data groups are formed (each accounting for 20% of data). Here, if a sampling event from a given group is included in the training set, all other events from the same specimens therein are excluded from the test set. The dramatic reduction in model’s ‘diagnostic power’ between true and false annotations strongly suggests that the multivariate model in Fig. 1A is likely robust in its ability to distinguish lymphocytic outgrowths from true solid tumour PDXs.

Significance value bold.

To ensure that the model predictions are significant, in Fig. 1B we show a permutated version of the panel A model in which we have used false annotations to create a 2-class model with each class possessing a mixture of data points across both lymphocytic and true PDX classes. As can be seen in Fig. 1B panel, the two classes show mixing of data points (albeit, with the distal most distinct data points being stretched out by the supervised linear discriminant analysis). Consistent with this, Table 2 shows poorer performance in class separation for this permutated model with 5-fold full group leave out cross-validation accuracy dropping to 48.78% from 100% for Panel A model bearing true annotations (as described in Table 1). This suggests that the statistical discrimination between lymphocytic outgrowths and true PDX specimens in Panel A model is likely robust and uninfluenced by ‘overfitting data’ and/or other confounding or unforeseen factors in data modeling. Consistent with this, unsupervised analysis of data using a UMAP algorithm for Fig. 1A, and Fig. 1B models shows the same patterns, with Fig. 1C (model with true annotations) showing distinction between lymphoma and PDX data and Fig. 1D (corresponding to permutated model of Fig. 1B) indeed possessing mixed data points between the two permutated data groups.

To further scrutinize the performance of the Fig. 1A model we performed blind sample evaluation using an additional *n* = 148 specimens (*n* = 2,079 spectral data points) with the breakdown of *n* = 30 lymphoma (*n* = 446 spectral data points) and *n* = 118 true PDX across lung, ovarian, head & neck, colon, pancreas, and additional two types of mesothelial and esophageal types not included in the model to assess its ‘generalizability’ (total *n* = 1,633 spectral data points). Table S2 summarizes the prediction classification results for each of these data points alongside signal duration (in seconds),  signal intensity and confidence in probability prediction and a metric for spatially concordant correct classification. This metric is of paramount importance to evaluate for practical reasons. A suitable molecular model must account for spatial heterogeneity in the signal, ensuring that concordant measurements are made and used for predictions, irrespective of the location of laser sampling on PDX’s surface. This dataset resulted in (98 ± 2)% spatially concordant classification (expressed as average ± 0.5 standard deviation), which is in line and slightly improved compared to previous reports of PIRL-MS sampling that utilized (more heterogeneous) human tissues^25–27,29^. As can be seen in Table S2, out of the *n* = 2,079 sampling attempts, *n* = 115 resulted in no predictions (unclassified data), and *n* = 34 data points did not result in good data. This translates to a practical duty cycle of ~ 93% (the meaning being 93% of the attempts result in classification readout). This is in line with the duty cycle of ~ 95% seen from the cross-validation of the Fig. 1A model itself discussed above in Table 1. As stated, only *n* = 34 attempts (or over 1.5% of the sampling attempts resulted in insufficient (or “bad”) data not possessing a signal duration or intensity to reliably generate “good quality” mass spectra. As such, the duty cycle of PIRL-MS method is favourable, with most attempted sampling efforts resulting in classification. This assessment uses an average signal duration (± 0.5 standard deviation) value of 10±1 s, which is extremely fast compared to the current gold standard histology-based or genomic sequencing methods. Of note, the average confidence in prediction probability (calculated from Mahalanobis distance mapping^35^ for the unknown dataset was (98 ± 2)%, calculated as the average with a ± 0.5 standard deviation. This supports the anticipated robustness of the method.

To add more precision to the arguments above, we converted the prediction statistics of the classifiable data points in Table S2 (we excluded unclassifiable/bad data based on which no decision is made, only affecting the duty cycle) to formal sensitivity and specificity values using standard definitions. Table 3 summarizes the sensitivity and specificity for 10-second identification of lymphocytic outgrowths from true solid tumour PDXs using PIRL-MS.

**Table 3:** The sensitivity and specificity values for blind test validation of 10-second PIRL-MS in distinguishing parasitic lymphocytic outgrowths from true solid tumour PDX engraftments in mice.

		Predicted class	
		+	−
Lymphoma			
Actual	+	410	3
		99.27%	
	−	14	1503
			99.08%
PDX tumour			
Actual	+	1503	14
		99.08%	
	−	3	410
			99.27%

For this assessment we used the favourable Fig. 1A PCA-LDA model (See table 1 cross-validation results) that successfully passed the permutation test and attempted to predict the PDX status of *n* = 148 independent specimens (*n* = 2,079 spectral data points) comprised of *n* = 30 lymphoma (*n* = 446 spectral data points) and *n* = 118 true PDX across lung, ovarian, head & neck, colon pancreas, mesothelial and esophageal types (forming *n* = 1,633 spectral data points). The breakdowns were as follows: *n* = 39 lung PDX (*n* = 634 data points), *n* = 20 ovarian PDX (*n* = 204 data points), *n* = 20 pancreas PDX (*n* = 280 data points), *n* = 18 colon PDX (*n* = 231 data points), *n* = 14 head & neck PDX (*n* = 197 data points), *n* = 3 esophageal PDX (*n* = 43 data points) and *n* = 4 mesothelial (*n* = 44 data points). The operator of PIRL-MS was blinded to the true class assignment of the unknown specimens to avoid any bias. Table S2 summarizes the predictions results from Mahalanobis method^35^ on AMX^34^ as done previously in PIRL-MS research^25,26,29,30^ for each sampling attempt. Translating the output of Table S2 to sensitivity and specificity values using standard definitions of true positive and true negative rates, 10-second PIRL-MS analysis is capable of high (> 99%) sensitivity and specificity detection of lymphocytic corruptions from true solid tumour PDX formations. This observation was made for several PDX models, over *n* = 1,930 classifiable data points. Here, a total of *n* = 2,079 sampling events were attempted and *n* = 115 of these resulted in unclassifiable data and *n* = 34 in bad data (*n* = 23 with signal duration less than or equal to 3 s and *n* = 11 data points not possessing lock-mass peak) as previously established^25^.

## Discussion, caveats, and mitigation strategies

As shown here, rapid identification of parasitic lymphocytic outgrowth during PDX generation is possible with sensitivity and specificity of > 99%, an observation made over close to 2,000 data points (1,930 to be exact) in a blind manner. Of particular interest, the Fig. 1A model is generalizable and able to correctly classify two novel types of PDX from mesothelial and esophageal origins not included in the model (i.e., training set). Coupled with previous demonstrations of the suitability of PIRL-MS sampling for *in vivo*^32^ or *in situ*^29,32^ exploration of murine models with limited laser induced tissue damage outside the ablated zone (~ 0.5 mm^3^ per shot), we believe an opportunity for translation of the hand-held sampling tool reported by our group for the analysis of murine models with 10-second PIRL-MS^32^ exists to streamline fidelity assessments in PDX generation workflows. Further analysis of the *n* = 14 misclassified specimens (*n* = 11 PDX and *n* = 3 lymphoma) reviewed by a pathologist did not uncover any obvious gross histologic heterogeneity not captured in the model. Therefore, additional opportunities to further improve performance, achieving near perfect discrimination between lymphocytic and true PDX growth may be possible through further scrutiny of these underperforming specimens from the molecular point of view, which we begin to consider below. Nevertheless, even in the absence of such scrutiny, the high sensitivity and specificity identification of lymphocytic outgrowths with 10-second PIRL-MS analysis is possible and this motivates future work to further reduce the cost and footprint of the hardware platform to facilitate wide adoption and routine use. An advantage of the current histology-based or sequencing-based methods to distinguish imposter tumours is their widespread availability and relatively low operational (running) costs. While the running cost of PIRL-MS platform is also low and perhaps comparable, the high capital cost of equipment and its uniqueness (general unavailability elsewhere) are currently limiting its immediate term translational potential. Nevertheless, we believe that the encouraging performance results provided in this manuscript could be leveraged to motivate follow-on work to (1) reduce the cost and footprint of the platform and to (2) further perform more rigorous analytical performance validation as summarized previously^28,38,39^. These include multisite validation of performance reproducibility, generalizability of the classifier model for PDXs created elsewhere, and assessment of data variability due to variance in user (operator) performance. To this end, our laboratory has developed and published guidelines for analytical performance validation^39^, analytical reproducibility assessments^38^, and model generalizability^28^, to guide such future follow-on efforts. It is thus at the conclusion of these developments that PIRL-MS could widely contribute to improved quality control in PDX generation workflows.

In addition to the caveats listed above, our work fails to address a fundamental scientific question: why does PIRL-MS work as well as it does in distinguishing lymphoproliferative outgrowths? In other words, what are the molecular drivers that separate the mass spectrum of an imposter lymphoproliferative tumour from that of a solid tumour of epithelioid origin? While Table S1 reports the features (*m/z* values) that are important for said classification, the identities of these molecules remain unknown, requiring high-resolution tandem mass spectrometry after chromatography as we have performed for other cancer cases^25,26,29–31^. To shed more light, however, we draw attention to a parallel manuscript (in submission) for which we have collected and assigned molecular identities to PIRL-MS spectra of primary lymphoma and various solid tumours, including several of epithelioid origin for scientific purposes unrelated to the work in this manuscript^40^. A portion of this data, if subjected to remodelling in forms not presented therein, provides an opportunity to glean insights into molecular drivers of difference between lymphoma and solid tumours, albeit in humans. We have, therefore, taken the opportunity to repurpose the said data, reanalyzed it in a way not discussed in the pending manuscript^40^ to comment on the identities of the molecular drivers of statistical discrimination between lymphoproliferative lesions and solid epithelioid tumours. Utilizing human tissues to address this question alleviates the need to account for non-negligible contributions of murine stroma to PIRL-MS signals and facilitates data interpretability in the absence of otherwise necessary controls to recognize and exclude murine stromal contributions from human cell signals. To this end, Fig. S2 shows a 2-way PCA-LDA model comprised of *n* = 22 human lymphoma and *n* = 22 human epithelioid tumours of same origin as those in Fig. 1A. Fig. S3 shows the ‘loading plot’ associated with said PCA-LDA model (of Fig. S2) indicating ions important for the differentiation between lymphoma and solid tumours of epithelioid origin. Among these, the pending manuscript in submission ^40^ identifies and reports six ions belonging to several classes of lipids namely ceramides, phosphatidylcholines, phosphatidylethanolamines, and sphingomyelins (See the legend accompanying Fig. S3). Therefore, it appears that differences in lipid metabolism likely contribute to the observed differentiation. This is consistent with the role of lipid metabolism alterations in other cancer type differentiations reported for PIRL-MS ^25,26,29–31^ and is further in keeping with fundamental science insights that lipid metabolism is altered in tumorigenesis^36,37,41–44^.

Lastly, we acknowledge that all analysis was performed on a proprietary software, AMX (Waters Research Centre, Budapest). While this platform may not be available to all users for academic use, in Fig. S4 we confirm that an in-house script based on Python can reproduce Fig. 1A PCA-LDA model with similar extent of separation between the two groups and substantially equivalent performance using a 5-fold leave-out test as shown in Table S3. Our house-built code further allows additional insights into the ‘degree of mixing’ between signals of the lymphoproliferative and solid tumours. Building on the substantial equivalency established between Python-based and AMX modeling (demonstrated by Fig. S4/Table S3 results), Fig. S5 shows a ‘jitter plot’ created based on Fig. S4 PCA-LDA plot, a feat which is not achievable on AMX due to its intended rigid design. Interpreting the results of degree of mixing allows further scientific insight into heterogeneities of PIRL-MS signal, opening new avenues for data analysis not possible in ‘projection-based modeling’ on AMX shown in Fig. 1A. This flexibility will further enable future users to access additional classifiers associated with signal variability for optimizations if deemed necessary. Overall, considering these concordant results, we do not believe access to proprietary data analytics platform such as AMX is hindering future adoption, and this is an important point to highlight at the conclusion of this manuscript. In a similar vein, reliance on in-house code enables several other advantages. First, it enables use of alternate data analysis methods not available on AMX to address the potential impact of imbalanced datasets on classification/cross-validation accuracies. This is demonstrated in Table S4 confusion matrix where we have compared the results of 5-fold cross-validation between PCA-LDA (in Fig. S4, results shown in Table S3) and PCA-LDA coupled with Synthetic Minority Over-sampling Technique (SMOTE), to make the classifier more suitable for imbalanced data analysis. The limited difference in cross-validation results between Fig. S4 (results shown in Table S3) and those in Table S4 with SMOTE augmentation to our PCA-LDA model suggests that despite utilizing an imbalanced dataset comprised of ~ 4-fold more data points for PDX tumours compared to lymphoproliferative lesions, the models presented and verified in this manuscript remain valid for future use.

Second, the use of this versatile code developed in house further enables an assessment of ‘threshold independent performance metrics’. In this context, Fig. S6 illustrates Receiver Operating Characteristic (ROC) and Area Under the [ROC] Curve (AUC) for Fig. S4 PCA-LDA plot created in Python (Fig. S6A) along its SMOTE variant (Fig. S6B). AMX uses PCA-LDA modeling in combination with a threshold dependent method of performance assessment (e.g. Accuracy, sensitivity, specificity). Area under ROC analysis, on the other hand, provides a threshold-independent way of assessing model performance. Namely, the constraints of a ‘static’ confusion matrix do not influence the ROC analysis. The concordance between ROC and multivariate cross-validation results further validates the robustness of the models developed in this work.

Third, the use of Python allows for a more detailed assessment of mass spectral properties, such as feature-based correlation maps, to glean insights into the origin of misclassified data. Fig. S7 shows the correlation maps for 14 misclassified data alongside ground truth (correctly classified 134 unknowns, comprised of 27 lymphomas and 107 PDX solid tumours), wherein, despite a lack of gross histologic alterations (from H&E analysis), subtle mass spectral changes are shown to drive misclassifications. Here, lymphoma-like correlation maps were detected for unknowns 3, 12, 29, 36, 38 104 and 146. These specimens were solid PDX tumours that had some of their sampling events misclassified as lymphoma. In a similar vein, unknown 141 which was a lymphoma, had qualitative PDX-like features populating its correlation map and hence, had one of its sampling events misclassified as such. While the correlation maps were informative to potentially explain some of the misclassifications on grounds of spectral similarities to the misclassifying type, they failed to rationalize why unknowns 9, 45, 51, 90, 93 and 107 also resulted in misclassifications. The correlation maps for these unknowns were drastically different from the control maps created from correctly classified unknowns. Here, a more fundamental question to be addressed is why the mass spectra of some of the misclassified unknowns possessed said features to promote misclassifications? In other words, while we can rationalize why the presence of certain features in correlation maps could explain the misclassifications, we cannot provide any insights as to why said unknowns produced such features characteristic of the misclassified tumour types. Having said this, it is worth noting that most of the sampling events from such unknowns formed unclassifiable outliers upon classification against the model. This is a positive attribute that highlights the model’s overall success in recognizing difficult-to-classify specimens.

## Supplementary Information

Below is the link to the electronic supplementary material.

Supplementary Material 1

## Data Availability

To access raw mass spectral data University Health Network must be contacted. Please email the corresponding author “ [arash.zarrine.afsar@utoronto.ca](mailto: arash.zarrine.afsar@utoronto.ca) ” to facilitate the discussion.
