# Supplementary material for: Picosecond infrared laser mass spectrometry for 10-second identification of lymphoproliferative imposter tumours in patient-derived xenografts
Source: Sci Rep. 2026 Jan 7;16:3198. doi: 10.1038/s41598-025-33064-w (PMC12830993; doi:10.1038/s41598-025-33064-w)
Supplement: Supplementary file 1 — Supplementary Material 1 [file 41598_2025_33064_MOESM1_ESM.pdf]

Supplementary Information

Picosecond infrared laser mass spectrometry for 10-second identification of lymphoproliferative imposter tumours in patient-derived xenografts

Lan-Anna Ye[1], Darah Vlaminck [1,2], Alexa Fiorante[1,2], Laurentiu G. Dabija[1,2], Francis Talbot[1], Julia Froment[1], Alhareth Azaizeh[1,3], Likun Hou[1], Ming Li[1], Yuhui Wang[1], Pinjiang Cao[1], Dani Shouk[1], Rani Shouk[1], Ming-Sound Tsao[1], Laurie Ailles[1,2], Catherine O'Brien[1,2], Benjamin H. Lok[1], Nhu-An Pham[1] and Arash Zarrine-Afsar[1,2,4,5,*]

[1] Princess Margaret Cancer Centre, University Health Network, 101 College Street, Toronto, ON, M5G 1L7, Canada
[2] Department of Medical Biophysics, University of Toronto, 101 College Street, Toronto, ON, M5G 1L7, Canada
[3] Present address: Jordanian Royal Medical Services, Amman, Jordan
[4] Department of Surgery, University of Toronto, 149 College Street, Toronto, ON, M5T 1P5, Canada
[5] Keenan Research Center for Biomedical Science & the Li Ka Shing Knowledge Institute, St. Michael's Hospital, 30 Bond Street, Toronto, ON, M5B 1W8, Canada

*Corresponding author: Arash Zarrine-Afsar
Email: arash.zarrine.afsar@utoronto.ca
101 College Street, Room 7-207,
MaRS Building, Princess Margaret Cancer Research Tower, 7th floor (STTARR)
Toronto, ON M5G 1L7
TEL (Office): ++1-416-581-8473

Conflict of interest statement

AZA is a co-inventor of 'soft ionization' utilized in this study and is consultant with Point Surgical Inc. with financial interest.

Author contributions statement

Study design: L-AY, AZA, N-AP
Acquisition, curation, analysis & interpretation of data: L-AY, DV, DS, RS, LD, AF, FT
Drafting the manuscript: AZA
Access to resources: JF, AA, LH, ML, YW, PC, M-ST, LA, CO'B, BL, N-AP
Editing the manuscript: All authors

Running title

10-second determination of lymphoproliferative PDXs

Table of content

Table S1……………………………………………………………………………………………………..Pages S3-S4
Table S2……………………………………………………………………………………………………..Pages S5-S55
Table S3……………………………………………………………………………………………………...Page S55
Table S4……………………………………………………………………………………………………...Page S55
Figure S1……………………………………………………………………………………………………..Page S56
Figure S2……………………………………………………………………………………………………..Page S57
Figure S3……………………………………………………………………………………………………..Page S57
Figure S4……………………………………………………………………………………………………..Page S58
Figure S5……………………………………………………………………………………………………..Page S58
Figure S6……………………………………………………………………………………………………..Page S59
Figure S7……………………………………………………………………………………………………..Page S60
References…………………………………………………………………………………………………...Page S61

Top 100 Binned m/z	
125.05	698.55
128.05	699.55
134.05	701.55
170.05	703.55
187.05	709.55
214.05	710.65
220.05	711.45
238.05	713.45
241.15	716.45
251.15	716.55
307.25	717.55
311.15	726.55
325.15	733.45
329.25	733.55
331.25	734.65
337.15	735.45
355.35	736.65
375.15	737.45
377.25	737.65
381.35	739.55
403.25	740.65
441.25	744.55
443.25	754.55
467.25	763.45
524.45	766.55
570.45	766.65
572.55	768.55
576.45	769.55
603.45	770.55
624.55	772.65
642.45	779.65
642.55	793.55
653.45	795.55
658.55	795.65
660.55	796.55
663.55	814.65
665.55	818.75
667.55	824.65
668.55	826.65
671.45	827.65
677.45	828.65
679.45	829.65
681.55	832.65

682.65	885.55
685.55	886.55
686.55	891.75
689.45	901.75
691.45	915.75
691.55	917.75
693.45	928.85

Table S1. The top 100 most important *m/z* values for PDX type differentiation. This table lists the top 100 *m/z* features from analysis of the 'loading plots' of Fig. 1A PCA-LDA model binned to 0.1 Da. The list is comprised of 50 most positively and 50 most negatively discriminating *m/z* values across linear discriminant 1 (LD1). The fraction of the identified ions from the top 100 *m/z* features that are visible on the representative spectra (binned values) in Fig. S1 is indicated in red font on Fig. S1.

Specimen ID	True Class	Duration of Signal (seconds)	Total Ion Count (TIC)	PCA-LDA Prediction (AMX Recognition)	Probability	% Spatially Invariant Correct Classification
**Unknown 1**	PDX tumour	13	5.67E+06	PDX tumour	99.28	100.00
**Unknown 1**	PDX tumour	11	2.96E+06	PDX tumour	99.33	
**Unknown 1**	PDX tumour	12	3.37E+06	PDX tumour	99.37	
**Unknown 1**	PDX tumour	13	2.53E+06	PDX tumour	99.37	
**Unknown 1**	PDX tumour	14	1.88E+06	PDX tumour	99.36	
**Unknown 1**	PDX tumour	12	1.02E+07	PDX tumour	99.33	
**Unknown 1**	PDX tumour	12	3.28E+06	PDX tumour	99.37	
**Unknown 1**	PDX tumour	13	5.20E+06	PDX tumour	99.36	
**Unknown 1**	PDX tumour	1	2.92E+05	bad data	-	
**Unknown 1**	PDX tumour	12	3.24E+06	PDX tumour	99.34	
**Unknown 1**	PDX tumour	11	2.15E+06	PDX tumour	99.39	
**Unknown 1**	PDX tumour	10	2.09E+06	PDX tumour	99.39	
**Unknown 1**	PDX tumour	12	3.44E+06	PDX tumour	99.39	
**Unknown 2**	PDX tumour	11	6.16E+06	PDX tumour	99.04	100.00
**Unknown 2**	PDX tumour	12	5.55E+05	PDX tumour	99.02	
**Unknown 2**	PDX tumour	11	2.08E+06	PDX tumour	99.15	
**Unknown 2**	PDX tumour	11	2.70E+06	PDX tumour	99.3	
**Unknown 2**	PDX tumour	2	7.96E+05	bad data	-	
**Unknown 2**	PDX tumour	1	1.78E+05	bad data	-	
**Unknown 2**	PDX tumour	10	7.97E+06	PDX tumour	99.34	
**Unknown 2**	PDX tumour	10	2.23E+06	PDX tumour	99.36	
**Unknown 2**	PDX tumour	7	9.52E+05	PDX tumour	99.05	
**Unknown 2**	PDX tumour	10	1.09E+06	PDX tumour	99.1	
**Unknown 2**	PDX tumour	11	2.57E+06	PDX tumour	98.92	
**Unknown 2**	PDX tumour	8	3.34E+05	PDX tumour	99.13	
**Unknown 2**	PDX tumour	10	2.32E+06	PDX tumour	99.32	
**Unknown 2**	PDX tumour	9	7.33E+06	PDX tumour	99.39	
**Unknown 2**	PDX tumour	8	2.23E+06	PDX tumour	97.97	
**Unknown 2**	PDX tumour	6	1.28E+06	Unclassifiable	-	
**Unknown 2**	PDX tumour	9	1.53E+06	PDX tumour	99.37	
**Unknown 2**	PDX tumour	10	2.36E+06	PDX tumour	99.39	
**Unknown 2**	PDX tumour	7	7.52E+05	PDX tumour	99.03	
**Unknown 2**	PDX tumour	11	1.49E+06	PDX tumour	99.27	
**Unknown 2**	PDX tumour	10	1.68E+06	PDX tumour	99.38	
**Unknown 2**	PDX tumour	9	1.31E+06	PDX tumour	99.38	
**Unknown 3**	PDX tumour	11	1.82E+06	PDX tumour	99.05	90.91
**Unknown 3**	PDX tumour	12	2.75E+06	PDX tumour	98.02	
**Unknown 3**	PDX tumour	13	3.11E+06	PDX tumour	98.04	

**Unknown 3**	PDX tumour	11	2.08E+06	PDX tumour	99.34	
**Unknown 3**	PDX tumour	14	4.49E+06	PDX tumour	98.3	
**Unknown 3**	PDX tumour	12	4.64E+06	Unclassifiable	-	
**Unknown 3**	PDX tumour	13	4.60E+06	PDX tumour	99.04	
**Unknown 3**	PDX tumour	11	4.01E+06	Unclassifiable	-	
**Unknown 3**	PDX tumour	12	4.57E+06	Unclassifiable	-	
**Unknown 3**	PDX tumour	13	4.43E+06	Unclassifiable	-	
**Unknown 3**	PDX tumour	14	2.88E+06	Lymphoma	88.44	
**Unknown 3**	PDX tumour	15	2.65E+06	Unclassifiable	-	
**Unknown 3**	PDX tumour	7	1.97E+06	PDX tumour	99.09	
**Unknown 3**	PDX tumour	11	5.30E+06	PDX tumour	98.84	
**Unknown 3**	PDX tumour	13	5.33E+06	Unclassifiable	-	
**Unknown 3**	PDX tumour	11	4.98E+06	PDX tumour	99.17	
**Unknown 3**	PDX tumour	15	6.01E+06	Unclassifiable	-	
**Unknown 3**	PDX tumour	12	8.15E+06	Unclassifiable	-	
**Unknown 3**	PDX tumour	11	4.67E+06	Unclassifiable	-	
**Unknown 3**	PDX tumour	12	5.49E+06	PDX tumour	98.98	
**Unknown 4**	PDX tumour	14	2.61E+06	PDX tumour	99.38	100.00
**Unknown 4**	PDX tumour	11	1.81E+06	PDX tumour	99.39	
**Unknown 4**	PDX tumour	10	1.63E+06	PDX tumour	99.38	
**Unknown 4**	PDX tumour	12	2.40E+06	PDX tumour	99.35	
**Unknown 4**	PDX tumour	11	2.40E+06	PDX tumour	99.38	
**Unknown 4**	PDX tumour	14	2.61E+06	PDX tumour	99.37	
**Unknown 4**	PDX tumour	10	2.46E+06	PDX tumour	99.33	
**Unknown 4**	PDX tumour	12	2.71E+06	PDX tumour	99.37	
**Unknown 4**	PDX tumour	12	2.06E+06	PDX tumour	99.38	
**Unknown 4**	PDX tumour	12	2.63E+06	PDX tumour	99.38	
**Unknown 4**	PDX tumour	10	1.51E+06	PDX tumour	99.38	
**Unknown 4**	PDX tumour	11	4.40E+06	PDX tumour	99.38	
**Unknown 4**	PDX tumour	14	3.82E+06	PDX tumour	99.31	
**Unknown 4**	PDX tumour	14	3.06E+06	PDX tumour	99.28	
**Unknown 4**	PDX tumour	13	3.58E+06	PDX tumour	99.38	
**Unknown 5**	PDX tumour	13	3.97E+06	PDX tumour	99.33	100.00
**Unknown 5**	PDX tumour	12	3.16E+06	PDX tumour	99.39	
**Unknown 5**	PDX tumour	11	2.38E+06	PDX tumour	99.35	
**Unknown 5**	PDX tumour	12	2.25E+06	PDX tumour	99.38	
**Unknown 5**	PDX tumour	11	3.48E+06	PDX tumour	99.36	
**Unknown 5**	PDX tumour	11	4.33E+06	PDX tumour	99.15	
**Unknown 5**	PDX tumour	12	2.26E+06	PDX tumour	98.96	
**Unknown 5**	PDX tumour	10	3.43E+06	PDX tumour	99.05	
**Unknown 5**	PDX tumour	9	3.14E+06	PDX tumour	99.13	
**Unknown 5**	PDX tumour	10	2.66E+06	PDX tumour	99.27	

**Unknown 6**	PDX tumour	8	1.69E+06	PDX tumour	99.36	100.00
**Unknown 6**	PDX tumour	10	2.08E+06	PDX tumour	99.35	
**Unknown 6**	PDX tumour	11	2.10E+06	PDX tumour	99.35	
**Unknown 6**	PDX tumour	10	2.04E+06	PDX tumour	99.37	
**Unknown 6**	PDX tumour	9	2.62E+06	PDX tumour	99.29	
**Unknown 6**	PDX tumour	10	2.95E+06	PDX tumour	99.31	
**Unknown 7**	PDX tumour	9	3.57E+06	PDX tumour	99.39	100.00
**Unknown 7**	PDX tumour	9	4.25E+06	PDX tumour	99.39	
**Unknown 7**	PDX tumour	9	3.93E+06	PDX tumour	99.38	
**Unknown 7**	PDX tumour	10	3.49E+06	PDX tumour	99.36	
**Unknown 7**	PDX tumour	9	4.33E+06	PDX tumour	99.37	
**Unknown 7**	PDX tumour	10	4.01E+06	PDX tumour	99.36	
**Unknown 7**	PDX tumour	10	4.27E+06	PDX tumour	99.38	
**Unknown 7**	PDX tumour	10	3.77E+06	PDX tumour	99.35	
**Unknown 7**	PDX tumour	9	3.42E+06	PDX tumour	99.38	
**Unknown 7**	PDX tumour	8	2.31E+06	PDX tumour	99.36	
**Unknown 7**	PDX tumour	13	2.17E+06	PDX tumour	99.37	
**Unknown 7**	PDX tumour	7	2.47E+06	PDX tumour	99.39	
**Unknown 8**	PDX tumour	11	7.06E+05	PDX tumour	99.34	100.00
**Unknown 8**	PDX tumour	9	6.65E+05	PDX tumour	99.21	
**Unknown 8**	PDX tumour	7	1.51E+06	PDX tumour	99.38	
**Unknown 8**	PDX tumour	5	6.90E+05	PDX tumour	99.32	
**Unknown 8**	PDX tumour	10	1.39E+06	PDX tumour	99.24	
**Unknown 8**	PDX tumour	4	4.07E+05	PDX tumour	99.35	
**Unknown 9**	Lymphoma	12	2.96E+06	Lymphoma	99.28	92.86
**Unknown 9**	Lymphoma	11	4.08E+06	Lymphoma	99.35	
**Unknown 9**	Lymphoma	10	4.62E+06	Lymphoma	99.28	
**Unknown 9**	Lymphoma	12	2.46E+06	Lymphoma	99.38	
**Unknown 9**	Lymphoma	12	4.45E+06	Lymphoma	99.37	
**Unknown 9**	Lymphoma	13	4.39E+06	Lymphoma	94.62	
**Unknown 9**	Lymphoma	11	4.07E+06	PDX tumour	98.59	
**Unknown 9**	Lymphoma	12	6.39E+06	Lymphoma	99.29	
**Unknown 9**	Lymphoma	12	3.77E+06	Lymphoma	99.28	
**Unknown 9**	Lymphoma	12	4.88E+06	Lymphoma	99.35	
**Unknown 9**	Lymphoma	11	2.47E+06	Lymphoma	99.39	
**Unknown 9**	Lymphoma	13	1.32E+06	Lymphoma	99.38	
**Unknown 9**	Lymphoma	13	1.95E+06	Lymphoma	99.39	
**Unknown 9**	Lymphoma	7	1.81E+06	Lymphoma	99.39	
**Unknown 10**	PDX tumour	9	2.83E+06	PDX tumour	98.83	100.00
**Unknown 10**	PDX tumour	1	1.51E+05	bad data	-	
**Unknown 10**	PDX tumour	1	7.79E+04	bad data	-	
**Unknown 10**	PDX tumour	10	2.14E+06	PDX tumour	99.39	

**Unknown 10**	PDX tumour	11	2.58E+06	PDX tumour	99.36	
**Unknown 10**	PDX tumour	9	2.45E+06	PDX tumour	99.39	
**Unknown 10**	PDX tumour	13	1.95E+06	PDX tumour	99.37	
**Unknown 10**	PDX tumour	12	2.12E+06	PDX tumour	99.39	
**Unknown 10**	PDX tumour	8	9.94E+05	PDX tumour	99.02	
**Unknown 10**	PDX tumour	10	8.57E+05	PDX tumour	99.37	
**Unknown 10**	PDX tumour	11	1.21E+06	PDX tumour	99.16	
**Unknown 10**	PDX tumour	10	2.31E+06	PDX tumour	99.38	
**Unknown 10**	PDX tumour	10	3.22E+06	PDX tumour	99.38	
**Unknown 10**	PDX tumour	9	2.43E+06	PDX tumour	99.35	
**Unknown 10**	PDX tumour	9	1.72E+06	PDX tumour	99.12	
**Unknown 11**	Lymphoma	11	5.81E+06	Lymphoma	99.3	100.00
**Unknown 11**	Lymphoma	10	4.37E+06	Lymphoma	98.97	
**Unknown 11**	Lymphoma	9	5.06E+06	Lymphoma	98.97	
**Unknown 11**	Lymphoma	9	4.76E+06	Lymphoma	98.96	
**Unknown 11**	Lymphoma	9	3.08E+06	Lymphoma	98.6	
**Unknown 11**	Lymphoma	10	3.14E+06	Lymphoma	98.95	
**Unknown 11**	Lymphoma	10	3.85E+06	Lymphoma	99.38	
**Unknown 11**	Lymphoma	8	7.22E+05	Lymphoma	99.33	
**Unknown 11**	Lymphoma	7	2.86E+06	Lymphoma	99.19	
**Unknown 11**	Lymphoma	10	5.72E+06	Lymphoma	99.29	
**Unknown 11**	Lymphoma	8	3.66E+06	Lymphoma	98.6	
**Unknown 11**	Lymphoma	9	3.92E+06	Lymphoma	99.34	
**Unknown 11**	Lymphoma	10	4.24E+06	Lymphoma	99.27	
**Unknown 11**	Lymphoma	9	4.42E+06	Lymphoma	<u>94.94</u>	
**Unknown 11**	Lymphoma	10	3.23E+06	Lymphoma	96.54	
**Unknown 11**	Lymphoma	10	2.91E+06	Lymphoma	97.36	
**Unknown 11**	Lymphoma	9	2.68E+06	Lymphoma	98.69	
**Unknown 11**	Lymphoma	9	7.57E+06	Lymphoma	99.3	
**Unknown 12**	PDX tumour	13	4.02E+06	PDX tumour	99.04	83.33
**Unknown 12**	PDX tumour	11	3.51E+06	Unclassifiable	-	
**Unknown 12**	PDX tumour	12	3.13E+06	PDX tumour	98.23	
**Unknown 12**	PDX tumour	14	3.16E+06	Unclassifiable	-	
**Unknown 12**	PDX tumour	11	2.78E+06	PDX tumour	98.46	
**Unknown 12**	PDX tumour	10	2.67E+06	PDX tumour	98.84	
**Unknown 12**	PDX tumour	8	1.35E+06	PDX tumour	98.21	
**Unknown 12**	PDX tumour	10	1.42E+06	Lymphoma	<u>74.49</u>	
**Unknown 12**	PDX tumour	11	1.58E+06	Unclassifiable	-	
**Unknown 12**	PDX tumour	13	4.21E+06	Unclassifiable	-	
**Unknown 13**	PDX tumour	2	3.20E+05	bad data	-	100.00
**Unknown 13**	PDX tumour	1	1.61E+05	bad data	-	
**Unknown 13**	PDX tumour	9	1.22E+06	PDX tumour	99.3	

**Unknown 13**	PDX tumour	4	6.44E+05	PDX tumour	99.3	
**Unknown 13**	PDX tumour	15	5.87E+05	PDX tumour	99.01	
**Unknown 13**	PDX tumour	9	1.25E+06	PDX tumour	99.39	
**Unknown 13**	PDX tumour	15	1.98E+06	PDX tumour	99.36	
**Unknown 13**	PDX tumour	15	1.74E+06	PDX tumour	99.39	
**Unknown 13**	PDX tumour	15	1.52E+06	PDX tumour	99.39	
**Unknown 13**	PDX tumour	9	1.94E+06	PDX tumour	99.39	
**Unknown 13**	PDX tumour	8	5.65E+05	Unclassifiable	-	
**Unknown 13**	PDX tumour	12	2.25E+06	PDX tumour	99.35	
**Unknown 13**	PDX tumour	10	1.46E+06	PDX tumour	99.38	
**Unknown 14**	PDX tumour	12	1.31E+06	PDX tumour	99.36	100.00
**Unknown 14**	PDX tumour	12	9.34E+05	PDX tumour	99.39	
**Unknown 14**	PDX tumour	15	6.14E+05	PDX tumour	99.31	
**Unknown 14**	PDX tumour	12	1.64E+06	PDX tumour	99.34	
**Unknown 14**	PDX tumour	13	1.93E+06	PDX tumour	99.37	
**Unknown 14**	PDX tumour	11	1.65E+06	PDX tumour	99.39	
**Unknown 14**	PDX tumour	11	2.59E+06	PDX tumour	99.37	
**Unknown 14**	PDX tumour	13	2.89E+06	PDX tumour	99.39	
**Unknown 14**	PDX tumour	14	2.67E+06	PDX tumour	99.39	
**Unknown 14**	PDX tumour	10	1.96E+06	PDX tumour	99.37	
**Unknown 14**	PDX tumour	15	1.72E+06	PDX tumour	99.33	
**Unknown 14**	PDX tumour	10	2.27E+06	PDX tumour	99.37	
**Unknown 14**	PDX tumour	10	2.33E+06	PDX tumour	99.35	
**Unknown 14**	PDX tumour	10	2.31E+06	PDX tumour	99.38	
**Unknown 14**	PDX tumour	10	2.91E+06	PDX tumour	99.38	
**Unknown 14**	PDX tumour	15	2.73E+06	PDX tumour	99.37	
**Unknown 14**	PDX tumour	15	2.88E+06	PDX tumour	99.39	
**Unknown 14**	PDX tumour	11	1.87E+06	PDX tumour	99.38	
**Unknown 15**	PDX tumour	12	1.81E+06	PDX tumour	99.39	100.00
**Unknown 15**	PDX tumour	13	2.28E+06	PDX tumour	99.38	
**Unknown 15**	PDX tumour	13	3.59E+06	PDX tumour	99.34	
**Unknown 15**	PDX tumour	11	1.02E+06	PDX tumour	99.37	
**Unknown 15**	PDX tumour	12	1.03E+06	PDX tumour	99.38	
**Unknown 15**	PDX tumour	14	3.44E+06	PDX tumour	99.39	
**Unknown 15**	PDX tumour	11	2.05E+06	PDX tumour	99.38	
**Unknown 15**	PDX tumour	12	1.87E+06	PDX tumour	99.38	
**Unknown 15**	PDX tumour	12	3.60E+06	PDX tumour	99.38	
**Unknown 15**	PDX tumour	12	4.58E+06	PDX tumour	99.37	
**Unknown 15**	PDX tumour	12	2.33E+06	PDX tumour	99.36	
**Unknown 15**	PDX tumour	11	2.04E+06	PDX tumour	99.38	
**Unknown 15**	PDX tumour	10	2.96E+06	PDX tumour	99.38	
**Unknown 15**	PDX tumour	13	5.11E+06	PDX tumour	99.38	

**Unknown 15**	PDX tumour	11	2.18E+06	PDX tumour	99.38	
**Unknown 15**	PDX tumour	11	2.87E+06	PDX tumour	99.39	
**Unknown 15**	PDX tumour	10	1.76E+06	PDX tumour	99.38	
**Unknown 15**	PDX tumour	12	2.50E+06	PDX tumour	99.26	
**Unknown 15**	PDX tumour	10	2.01E+06	PDX tumour	99.37	
**Unknown 15**	PDX tumour	11	2.01E+06	PDX tumour	99.39	
**Unknown 15**	PDX tumour	12	1.49E+06	PDX tumour	99.38	
**Unknown 15**	PDX tumour	15	2.13E+06	PDX tumour	99.37	
**Unknown 15**	PDX tumour	15	1.18E+06	PDX tumour	99.39	
**Unknown 15**	PDX tumour	11	2.99E+06	PDX tumour	99.35	
**Unknown 15**	PDX tumour	12	2.49E+06	PDX tumour	99.36	
**Unknown 15**	PDX tumour	12	1.88E+06	PDX tumour	99.38	
**Unknown 15**	PDX tumour	11	1.18E+06	PDX tumour	99.39	
**Unknown 15**	PDX tumour	9	8.79E+05	PDX tumour	99.39	
**Unknown 15**	PDX tumour	10	2.13E+06	PDX tumour	99.39	
**Unknown 15**	PDX tumour	13	5.86E+06	PDX tumour	99.38	
**Unknown 15**	PDX tumour	9	7.16E+06	PDX tumour	99.32	
**Unknown 16**	PDX tumour	12	3.74E+06	PDX tumour	99.31	100.00
**Unknown 16**	PDX tumour	11	2.19E+06	PDX tumour	99.37	
**Unknown 16**	PDX tumour	15	4.15E+06	PDX tumour	99.38	
**Unknown 16**	PDX tumour	11	4.42E+06	PDX tumour	99.39	
**Unknown 16**	PDX tumour	13	2.71E+06	PDX tumour	99.38	
**Unknown 16**	PDX tumour	10	2.14E+06	PDX tumour	99.39	
**Unknown 16**	PDX tumour	13	3.12E+06	PDX tumour	99.35	
**Unknown 16**	PDX tumour	13	2.49E+06	PDX tumour	99.39	
**Unknown 16**	PDX tumour	11	1.13E+06	PDX tumour	99.32	
**Unknown 16**	PDX tumour	12	2.35E+06	PDX tumour	99.39	
**Unknown 16**	PDX tumour	15	4.22E+06	PDX tumour	99.37	
**Unknown 16**	PDX tumour	11	2.25E+06	PDX tumour	99.39	
**Unknown 16**	PDX tumour	7	1.93E+06	PDX tumour	99.38	
**Unknown 16**	PDX tumour	16	2.10E+06	PDX tumour	99.39	
**Unknown 16**	PDX tumour	7	1.40E+06	PDX tumour	99.39	
**Unknown 17**	PDX tumour	4	5.25E+05	PDX tumour	99.3	100.00
**Unknown 17**	PDX tumour	1	9.36E+04	bad data	-	
**Unknown 17**	PDX tumour	8	2.25E+06	PDX tumour	99.36	
**Unknown 17**	PDX tumour	9	1.50E+06	PDX tumour	99.38	
**Unknown 17**	PDX tumour	9	2.41E+06	PDX tumour	99.37	
**Unknown 17**	PDX tumour	8	9.49E+05	PDX tumour	99.25	
**Unknown 17**	PDX tumour	5	4.27E+05	PDX tumour	98.97	
**Unknown 17**	PDX tumour	12	9.16E+05	PDX tumour	99.14	
**Unknown 18**	Lymphoma	10	3.54E+06	Lymphoma	99.38	100.00
**Unknown 18**	Lymphoma	9	3.50E+06	Lymphoma	99.37	

**Unknown 18**	Lymphoma	9	2.78E+06	Lymphoma	99.38	
**Unknown 18**	Lymphoma	9	2.56E+06	Lymphoma	99.38	
**Unknown 18**	Lymphoma	9	2.68E+06	Lymphoma	99.39	
**Unknown 18**	Lymphoma	9	2.11E+06	Lymphoma	99.36	
**Unknown 18**	Lymphoma	9	2.66E+06	Lymphoma	99.38	
**Unknown 18**	Lymphoma	8	3.65E+06	Lymphoma	99.38	
**Unknown 18**	Lymphoma	9	3.48E+06	Lymphoma	99.38	
**Unknown 18**	Lymphoma	9	4.46E+06	Lymphoma	99.35	
**Unknown 18**	Lymphoma	10	4.73E+06	Lymphoma	99.36	
**Unknown 18**	Lymphoma	10	3.55E+06	Lymphoma	99.36	
**Unknown 18**	Lymphoma	11	4.96E+06	Lymphoma	99.3	
**Unknown 18**	Lymphoma	1	1.47E+05	bad data	-	
**Unknown 18**	Lymphoma	11	6.13E+06	Lymphoma	99.34	
**Unknown 18**	Lymphoma	9	4.90E+06	Lymphoma	99.34	
**Unknown 18**	Lymphoma	11	2.32E+06	Lymphoma	99.38	
**Unknown 19**	PDX tumour	10	4.00E+06	PDX tumour	99.3	100.00
**Unknown 19**	PDX tumour	10	2.06E+06	PDX tumour	99.27	
**Unknown 19**	PDX tumour	4	8.77E+05	PDX tumour	99.38	
**Unknown 19**	PDX tumour	5	9.87E+05	PDX tumour	99.35	
**Unknown 19**	PDX tumour	11	5.20E+06	PDX tumour	99.39	
**Unknown 19**	PDX tumour	10	5.21E+06	PDX tumour	99.37	
**Unknown 19**	PDX tumour	10	7.21E+06	PDX tumour	99.36	
**Unknown 19**	PDX tumour	9	4.87E+06	PDX tumour	99.37	
**Unknown 19**	PDX tumour	9	6.03E+06	PDX tumour	99.37	
**Unknown 19**	PDX tumour	9	6.43E+06	PDX tumour	99.38	
**Unknown 19**	PDX tumour	9	7.25E+06	PDX tumour	99.36	
**Unknown 19**	PDX tumour	11	3.54E+06	PDX tumour	99.36	
**Unknown 19**	PDX tumour	9	2.17E+06	PDX tumour	99.39	
**Unknown 19**	PDX tumour	11	4.93E+06	PDX tumour	99.33	
**Unknown 19**	PDX tumour	9	1.02E+07	PDX tumour	99.38	
**Unknown 19**	PDX tumour	10	1.07E+07	PDX tumour	99.38	
**Unknown 20**	PDX tumour	10	2.75E+06	PDX tumour	99.29	100.00
**Unknown 20**	PDX tumour	10	3.52E+06	PDX tumour	99.38	
**Unknown 20**	PDX tumour	12	5.54E+06	PDX tumour	99.33	
**Unknown 20**	PDX tumour	10	4.43E+06	PDX tumour	99.38	
**Unknown 20**	PDX tumour	9	3.23E+06	PDX tumour	99.2	
**Unknown 21**	PDX tumour	11	5.56E+06	PDX tumour	99.32	100.00
**Unknown 21**	PDX tumour	10	5.03E+06	PDX tumour	99.24	
**Unknown 21**	PDX tumour	9	3.30E+06	PDX tumour	99.23	
**Unknown 21**	PDX tumour	10	4.46E+06	PDX tumour	99.32	
**Unknown 21**	PDX tumour	11	3.97E+06	PDX tumour	99.27	
**Unknown 21**	PDX tumour	13	5.31E+06	PDX tumour	99.36	

**Unknown 21**	PDX tumour	9	3.92E+06	PDX tumour	99.35	
**Unknown 21**	PDX tumour	11	5.35E+06	PDX tumour	99.37	
**Unknown 21**	PDX tumour	10	4.39E+06	PDX tumour	99.32	
**Unknown 21**	PDX tumour	9	3.38E+06	PDX tumour	99.34	
**Unknown 21**	PDX tumour	11	4.77E+06	PDX tumour	99.38	
**Unknown 21**	PDX tumour	8	2.79E+06	PDX tumour	99.36	
**Unknown 21**	PDX tumour	11	6.09E+06	PDX tumour	99.37	
**Unknown 22**	Lymphoma	10	3.42E+06	Lymphoma	98.93	100.00
**Unknown 22**	Lymphoma	12	3.45E+06	Lymphoma	98.88	
**Unknown 22**	Lymphoma	11	4.08E+06	Lymphoma	99.25	
**Unknown 22**	Lymphoma	13	4.80E+05	Lymphoma	96.47	
**Unknown 22**	Lymphoma	13	1.68E+06	Unclassifiable	-	
**Unknown 22**	Lymphoma	8	2.39E+06	Lymphoma	98.93	
**Unknown 22**	Lymphoma	10	3.45E+06	Lymphoma	97.24	
**Unknown 22**	Lymphoma	9	3.24E+06	Lymphoma	98.59	
**Unknown 22**	Lymphoma	10	4.52E+06	Lymphoma	99.21	
**Unknown 22**	Lymphoma	10	4.47E+06	Lymphoma	99.22	
**Unknown 22**	Lymphoma	10	1.96E+06	Lymphoma	97.79	
**Unknown 22**	Lymphoma	10	1.26E+06	Lymphoma	98.6	
**Unknown 22**	Lymphoma	10	1.64E+06	Lymphoma	99.29	
**Unknown 22**	Lymphoma	11	2.09E+06	Lymphoma	99.3	
**Unknown 22**	Lymphoma	10	3.03E+06	Lymphoma	99.22	
**Unknown 23**	PDX tumour	7	8.21E+05	Unclassifiable	-	100.00
**Unknown 23**	PDX tumour	7	9.71E+05	PDX tumour	98.98	
**Unknown 23**	PDX tumour	13	8.92E+05	Unclassifiable	-	
**Unknown 23**	PDX tumour	9	1.08E+06	PDX tumour	99.32	
**Unknown 23**	PDX tumour	10	2.07E+06	Unclassifiable	-	
**Unknown 23**	PDX tumour	9	2.30E+06	Unclassifiable	-	
**Unknown 23**	PDX tumour	11	2.23E+06	Unclassifiable	-	
**Unknown 23**	PDX tumour	9	2.30E+06	Unclassifiable	-	
**Unknown 23**	PDX tumour	10	1.96E+06	Unclassifiable	-	
**Unknown 23**	PDX tumour	9	1.50E+06	Unclassifiable	-	
**Unknown 23**	PDX tumour	11	1.58E+06	PDX tumour	97.91	
**Unknown 23**	PDX tumour	7	5.93E+05	PDX tumour	98.87	
**Unknown 24**	PDX tumour	12	2.10E+06	PDX tumour	99.37	100.00
**Unknown 24**	PDX tumour	11	2.07E+06	PDX tumour	99.35	
**Unknown 24**	PDX tumour	12	2.14E+06	PDX tumour	99.37	
**Unknown 24**	PDX tumour	10	1.73E+06	PDX tumour	99.36	
**Unknown 24**	PDX tumour	14	1.22E+06	PDX tumour	99.38	
**Unknown 24**	PDX tumour	3	3.32E+05	bad data	-	
**Unknown 24**	PDX tumour	4	1.05E+06	PDX tumour	99.22	
**Unknown 24**	PDX tumour	11	2.86E+06	PDX tumour	99.37	

**Unknown 24**	PDX tumour	14	2.90E+06	PDX tumour	99.38	
**Unknown 24**	PDX tumour	13	4.69E+06	PDX tumour	99.35	
**Unknown 25**	Lymphoma	10	3.99E+06	Lymphoma	99.15	100.00
**Unknown 25**	Lymphoma	10	3.71E+06	Lymphoma	99.35	
**Unknown 25**	Lymphoma	10	2.37E+06	Lymphoma	98.86	
**Unknown 25**	Lymphoma	12	2.49E+06	Lymphoma	99.3	
**Unknown 25**	Lymphoma	9	3.38E+06	Lymphoma	98.54	
**Unknown 25**	Lymphoma	9	3.11E+06	Lymphoma	99.33	
**Unknown 25**	Lymphoma	9	3.07E+06	Lymphoma	99.24	
**Unknown 25**	Lymphoma	8	2.62E+06	Lymphoma	98.75	
**Unknown 25**	Lymphoma	11	2.96E+06	Lymphoma	99.14	
**Unknown 25**	Lymphoma	11	3.70E+06	Lymphoma	99.37	
**Unknown 25**	Lymphoma	10	2.88E+06	Lymphoma	98.94	
**Unknown 25**	Lymphoma	9	1.35E+06	Lymphoma	92.4	
**Unknown 25**	Lymphoma	14	2.85E+06	Lymphoma	99.34	
**Unknown 25**	Lymphoma	14	2.17E+06	Lymphoma	98.99	
**Unknown 25**	Lymphoma	12	1.82E+06	Lymphoma	99.3	
**Unknown 26**	PDX tumour	12	3.92E+06	PDX tumour	99.37	100.00
**Unknown 26**	PDX tumour	13	2.47E+06	PDX tumour	99.32	
**Unknown 26**	PDX tumour	11	2.76E+06	PDX tumour	99.22	
**Unknown 26**	PDX tumour	10	2.17E+06	PDX tumour	99.34	
**Unknown 26**	PDX tumour	11	1.99E+06	PDX tumour	99.38	
**Unknown 26**	PDX tumour	12	2.18E+06	PDX tumour	99.38	
**Unknown 26**	PDX tumour	11	2.28E+06	Unclassifiable	-	
**Unknown 26**	PDX tumour	10	2.47E+06	PDX tumour	99.29	
**Unknown 26**	PDX tumour	10	2.69E+06	Unclassifiable	-	
**Unknown 26**	PDX tumour	10	2.02E+06	Unclassifiable	-	
**Unknown 26**	PDX tumour	12	1.85E+06	PDX tumour	99.33	
**Unknown 26**	PDX tumour	12	2.58E+06	PDX tumour	99.26	
**Unknown 26**	PDX tumour	12	3.45E+06	PDX tumour	99.3	
**Unknown 26**	PDX tumour	6	1.93E+06	PDX tumour	99.38	
**Unknown 26**	PDX tumour	5	1.10E+06	PDX tumour	99.31	
**Unknown 26**	PDX tumour	14	1.83E+06	PDX tumour	99.32	
**Unknown 27**	PDX tumour	12	3.43E+06	PDX tumour	98.47	100.00
**Unknown 27**	PDX tumour	11	1.08E+06	PDX tumour	97.85	
**Unknown 27**	PDX tumour	10	2.76E+06	PDX tumour	99.23	
**Unknown 27**	PDX tumour	10	2.12E+06	PDX tumour	98.74	
**Unknown 27**	PDX tumour	9	1.37E+06	PDX tumour	98.76	
**Unknown 27**	PDX tumour	10	1.14E+06	PDX tumour	98.73	
**Unknown 27**	PDX tumour	7	8.27E+05	PDX tumour	99.35	
**Unknown 28**	Lymphoma	10	8.40E+06	Lymphoma	99.29	100.00
**Unknown 28**	Lymphoma	9	4.29E+06	Lymphoma	99.08	

**Unknown 28**	Lymphoma	9	3.88E+06	Lymphoma	96.94	
**Unknown 28**	Lymphoma	6	2.45E+06	Lymphoma	98.78	
**Unknown 28**	Lymphoma	6	2.36E+06	Lymphoma	74.98	
**Unknown 28**	Lymphoma	9	4.63E+06	Lymphoma	99.24	
**Unknown 29**	PDX tumour	10	1.04E+07	Lymphoma	89.73	72.73
**Unknown 29**	PDX tumour	10	9.59E+06	Lymphoma	97.76	
**Unknown 29**	PDX tumour	8	5.66E+06	PDX tumour	98.64	
**Unknown 29**	PDX tumour	10	5.24E+06	PDX tumour	99.38	
**Unknown 29**	PDX tumour	10	3.24E+06	PDX tumour	99.36	
**Unknown 29**	PDX tumour	12	2.52E+06	PDX tumour	99.36	
**Unknown 29**	PDX tumour	10	1.98E+06	PDX tumour	99.22	
**Unknown 29**	PDX tumour	10	5.43E+06	Unclassifiable	-	
**Unknown 29**	PDX tumour	8	6.43E+06	Lymphoma	76.17	
**Unknown 29**	PDX tumour	9	3.83E+06	PDX tumour	99.15	
**Unknown 29**	PDX tumour	9	5.66E+06	PDX tumour	99.12	
**Unknown 29**	PDX tumour	13	4.16E+06	PDX tumour	99.35	
**Unknown 30**	PDX tumour	13	1.91E+06	PDX tumour	99.38	100.00
**Unknown 30**	PDX tumour	10	1.34E+06	PDX tumour	99.39	
**Unknown 30**	PDX tumour	16	1.90E+06	PDX tumour	99.38	
**Unknown 30**	PDX tumour	13	1.67E+06	PDX tumour	99.37	
**Unknown 30**	PDX tumour	15	2.39E+06	PDX tumour	99.35	
**Unknown 30**	PDX tumour	13	3.87E+06	PDX tumour	99.31	
**Unknown 30**	PDX tumour	12	2.77E+06	PDX tumour	99.38	
**Unknown 30**	PDX tumour	17	7.24E+05	PDX tumour	99.38	
**Unknown 30**	PDX tumour	5	1.07E+06	PDX tumour	99.36	
**Unknown 30**	PDX tumour	10	1.49E+06	PDX tumour	99.38	
**Unknown 31**	PDX tumour	14	2.90E+06	PDX tumour	99.37	100.00
**Unknown 31**	PDX tumour	12	1.78E+06	PDX tumour	99.39	
**Unknown 31**	PDX tumour	12	2.84E+06	PDX tumour	99.38	
**Unknown 31**	PDX tumour	11	2.17E+06	PDX tumour	99.38	
**Unknown 31**	PDX tumour	11	1.96E+06	PDX tumour	99.39	
**Unknown 31**	PDX tumour	16	2.46E+06	PDX tumour	99.39	
**Unknown 31**	PDX tumour	16	1.55E+06	PDX tumour	99.38	
**Unknown 31**	PDX tumour	6	1.44E+06	PDX tumour	99.39	
**Unknown 31**	PDX tumour	7	1.63E+06	PDX tumour	99.39	
**Unknown 32**	PDX tumour	16	1.30E+06	PDX tumour	98.4	100.00
**Unknown 32**	PDX tumour	10	7.87E+05	PDX tumour	99.23	
**Unknown 32**	PDX tumour	9	8.25E+05	PDX tumour	99.35	
**Unknown 32**	PDX tumour	9	1.02E+06	PDX tumour	98.98	
**Unknown 32**	PDX tumour	15	1.41E+06	PDX tumour	99.33	
**Unknown 32**	PDX tumour	16	1.47E+06	PDX tumour	99.3	
**Unknown 32**	PDX tumour	11	1.65E+06	PDX tumour	98.96	

**Unknown 32**	PDX tumour	12	1.86E+06	PDX tumour	99.27	
**Unknown 32**	PDX tumour	14	2.02E+06	PDX tumour	99.38	
**Unknown 32**	PDX tumour	7	7.29E+05	PDX tumour	99.37	
**Unknown 32**	PDX tumour	11	1.99E+06	PDX tumour	99.34	
**Unknown 32**	PDX tumour	8	1.94E+06	PDX tumour	98.96	
**Unknown 32**	PDX tumour	9	9.58E+05	PDX tumour	98.54	
**Unknown 32**	PDX tumour	10	1.42E+06	PDX tumour	99.22	
**Unknown 32**	PDX tumour	9	7.23E+05	PDX tumour	99.2	
**Unknown 33**	PDX tumour	12	8.13E+06	PDX tumour	99.2	100.00
**Unknown 33**	PDX tumour	12	5.42E+06	PDX tumour	99.33	
**Unknown 33**	PDX tumour	13	6.55E+06	PDX tumour	99.28	
**Unknown 33**	PDX tumour	13	6.39E+06	PDX tumour	98.89	
**Unknown 33**	PDX tumour	11	2.55E+06	PDX tumour	99.36	
**Unknown 33**	PDX tumour	13	6.32E+06	PDX tumour	99.21	
**Unknown 33**	PDX tumour	13	5.76E+06	PDX tumour	99.22	
**Unknown 33**	PDX tumour	11	4.34E+06	PDX tumour	99.18	
**Unknown 33**	PDX tumour	11	5.08E+06	PDX tumour	99.26	
**Unknown 33**	PDX tumour	11	5.06E+06	PDX tumour	99.38	
**Unknown 33**	PDX tumour	11	4.44E+06	PDX tumour	99.34	
**Unknown 33**	PDX tumour	12	6.29E+06	PDX tumour	99.11	
**Unknown 33**	PDX tumour	9	5.76E+06	PDX tumour	99.13	
**Unknown 34**	PDX tumour	12	2.38E+06	PDX tumour	99.39	100.00
**Unknown 34**	PDX tumour	10	1.04E+06	PDX tumour	99.34	
**Unknown 34**	PDX tumour	12	1.56E+06	PDX tumour	99.37	
**Unknown 34**	PDX tumour	6	6.28E+05	PDX tumour	99.35	
**Unknown 34**	PDX tumour	12	1.05E+06	PDX tumour	99.38	
**Unknown 34**	PDX tumour	5	4.59E+05	PDX tumour	99.35	
**Unknown 34**	PDX tumour	8	9.65E+05	PDX tumour	99.38	
**Unknown 34**	PDX tumour	10	1.74E+06	PDX tumour	99.34	
**Unknown 34**	PDX tumour	5	4.62E+05	PDX tumour	99.24	
**Unknown 34**	PDX tumour	9	1.99E+06	PDX tumour	99.38	
**Unknown 34**	PDX tumour	13	2.58E+06	PDX tumour	99.38	
**Unknown 34**	PDX tumour	9	3.19E+06	PDX tumour	99.33	
**Unknown 34**	PDX tumour	8	3.62E+06	PDX tumour	99.38	
**Unknown 34**	PDX tumour	9	2.35E+06	PDX tumour	99.37	
**Unknown 34**	PDX tumour	12	1.93E+06	PDX tumour	99.36	
**Unknown 34**	PDX tumour	11	1.70E+06	PDX tumour	99.38	
**Unknown 34**	PDX tumour	9	2.05E+06	PDX tumour	99.36	
**Unknown 34**	PDX tumour	11	2.00E+06	PDX tumour	99.38	
**Unknown 34**	PDX tumour	10	3.18E+06	PDX tumour	99.39	
**Unknown 34**	PDX tumour	8	2.34E+06	PDX tumour	99.38	
**Unknown 34**	PDX tumour	11	2.12E+06	PDX tumour	99.31	

**Unknown 34**	PDX tumour	7	2.16E+06	PDX tumour	99.38	
**Unknown 35**	PDX tumour	9	3.20E+06	PDX tumour	99.37	100.00
**Unknown 35**	PDX tumour	9	2.28E+06	PDX tumour	99.37	
**Unknown 35**	PDX tumour	8	2.18E+06	PDX tumour	99.26	
**Unknown 35**	PDX tumour	8	2.02E+06	PDX tumour	99.27	
**Unknown 35**	PDX tumour	12	3.42E+06	PDX tumour	99.24	
**Unknown 35**	PDX tumour	7	2.86E+06	PDX tumour	99.28	
**Unknown 35**	PDX tumour	8	2.78E+06	PDX tumour	99.27	
**Unknown 35**	PDX tumour	12	3.50E+06	PDX tumour	99.28	
**Unknown 35**	PDX tumour	7	2.91E+06	PDX tumour	99.23	
**Unknown 35**	PDX tumour	7	2.75E+06	PDX tumour	99.25	
**Unknown 35**	PDX tumour	8	2.60E+06	PDX tumour	99.22	
**Unknown 35**	PDX tumour	7	1.47E+06	PDX tumour	99.36	
**Unknown 35**	PDX tumour	8	2.53E+06	PDX tumour	99.31	
**Unknown 35**	PDX tumour	8	2.55E+06	PDX tumour	99.25	
**Unknown 35**	PDX tumour	9	3.96E+06	PDX tumour	99.3	
**Unknown 35**	PDX tumour	8	4.94E+06	PDX tumour	99.3	
**Unknown 35**	PDX tumour	11	2.69E+06	PDX tumour	99.24	
**Unknown 35**	PDX tumour	9	2.51E+06	PDX tumour	99.29	
**Unknown 35**	PDX tumour	8	3.25E+06	PDX tumour	99.29	
**Unknown 35**	PDX tumour	12	3.47E+06	PDX tumour	99.35	
**Unknown 35**	PDX tumour	9	2.09E+06	PDX tumour	99.23	
**Unknown 35**	PDX tumour	9	1.73E+06	PDX tumour	99.32	
**Unknown 35**	PDX tumour	9	1.66E+06	PDX tumour	99.35	
**Unknown 35**	PDX tumour	9	3.74E+06	PDX tumour	99.29	
**Unknown 36**	PDX tumour	12	1.44E+07	Lymphoma	<u>73.97</u>	92.31
**Unknown 36**	PDX tumour	10	7.74E+06	Unclassifiable	-	
**Unknown 36**	PDX tumour	12	8.56E+06	Unclassifiable	-	
**Unknown 36**	PDX tumour	11	6.15E+06	PDX tumour	98.19	
**Unknown 36**	PDX tumour	11	7.41E+06	Unclassifiable	-	
**Unknown 36**	PDX tumour	11	8.96E+06	Unclassifiable	-	
**Unknown 36**	PDX tumour	13	7.59E+06	Unclassifiable	-	
**Unknown 36**	PDX tumour	12	7.44E+06	PDX tumour	97.92	
**Unknown 36**	PDX tumour	11	4.79E+06	PDX tumour	98.43	
**Unknown 36**	PDX tumour	13	7.12E+06	PDX tumour	98.98	
**Unknown 36**	PDX tumour	12	6.14E+06	Unclassifiable	-	
**Unknown 36**	PDX tumour	11	6.02E+06	PDX tumour	98.09	
**Unknown 36**	PDX tumour	14	5.21E+06	PDX tumour	99.21	
**Unknown 36**	PDX tumour	11	1.13E+07	Unclassifiable	-	
**Unknown 36**	PDX tumour	12	7.67E+06	PDX tumour	99.13	
**Unknown 36**	PDX tumour	13	1.24E+07	PDX tumour	99.23	
**Unknown 36**	PDX tumour	11	1.24E+07	PDX tumour	99.24	

**Unknown 36**	PDX tumour	12	1.56E+07	PDX tumour	99.15	
**Unknown 36**	PDX tumour	13	8.94E+06	PDX tumour	98.43	
**Unknown 36**	PDX tumour	13	1.96E+07	PDX tumour	99.28	
**Unknown 37**	PDX tumour	12	3.12E+06	PDX tumour	99.38	100.00
**Unknown 37**	PDX tumour	16	2.17E+06	PDX tumour	99.33	
**Unknown 37**	PDX tumour	16	2.79E+06	PDX tumour	99.32	
**Unknown 37**	PDX tumour	15	2.88E+06	PDX tumour	99.3	
**Unknown 37**	PDX tumour	13	2.07E+06	PDX tumour	99.29	
**Unknown 37**	PDX tumour	14	3.18E+06	PDX tumour	99.27	
**Unknown 37**	PDX tumour	12	3.47E+06	PDX tumour	99.38	
**Unknown 37**	PDX tumour	14	2.75E+06	PDX tumour	99.37	
**Unknown 37**	PDX tumour	14	4.37E+06	PDX tumour	99.34	
**Unknown 37**	PDX tumour	10	2.62E+06	PDX tumour	99.34	
**Unknown 38**	PDX tumour	11	3.97E+06	PDX tumour	98.82	80.00
**Unknown 38**	PDX tumour	10	2.34E+06	PDX tumour	98.22	
**Unknown 38**	PDX tumour	11	4.59E+06	PDX tumour	98.92	
**Unknown 38**	PDX tumour	10	3.10E+06	PDX tumour	99.2	
**Unknown 38**	PDX tumour	12	6.44E+06	PDX tumour	98.44	
**Unknown 38**	PDX tumour	9	3.51E+06	PDX tumour	99.19	
**Unknown 38**	PDX tumour	9	2.09E+06	Lymphoma	92.92	
**Unknown 38**	PDX tumour	9	1.87E+06	PDX tumour	97.91	
**Unknown 38**	PDX tumour	9	4.21E+06	Unclassifiable	-	
**Unknown 38**	PDX tumour	9	2.76E+06	PDX tumour	99.3	
**Unknown 38**	PDX tumour	11	4.53E+06	Lymphoma	94.98	
**Unknown 38**	PDX tumour	9	5.32E+06	Unclassifiable	-	
**Unknown 39**	PDX tumour	13	1.31E+06	PDX tumour	99.28	100.00
**Unknown 39**	PDX tumour	11	1.78E+06	PDX tumour	99.36	
**Unknown 39**	PDX tumour	12	2.18E+06	PDX tumour	99.21	
**Unknown 39**	PDX tumour	8	1.52E+06	PDX tumour	99.35	
**Unknown 39**	PDX tumour	17	4.02E+06	PDX tumour	99.38	
**Unknown 39**	PDX tumour	10	3.90E+06	PDX tumour	99.33	
**Unknown 39**	PDX tumour	11	4.57E+06	PDX tumour	99.3	
**Unknown 39**	PDX tumour	17	3.66E+06	PDX tumour	99.38	
**Unknown 39**	PDX tumour	9	1.86E+06	PDX tumour	99.38	
**Unknown 40**	PDX tumour	14	4.10E+06	PDX tumour	99.31	100.00
**Unknown 40**	PDX tumour	8	1.30E+06	PDX tumour	99.35	
**Unknown 40**	PDX tumour	7	9.58E+05	PDX tumour	99.37	
**Unknown 40**	PDX tumour	12	1.61E+06	PDX tumour	99.38	
**Unknown 40**	PDX tumour	6	6.07E+05	PDX tumour	99.38	
**Unknown 40**	PDX tumour	15	7.15E+05	PDX tumour	99.36	
**Unknown 40**	PDX tumour	10	1.12E+06	PDX tumour	99.39	
**Unknown 40**	PDX tumour	9	8.88E+05	PDX tumour	99.36	

**Unknown 40**	PDX tumour	9	1.65E+06	PDX tumour	99.35	
**Unknown 40**	PDX tumour	11	2.02E+06	PDX tumour	99.38	
**Unknown 40**	PDX tumour	9	1.77E+06	PDX tumour	99.36	
**Unknown 40**	PDX tumour	6	8.64E+05	PDX tumour	99.36	
**Unknown 40**	PDX tumour	12	1.03E+06	PDX tumour	99.38	
**Unknown 40**	PDX tumour	15	9.21E+05	PDX tumour	99.37	
**Unknown 40**	PDX tumour	2	5.00E+05	bad data	-	
**Unknown 40**	PDX tumour	13	5.75E+06	PDX tumour	99.27	
**Unknown 40**	PDX tumour	10	4.27E+06	PDX tumour	99.39	
**Unknown 41**	PDX tumour	10	4.13E+06	PDX tumour	99.3	100.00
**Unknown 41**	PDX tumour	8	6.30E+06	PDX tumour	99.39	
**Unknown 41**	PDX tumour	8	6.27E+06	PDX tumour	99.39	
**Unknown 41**	PDX tumour	9	6.84E+06	PDX tumour	99.37	
**Unknown 42**	PDX tumour	13	3.19E+06	PDX tumour	98.75	100.00
**Unknown 42**	PDX tumour	13	1.11E+06	PDX tumour	99.12	
**Unknown 42**	PDX tumour	9	1.34E+06	PDX tumour	99.38	
**Unknown 42**	PDX tumour	8	1.15E+06	PDX tumour	99.38	
**Unknown 42**	PDX tumour	9	9.98E+05	PDX tumour	99.34	
**Unknown 42**	PDX tumour	8	1.06E+06	PDX tumour	99.36	
**Unknown 42**	PDX tumour	7	6.77E+05	PDX tumour	99.32	
**Unknown 42**	PDX tumour	11	2.47E+06	PDX tumour	99.23	
**Unknown 42**	PDX tumour	12	2.14E+06	PDX tumour	99.38	
**Unknown 42**	PDX tumour	11	2.21E+06	PDX tumour	99.34	
**Unknown 42**	PDX tumour	12	2.68E+06	PDX tumour	99.38	
**Unknown 42**	PDX tumour	8	1.64E+06	PDX tumour	99.37	
**Unknown 42**	PDX tumour	12	1.47E+06	PDX tumour	99.24	
**Unknown 42**	PDX tumour	11	4.31E+06	PDX tumour	99.28	
**Unknown 42**	PDX tumour	11	2.06E+06	PDX tumour	98.86	
**Unknown 42**	PDX tumour	9	1.46E+06	PDX tumour	99.14	
**Unknown 43**	PDX tumour	11	2.89E+06	PDX tumour	99.35	100.00
**Unknown 43**	PDX tumour	12	3.02E+06	PDX tumour	99.38	
**Unknown 43**	PDX tumour	12	2.17E+06	PDX tumour	99.36	
**Unknown 43**	PDX tumour	11	2.34E+06	PDX tumour	99.37	
**Unknown 43**	PDX tumour	12	1.19E+06	PDX tumour	99.37	
**Unknown 43**	PDX tumour	11	3.98E+06	PDX tumour	99.38	
**Unknown 43**	PDX tumour	9	2.70E+06	PDX tumour	99.38	
**Unknown 43**	PDX tumour	11	2.56E+06	PDX tumour	99.38	
**Unknown 43**	PDX tumour	12	3.75E+06	PDX tumour	99.39	
**Unknown 43**	PDX tumour	15	2.29E+06	PDX tumour	99.26	
**Unknown 44**	PDX tumour	12	4.89E+06	PDX tumour	99.29	100.00
**Unknown 44**	PDX tumour	13	3.15E+06	PDX tumour	99.35	
**Unknown 44**	PDX tumour	13	2.59E+06	PDX tumour	99.37	

**Unknown 44**	PDX tumour	13	2.75E+06	PDX tumour	99.38	
**Unknown 44**	PDX tumour	13	6.28E+06	PDX tumour	99.33	
**Unknown 44**	PDX tumour	13	5.87E+06	PDX tumour	99.31	
**Unknown 44**	PDX tumour	12	7.22E+06	PDX tumour	99.33	
**Unknown 44**	PDX tumour	13	8.25E+06	PDX tumour	99.38	
**Unknown 44**	PDX tumour	11	6.65E+06	PDX tumour	99.38	
**Unknown 44**	PDX tumour	12	5.40E+06	PDX tumour	99.33	
**Unknown 44**	PDX tumour	10	6.05E+06	PDX tumour	99.39	
**Unknown 45**	Lymphoma	12	1.13E+07	Lymphoma	99.29	92.86
**Unknown 45**	Lymphoma	12	1.15E+07	Lymphoma	99.38	
**Unknown 45**	Lymphoma	11	7.11E+06	Lymphoma	98.73	
**Unknown 45**	Lymphoma	11	6.07E+06	PDX tumour	98.8	
**Unknown 45**	Lymphoma	12	5.58E+06	Unclassifiable	-	
**Unknown 45**	Lymphoma	12	2.15E+06	Unclassifiable	-	
**Unknown 45**	Lymphoma	12	1.58E+06	Lymphoma	98.75	
**Unknown 45**	Lymphoma	8	1.74E+06	Lymphoma	98.49	
**Unknown 45**	Lymphoma	11	4.44E+06	Lymphoma	99.38	
**Unknown 45**	Lymphoma	12	8.94E+06	Lymphoma	99.39	
**Unknown 45**	Lymphoma	11	1.37E+07	Lymphoma	99.36	
**Unknown 45**	Lymphoma	11	1.17E+07	Lymphoma	99.38	
**Unknown 45**	Lymphoma	11	1.44E+07	Lymphoma	99.37	
**Unknown 45**	Lymphoma	10	1.56E+07	Lymphoma	99.29	
**Unknown 45**	Lymphoma	10	8.77E+06	Lymphoma	99.39	
**Unknown 45**	Lymphoma	10	7.07E+06	Lymphoma	99.26	
**Unknown 46**	PDX tumour	13	2.68E+06	PDX tumour	99.38	100.00
**Unknown 46**	PDX tumour	10	2.53E+06	PDX tumour	99.39	
**Unknown 46**	PDX tumour	11	1.13E+06	PDX tumour	99.15	
**Unknown 46**	PDX tumour	5	4.03E+05	PDX tumour	99.39	
**Unknown 46**	PDX tumour	11	1.77E+06	PDX tumour	99.34	
**Unknown 46**	PDX tumour	11	1.72E+06	PDX tumour	99.35	
**Unknown 46**	PDX tumour	11	2.40E+06	PDX tumour	99.36	
**Unknown 46**	PDX tumour	12	3.70E+06	PDX tumour	99.3	
**Unknown 46**	PDX tumour	13	2.88E+06	PDX tumour	99.3	
**Unknown 46**	PDX tumour	9	2.40E+06	PDX tumour	99.17	
**Unknown 46**	PDX tumour	10	2.53E+06	PDX tumour	99.38	
**Unknown 46**	PDX tumour	10	3.67E+06	PDX tumour	99.32	
**Unknown 46**	PDX tumour	9	2.02E+06	PDX tumour	99.37	
**Unknown 46**	PDX tumour	10	4.31E+06	PDX tumour	99.38	
**Unknown 46**	PDX tumour	7	1.10E+06	PDX tumour	99.21	
**Unknown 47**	Lymphoma	11	4.18E+06	Lymphoma	99.33	100.00
**Unknown 47**	Lymphoma	10	3.09E+06	Lymphoma	98.87	
**Unknown 47**	Lymphoma	12	2.63E+06	Lymphoma	99.09	

**Unknown 47**	Lymphoma	14	3.02E+06	Lymphoma	98.99	
**Unknown 47**	Lymphoma	11	3.18E+06	Lymphoma	99.11	
**Unknown 47**	Lymphoma	13	6.52E+06	Lymphoma	98.65	
**Unknown 47**	Lymphoma	10	1.52E+06	Lymphoma	99.26	
**Unknown 47**	Lymphoma	10	3.84E+06	Lymphoma	96.46	
**Unknown 47**	Lymphoma	11	6.36E+06	Lymphoma	98.43	
**Unknown 47**	Lymphoma	14	6.50E+06	Lymphoma	97.94	
**Unknown 47**	Lymphoma	6	1.45E+06	Lymphoma	96.68	
**Unknown 47**	Lymphoma	11	2.77E+06	Lymphoma	99.29	
**Unknown 47**	Lymphoma	10	2.14E+06	Lymphoma	98.95	
**Unknown 47**	Lymphoma	11	3.26E+05	Lymphoma	99.18	
**Unknown 47**	Lymphoma	9	2.41E+06	Lymphoma	98.58	
**Unknown 47**	Lymphoma	4	4.52E+05	Lymphoma	99.05	
**Unknown 48**	Lymphoma	13	1.08E+07	Lymphoma	99.24	100.00
**Unknown 48**	Lymphoma	14	5.37E+06	Lymphoma	99.38	
**Unknown 48**	Lymphoma	11	6.74E+06	Lymphoma	99.39	
**Unknown 48**	Lymphoma	14	5.81E+06	Lymphoma	99.37	
**Unknown 48**	Lymphoma	13	6.77E+06	Lymphoma	99.38	
**Unknown 48**	Lymphoma	12	7.77E+06	Lymphoma	99.38	
**Unknown 48**	Lymphoma	11	7.31E+06	Lymphoma	99.38	
**Unknown 48**	Lymphoma	11	7.58E+06	Lymphoma	99.35	
**Unknown 48**	Lymphoma	10	9.44E+06	Lymphoma	99.25	
**Unknown 48**	Lymphoma	11	5.70E+06	Lymphoma	99.24	
**Unknown 48**	Lymphoma	14	5.47E+06	Lymphoma	99.38	
**Unknown 48**	Lymphoma	9	4.88E+06	Lymphoma	99.24	
**Unknown 49**	Lymphoma	12	1.48E+07	Lymphoma	99.38	100.00
**Unknown 49**	Lymphoma	11	1.53E+07	Lymphoma	99.36	
**Unknown 49**	Lymphoma	11	1.08E+07	Lymphoma	99.39	
**Unknown 49**	Lymphoma	11	8.03E+06	Lymphoma	99.38	
**Unknown 49**	Lymphoma	11	8.97E+06	Lymphoma	99.38	
**Unknown 49**	Lymphoma	11	1.09E+07	Lymphoma	99.39	
**Unknown 49**	Lymphoma	14	1.39E+07	Lymphoma	99.36	
**Unknown 49**	Lymphoma	10	1.08E+07	Lymphoma	99.35	
**Unknown 49**	Lymphoma	11	1.51E+07	Lymphoma	99.26	
**Unknown 49**	Lymphoma	11	1.93E+07	Lymphoma	99.29	
**Unknown 49**	Lymphoma	11	1.78E+07	Lymphoma	99.38	
**Unknown 49**	Lymphoma	11	1.36E+07	Lymphoma	99.37	
**Unknown 49**	Lymphoma	10	1.22E+07	Lymphoma	99.36	
**Unknown 49**	Lymphoma	10	1.07E+07	Lymphoma	99.32	
**Unknown 49**	Lymphoma	11	1.59E+07	Lymphoma	99.32	
**Unknown 49**	Lymphoma	10	1.14E+07	Lymphoma	99.34	
**Unknown 49**	Lymphoma	11	1.75E+07	Lymphoma	99.37	

**Unknown 49**	Lymphoma	11	1.87E+07	Lymphoma	99.39	
**Unknown 49**	Lymphoma	10	2.49E+07	Lymphoma	99.27	
**Unknown 50**	PDX tumour	9	1.95E+06	PDX tumour	99.35	100.00
**Unknown 50**	PDX tumour	10	2.16E+06	PDX tumour	99.37	
**Unknown 50**	PDX tumour	8	1.12E+06	PDX tumour	99.39	
**Unknown 50**	PDX tumour	8	1.57E+06	PDX tumour	99.36	
**Unknown 50**	PDX tumour	8	1.92E+06	PDX tumour	99.36	
**Unknown 50**	PDX tumour	9	1.58E+06	PDX tumour	99.38	
**Unknown 50**	PDX tumour	12	1.84E+06	PDX tumour	99.38	
**Unknown 50**	PDX tumour	9	1.23E+06	PDX tumour	99.38	
**Unknown 50**	PDX tumour	9	1.78E+06	PDX tumour	99.39	
**Unknown 50**	PDX tumour	12	1.79E+06	PDX tumour	99.27	
**Unknown 50**	PDX tumour	9	4.55E+06	PDX tumour	99.32	
**Unknown 50**	PDX tumour	10	2.23E+06	PDX tumour	99.32	
**Unknown 50**	PDX tumour	10	3.30E+06	PDX tumour	99.32	
**Unknown 50**	PDX tumour	8	2.58E+06	PDX tumour	99.38	
**Unknown 50**	PDX tumour	9	3.91E+06	PDX tumour	99.17	
**Unknown 50**	PDX tumour	9	2.75E+06	PDX tumour	99.34	
**Unknown 50**	PDX tumour	8	3.51E+06	PDX tumour	99.36	
**Unknown 50**	PDX tumour	8	3.51E+06	PDX tumour	99.35	
**Unknown 50**	PDX tumour	10	2.70E+06	PDX tumour	99.39	
**Unknown 50**	PDX tumour	10	2.85E+06	PDX tumour	99.36	
**Unknown 50**	PDX tumour	8	1.77E+06	PDX tumour	99.34	
**Unknown 50**	PDX tumour	8	9.86E+05	PDX tumour	99.39	
**Unknown 50**	PDX tumour	8	2.54E+06	PDX tumour	99.37	
**Unknown 50**	PDX tumour	11	3.31E+06	PDX tumour	99.37	
**Unknown 51**	PDX tumour	9	3.15E+06	Unclassifiable	-	94.12
**Unknown 51**	PDX tumour	10	2.82E+06	PDX tumour	99.35	
**Unknown 51**	PDX tumour	8	1.44E+06	PDX tumour	99.38	
**Unknown 51**	PDX tumour	9	1.94E+06	PDX tumour	99.22	
**Unknown 51**	PDX tumour	6	1.32E+06	PDX tumour	99.36	
**Unknown 51**	PDX tumour	6	1.05E+06	PDX tumour	99.22	
**Unknown 51**	PDX tumour	8	2.96E+06	PDX tumour	99.14	
**Unknown 51**	PDX tumour	9	2.23E+06	PDX tumour	97.84	
**Unknown 51**	PDX tumour	9	1.40E+06	PDX tumour	99.31	
**Unknown 51**	PDX tumour	7	1.53E+06	PDX tumour	99.05	
**Unknown 51**	PDX tumour	8	2.66E+06	PDX tumour	99.28	
**Unknown 51**	PDX tumour	9	2.48E+06	PDX tumour	98.99	
**Unknown 51**	PDX tumour	9	2.63E+06	PDX tumour	99.21	
**Unknown 51**	PDX tumour	10	2.83E+06	PDX tumour	99.18	
**Unknown 51**	PDX tumour	9	3.02E+06	Unclassifiable	-	
**Unknown 51**	PDX tumour	9	3.25E+06	PDX tumour	97.58	

**Unknown 51**	PDX tumour	12	4.52E+05	PDX tumour	98.87	
**Unknown 51**	PDX tumour	5	5.10E+05	PDX tumour	99.33	
**Unknown 51**	PDX tumour	5	6.42E+05	Lymphoma	63.79	
**Unknown 52**	PDX tumour	12	1.66E+06	PDX tumour	99.39	100.00
**Unknown 52**	PDX tumour	11	1.87E+06	PDX tumour	99.38	
**Unknown 52**	PDX tumour	12	2.21E+06	PDX tumour	99.38	
**Unknown 52**	PDX tumour	13	2.37E+06	PDX tumour	99.38	
**Unknown 52**	PDX tumour	11	2.35E+06	PDX tumour	99.31	
**Unknown 52**	PDX tumour	13	3.04E+06	PDX tumour	99.37	
**Unknown 53**	PDX tumour	9	5.18E+06	PDX tumour	99.24	100.00
**Unknown 53**	PDX tumour	10	8.27E+06	PDX tumour	99.19	
**Unknown 53**	PDX tumour	10	6.53E+06	PDX tumour	99.19	
**Unknown 53**	PDX tumour	9	3.69E+06	PDX tumour	99.22	
**Unknown 53**	PDX tumour	9	6.75E+06	Unclassifiable	-	
**Unknown 53**	PDX tumour	10	4.68E+06	PDX tumour	99.19	
**Unknown 54**	PDX tumour	13	1.47E+06	PDX tumour	99.38	100.00
**Unknown 54**	PDX tumour	8	1.39E+06	PDX tumour	99.31	
**Unknown 54**	PDX tumour	8	7.77E+05	PDX tumour	99.24	
**Unknown 54**	PDX tumour	12	1.07E+06	PDX tumour	99.3	
**Unknown 54**	PDX tumour	9	1.23E+06	PDX tumour	99.39	
**Unknown 54**	PDX tumour	10	1.98E+06	PDX tumour	99.21	
**Unknown 54**	PDX tumour	10	1.37E+06	PDX tumour	99.39	
**Unknown 54**	PDX tumour	12	1.20E+06	PDX tumour	99.29	
**Unknown 54**	PDX tumour	4	1.06E+06	PDX tumour	99.33	
**Unknown 54**	PDX tumour	12	1.14E+06	PDX tumour	99.34	
**Unknown 54**	PDX tumour	8	9.14E+05	PDX tumour	99.37	
**Unknown 54**	PDX tumour	10	1.84E+06	PDX tumour	99.39	
**Unknown 54**	PDX tumour	15	1.33E+06	PDX tumour	99.39	
**Unknown 54**	PDX tumour	13	8.24E+05	PDX tumour	99.27	
**Unknown 54**	PDX tumour	10	1.62E+06	PDX tumour	99.36	
**Unknown 55**	PDX tumour	14	3.06E+06	PDX tumour	99.29	100.00
**Unknown 55**	PDX tumour	13	4.77E+06	PDX tumour	99.38	
**Unknown 55**	PDX tumour	17	2.57E+06	PDX tumour	99.38	
**Unknown 55**	PDX tumour	14	2.54E+06	PDX tumour	99.33	
**Unknown 55**	PDX tumour	13	2.62E+06	PDX tumour	99.27	
**Unknown 55**	PDX tumour	16	2.37E+06	PDX tumour	99.3	
**Unknown 55**	PDX tumour	13	2.38E+06	PDX tumour	99.25	
**Unknown 55**	PDX tumour	15	3.06E+06	PDX tumour	99.32	
**Unknown 55**	PDX tumour	17	5.81E+06	PDX tumour	99.24	
**Unknown 55**	PDX tumour	17	4.41E+06	PDX tumour	99.32	
**Unknown 55**	PDX tumour	9	2.34E+06	PDX tumour	99.34	
**Unknown 55**	PDX tumour	8	1.10E+06	PDX tumour	99.35	

**Unknown 55**	PDX tumour	14	3.57E+06	PDX tumour	99.31	
**Unknown 56**	PDX tumour	12	3.42E+06	PDX tumour	99.38	100.00
**Unknown 56**	PDX tumour	13	2.58E+06	PDX tumour	99.34	
**Unknown 56**	PDX tumour	14	2.32E+06	PDX tumour	99.36	
**Unknown 56**	PDX tumour	14	1.68E+06	PDX tumour	99.37	
**Unknown 56**	PDX tumour	15	2.55E+06	PDX tumour	99.39	
**Unknown 56**	PDX tumour	14	3.17E+06	PDX tumour	99.37	
**Unknown 56**	PDX tumour	12	2.61E+06	PDX tumour	99.38	
**Unknown 56**	PDX tumour	12	2.49E+06	PDX tumour	99.38	
**Unknown 56**	PDX tumour	16	3.12E+06	PDX tumour	99.37	
**Unknown 56**	PDX tumour	14	1.67E+06	PDX tumour	99.25	
**Unknown 56**	PDX tumour	14	2.08E+06	PDX tumour	99.39	
**Unknown 56**	PDX tumour	12	2.41E+06	PDX tumour	99.37	
**Unknown 57**	PDX tumour	10	2.21E+06	PDX tumour	99.38	100.00
**Unknown 57**	PDX tumour	8	1.78E+06	PDX tumour	99.3	
**Unknown 57**	PDX tumour	7	1.06E+06	PDX tumour	99.39	
**Unknown 58**	PDX tumour	12	3.42E+06	PDX tumour	99.38	100.00
**Unknown 58**	PDX tumour	1	1.14E+05	bad data	-	
**Unknown 58**	PDX tumour	10	5.73E+06	PDX tumour	99.37	
**Unknown 58**	PDX tumour	9	4.62E+06	PDX tumour	99.33	
**Unknown 58**	PDX tumour	10	3.54E+06	PDX tumour	99.38	
**Unknown 58**	PDX tumour	8	4.91E+06	PDX tumour	99.37	
**Unknown 58**	PDX tumour	10	2.00E+06	PDX tumour	99.15	
**Unknown 58**	PDX tumour	9	1.67E+06	PDX tumour	99.38	
**Unknown 58**	PDX tumour	7	5.80E+05	Unclassifiable	-	
**Unknown 58**	PDX tumour	3	1.06E+06	bad data	-	
**Unknown 58**	PDX tumour	9	2.69E+06	PDX tumour	99.35	
**Unknown 58**	PDX tumour	10	4.95E+06	PDX tumour	99.38	
**Unknown 58**	PDX tumour	6	1.53E+06	PDX tumour	99.35	
**Unknown 58**	PDX tumour	9	1.82E+06	PDX tumour	99.33	
**Unknown 58**	PDX tumour	9	4.54E+06	PDX tumour	99.3	
**Unknown 58**	PDX tumour	9	1.74E+06	PDX tumour	99.28	
**Unknown 58**	PDX tumour	11	5.83E+06	PDX tumour	99.38	
**Unknown 58**	PDX tumour	10	7.75E+06	PDX tumour	99.35	
**Unknown 59**	PDX tumour	16	3.91E+06	PDX tumour	99.34	100.00
**Unknown 59**	PDX tumour	13	3.71E+06	PDX tumour	99.35	
**Unknown 59**	PDX tumour	15	3.13E+06	PDX tumour	99.38	
**Unknown 59**	PDX tumour	16	3.14E+06	PDX tumour	99.38	
**Unknown 59**	PDX tumour	13	1.86E+06	PDX tumour	99.39	
**Unknown 59**	PDX tumour	14	2.44E+06	PDX tumour	99.39	
**Unknown 59**	PDX tumour	12	2.41E+06	PDX tumour	99.37	
**Unknown 59**	PDX tumour	10	6.29E+05	PDX tumour	99.39	

**Unknown 59**	PDX tumour	7	6.08E+05	PDX tumour	99.07	
**Unknown 60**	PDX tumour	12	3.32E+06	PDX tumour	98.71	100.00
**Unknown 60**	PDX tumour	11	1.57E+06	PDX tumour	98.43	
**Unknown 60**	PDX tumour	10	1.17E+06	PDX tumour	99.25	
**Unknown 60**	PDX tumour	11	1.02E+06	PDX tumour	99.3	
**Unknown 60**	PDX tumour	9	1.19E+06	PDX tumour	98.68	
**Unknown 60**	PDX tumour	9	2.51E+06	PDX tumour	99.03	
**Unknown 60**	PDX tumour	13	2.79E+06	PDX tumour	99.24	
**Unknown 60**	PDX tumour	13	1.80E+06	PDX tumour	98.56	
**Unknown 60**	PDX tumour	10	2.58E+06	PDX tumour	98.74	
**Unknown 60**	PDX tumour	10	2.20E+06	PDX tumour	99.24	
**Unknown 60**	PDX tumour	11	2.78E+06	PDX tumour	98.72	
**Unknown 60**	PDX tumour	10	2.52E+06	PDX tumour	98.51	
**Unknown 60**	PDX tumour	11	2.83E+06	PDX tumour	98.02	
**Unknown 60**	PDX tumour	12	2.33E+06	PDX tumour	99.2	
**Unknown 60**	PDX tumour	13	1.02E+07	Unclassifiable	-	
**Unknown 60**	PDX tumour	11	1.29E+07	Unclassifiable	-	
**Unknown 61**	Lymphoma	10	9.31E+06	Lymphoma	99.33	100.00
**Unknown 61**	Lymphoma	11	9.75E+06	Lymphoma	99.34	
**Unknown 61**	Lymphoma	12	1.22E+07	Lymphoma	99.37	
**Unknown 61**	Lymphoma	11	1.33E+07	Lymphoma	99.31	
**Unknown 61**	Lymphoma	11	1.60E+07	Lymphoma	99.34	
**Unknown 61**	Lymphoma	10	6.49E+06	Lymphoma	99.34	
**Unknown 61**	Lymphoma	12	3.61E+06	Lymphoma	99.05	
**Unknown 61**	Lymphoma	11	4.20E+06	Lymphoma	99.39	
**Unknown 61**	Lymphoma	10	1.11E+07	Lymphoma	99.25	
**Unknown 61**	Lymphoma	9	1.04E+07	Lymphoma	99.29	
**Unknown 61**	Lymphoma	10	1.26E+07	Lymphoma	99.31	
**Unknown 61**	Lymphoma	6	1.73E+06	Lymphoma	99.27	
**Unknown 61**	Lymphoma	10	7.75E+06	Lymphoma	99.38	
**Unknown 61**	Lymphoma	9	1.03E+07	Lymphoma	99.27	
**Unknown 61**	Lymphoma	9	7.98E+06	Lymphoma	99.3	
**Unknown 61**	Lymphoma	13	1.07E+07	Lymphoma	99.32	
**Unknown 61**	Lymphoma	10	1.06E+07	Lymphoma	99.34	
**Unknown 61**	Lymphoma	10	8.67E+06	Lymphoma	99.32	
**Unknown 61**	Lymphoma	9	8.78E+06	Lymphoma	99.36	
**Unknown 61**	Lymphoma	9	9.50E+06	Lymphoma	99.37	
**Unknown 61**	Lymphoma	11	5.96E+06	Lymphoma	99.38	
**Unknown 62**	PDX tumour	11	2.73E+06	PDX tumour	99.39	100.00
**Unknown 62**	PDX tumour	11	2.05E+06	PDX tumour	99.37	
**Unknown 62**	PDX tumour	3	3.94E+05	bad data	-	
**Unknown 62**	PDX tumour	11	2.19E+06	PDX tumour	99.37	

**Unknown 62**	PDX tumour	10	2.27E+06	PDX tumour	99.39	
**Unknown 62**	PDX tumour	10	2.46E+06	PDX tumour	99.35	
**Unknown 62**	PDX tumour	9	1.28E+06	PDX tumour	99.36	
**Unknown 62**	PDX tumour	14	1.48E+06	PDX tumour	99.38	
**Unknown 62**	PDX tumour	8	1.97E+06	PDX tumour	99.32	
**Unknown 63**	PDX tumour	11	3.33E+06	PDX tumour	97.59	100.00
**Unknown 63**	PDX tumour	10	1.84E+06	PDX tumour	99.28	
**Unknown 63**	PDX tumour	11	2.66E+06	PDX tumour	99.38	
**Unknown 63**	PDX tumour	12	3.06E+06	PDX tumour	98.96	
**Unknown 63**	PDX tumour	6	7.67E+05	Unclassifiable	-	
**Unknown 63**	PDX tumour	10	2.30E+06	PDX tumour	98.86	
**Unknown 63**	PDX tumour	11	2.76E+06	PDX tumour	98.92	
**Unknown 63**	PDX tumour	11	1.59E+06	PDX tumour	99.28	
**Unknown 63**	PDX tumour	12	2.39E+06	PDX tumour	99.21	
**Unknown 63**	PDX tumour	11	2.49E+06	Unclassifiable	-	
**Unknown 63**	PDX tumour	9	2.54E+06	PDX tumour	99.03	
**Unknown 63**	PDX tumour	6	8.21E+05	PDX tumour	99.12	
**Unknown 63**	PDX tumour	12	8.80E+06	PDX tumour	99.13	
**Unknown 63**	PDX tumour	11	4.54E+06	PDX tumour	98.72	
**Unknown 63**	PDX tumour	11	6.85E+06	PDX tumour	99.01	
**Unknown 63**	PDX tumour	12	9.00E+06	PDX tumour	98.82	
**Unknown 63**	PDX tumour	11	1.54E+07	PDX tumour	99.32	
**Unknown 63**	PDX tumour	14	8.08E+06	PDX tumour	99.26	
**Unknown 63**	PDX tumour	12	9.25E+06	PDX tumour	99.36	
**Unknown 63**	PDX tumour	11	6.48E+06	PDX tumour	99.19	
**Unknown 63**	PDX tumour	12	7.18E+06	PDX tumour	97.74	
**Unknown 63**	PDX tumour	11	1.06E+07	PDX tumour	99.22	
**Unknown 63**	PDX tumour	11	8.72E+06	PDX tumour	99.35	
**Unknown 64**	Lymphoma	11	4.47E+06	Lymphoma	99.36	100.00
**Unknown 64**	Lymphoma	11	4.67E+06	Lymphoma	99.33	
**Unknown 64**	Lymphoma	8	3.84E+06	Lymphoma	99.35	
**Unknown 64**	Lymphoma	9	5.51E+06	Lymphoma	99.35	
**Unknown 64**	Lymphoma	8	5.33E+06	Lymphoma	99.35	
**Unknown 64**	Lymphoma	10	7.51E+06	Lymphoma	99.23	
**Unknown 64**	Lymphoma	8	5.42E+06	Lymphoma	99.36	
**Unknown 64**	Lymphoma	8	4.47E+06	Lymphoma	99.34	
**Unknown 64**	Lymphoma	8	4.86E+06	Lymphoma	99.34	
**Unknown 64**	Lymphoma	2	2.32E+06	bad data	-	
**Unknown 64**	Lymphoma	7	3.04E+06	Lymphoma	99.39	
**Unknown 64**	Lymphoma	6	3.64E+06	Lymphoma	99.37	
**Unknown 64**	Lymphoma	8	5.56E+06	Lymphoma	99.35	
**Unknown 65**	PDX tumour	13	1.39E+06	PDX tumour	99.36	100.00

**Unknown 65**	PDX tumour	6	7.31E+05	PDX tumour	99.38	
**Unknown 65**	PDX tumour	1	1.13E+05	bad data	-	
**Unknown 65**	PDX tumour	9	9.06E+05	PDX tumour	99.36	
**Unknown 65**	PDX tumour	12	2.40E+06	PDX tumour	99.3	
**Unknown 65**	PDX tumour	11	1.79E+06	PDX tumour	99.33	
**Unknown 65**	PDX tumour	11	3.07E+06	PDX tumour	99.28	
**Unknown 65**	PDX tumour	13	3.52E+06	PDX tumour	99.35	
**Unknown 65**	PDX tumour	10	2.66E+06	PDX tumour	99.39	
**Unknown 65**	PDX tumour	10	2.07E+06	PDX tumour	99.26	
**Unknown 65**	PDX tumour	10	3.01E+06	PDX tumour	99.34	
**Unknown 65**	PDX tumour	12	2.51E+06	PDX tumour	99.34	
**Unknown 65**	PDX tumour	14	4.72E+06	PDX tumour	99.33	
**Unknown 66**	PDX tumour	11	5.36E+06	PDX tumour	99.31	100.00
**Unknown 66**	PDX tumour	10	4.04E+06	PDX tumour	99.28	
**Unknown 66**	PDX tumour	13	5.32E+06	PDX tumour	99.29	
**Unknown 66**	PDX tumour	10	3.97E+06	PDX tumour	99.34	
**Unknown 66**	PDX tumour	9	3.23E+06	PDX tumour	99.31	
**Unknown 66**	PDX tumour	10	1.89E+06	PDX tumour	99.38	
**Unknown 66**	PDX tumour	10	1.74E+06	PDX tumour	99.38	
**Unknown 66**	PDX tumour	11	2.76E+06	PDX tumour	99.38	
**Unknown 66**	PDX tumour	9	1.36E+06	PDX tumour	99.39	
**Unknown 66**	PDX tumour	11	1.34E+06	PDX tumour	99.38	
**Unknown 67**	PDX tumour	14	2.56E+06	PDX tumour	99.39	100.00
**Unknown 67**	PDX tumour	14	2.43E+06	PDX tumour	99.35	
**Unknown 67**	PDX tumour	13	2.05E+06	PDX tumour	99.38	
**Unknown 67**	PDX tumour	13	1.28E+06	PDX tumour	99.35	
**Unknown 67**	PDX tumour	15	1.93E+06	PDX tumour	99.25	
**Unknown 67**	PDX tumour	13	1.51E+06	PDX tumour	99.38	
**Unknown 67**	PDX tumour	12	2.20E+06	PDX tumour	99.38	
**Unknown 67**	PDX tumour	13	3.02E+06	PDX tumour	99.39	
**Unknown 67**	PDX tumour	14	1.40E+06	PDX tumour	99.36	
**Unknown 67**	PDX tumour	11	2.37E+06	PDX tumour	99.37	
**Unknown 67**	PDX tumour	7	8.72E+05	PDX tumour	99.33	
**Unknown 68**	PDX tumour	10	1.18E+07	PDX tumour	99.36	100.00
**Unknown 68**	PDX tumour	10	9.96E+06	PDX tumour	99.25	
**Unknown 68**	PDX tumour	10	1.19E+07	PDX tumour	99.39	
**Unknown 68**	PDX tumour	11	1.25E+07	PDX tumour	99.37	
**Unknown 68**	PDX tumour	11	8.87E+06	PDX tumour	99.38	
**Unknown 68**	PDX tumour	11	8.18E+06	PDX tumour	99.38	
**Unknown 68**	PDX tumour	10	9.26E+06	PDX tumour	99.39	
**Unknown 68**	PDX tumour	11	6.61E+06	PDX tumour	99.37	
**Unknown 68**	PDX tumour	11	8.74E+06	PDX tumour	99.38	

**Unknown 68**	PDX tumour	11	8.84E+06	PDX tumour	99.37	
**Unknown 68**	PDX tumour	5	4.41E+06	PDX tumour	99.39	
**Unknown 69**	PDX tumour	12	2.54E+06	PDX tumour	99.38	100.00
**Unknown 69**	PDX tumour	11	2.06E+06	PDX tumour	99.37	
**Unknown 69**	PDX tumour	13	1.47E+06	PDX tumour	99.38	
**Unknown 69**	PDX tumour	13	1.94E+06	PDX tumour	99.39	
**Unknown 69**	PDX tumour	8	1.39E+06	PDX tumour	99.01	
**Unknown 69**	PDX tumour	11	1.44E+06	PDX tumour	99.3	
**Unknown 69**	PDX tumour	7	1.05E+06	PDX tumour	99.03	
**Unknown 69**	PDX tumour	8	1.84E+06	PDX tumour	99.32	
**Unknown 69**	PDX tumour	8	8.73E+05	PDX tumour	99.15	
**Unknown 69**	PDX tumour	10	1.81E+06	PDX tumour	99.38	
**Unknown 69**	PDX tumour	12	1.58E+06	PDX tumour	99.32	
**Unknown 69**	PDX tumour	9	1.11E+06	PDX tumour	99.34	
**Unknown 69**	PDX tumour	10	2.05E+06	PDX tumour	99.37	
**Unknown 70**	PDX tumour	12	1.72E+06	PDX tumour	99.38	100.00
**Unknown 70**	PDX tumour	9	1.44E+06	PDX tumour	99.37	
**Unknown 70**	PDX tumour	12	1.64E+06	PDX tumour	99.34	
**Unknown 70**	PDX tumour	8	9.35E+05	PDX tumour	98.45	
**Unknown 70**	PDX tumour	12	1.64E+06	PDX tumour	99.38	
**Unknown 70**	PDX tumour	12	3.38E+06	PDX tumour	99.38	
**Unknown 70**	PDX tumour	13	2.10E+06	PDX tumour	99.38	
**Unknown 70**	PDX tumour	12	1.37E+06	PDX tumour	99.3	
**Unknown 70**	PDX tumour	12	2.82E+06	PDX tumour	99.37	
**Unknown 70**	PDX tumour	11	1.92E+06	PDX tumour	99.38	
**Unknown 70**	PDX tumour	11	1.86E+06	PDX tumour	99.17	
**Unknown 70**	PDX tumour	11	2.15E+06	PDX tumour	99.38	
**Unknown 70**	PDX tumour	11	1.69E+06	PDX tumour	99.39	
**Unknown 70**	PDX tumour	12	1.99E+06	PDX tumour	99.35	
**Unknown 70**	PDX tumour	9	2.42E+06	PDX tumour	99.36	
**Unknown 70**	PDX tumour	12	1.61E+06	PDX tumour	99.39	
**Unknown 70**	PDX tumour	7	1.50E+06	PDX tumour	99.37	
**Unknown 70**	PDX tumour	9	1.87E+06	PDX tumour	99.37	
**Unknown 70**	PDX tumour	11	2.96E+06	PDX tumour	99.39	
**Unknown 70**	PDX tumour	7	1.66E+06	PDX tumour	99.39	
**Unknown 71**	PDX tumour	10	3.41E+06	PDX tumour	99.37	100.00
**Unknown 71**	PDX tumour	10	2.62E+06	PDX tumour	99.38	
**Unknown 71**	PDX tumour	10	2.83E+06	PDX tumour	99.37	
**Unknown 71**	PDX tumour	9	2.01E+06	PDX tumour	99.38	
**Unknown 71**	PDX tumour	12	3.41E+06	PDX tumour	99.39	
**Unknown 71**	PDX tumour	12	2.73E+06	PDX tumour	99.29	
**Unknown 71**	PDX tumour	11	2.46E+06	PDX tumour	99.39	

**Unknown 71**	PDX tumour	11	2.42E+06	PDX tumour	99.38	
**Unknown 71**	PDX tumour	10	3.23E+06	PDX tumour	99.34	
**Unknown 71**	PDX tumour	9	2.29E+06	PDX tumour	99.38	
**Unknown 71**	PDX tumour	12	3.38E+06	PDX tumour	99.32	
**Unknown 71**	PDX tumour	11	2.45E+06	PDX tumour	99.38	
**Unknown 71**	PDX tumour	9	1.37E+06	PDX tumour	99.39	
**Unknown 71**	PDX tumour	11	1.52E+06	PDX tumour	99.38	
**Unknown 71**	PDX tumour	10	3.70E+06	PDX tumour	99.37	
**Unknown 71**	PDX tumour	9	2.99E+06	PDX tumour	99.38	
**Unknown 71**	PDX tumour	12	2.48E+06	PDX tumour	99.37	
**Unknown 71**	PDX tumour	11	2.81E+06	PDX tumour	99.38	
**Unknown 71**	PDX tumour	13	3.14E+06	PDX tumour	99.37	
**Unknown 71**	PDX tumour	13	3.34E+06	PDX tumour	99.39	
**Unknown 72**	PDX tumour	13	2.36E+06	PDX tumour	99.38	100.00
**Unknown 72**	PDX tumour	13	2.28E+06	PDX tumour	99.34	
**Unknown 72**	PDX tumour	11	3.41E+06	PDX tumour	99.38	
**Unknown 72**	PDX tumour	12	2.65E+06	PDX tumour	99.37	
**Unknown 73**	Lymphoma	9	3.84E+06	Lymphoma	97.97	100.00
**Unknown 73**	Lymphoma	11	3.68E+06	Lymphoma	99.21	
**Unknown 73**	Lymphoma	10	3.82E+06	Lymphoma	99.3	
**Unknown 73**	Lymphoma	10	2.82E+06	Lymphoma	98.22	
**Unknown 73**	Lymphoma	10	3.80E+06	Lymphoma	99.3	
**Unknown 73**	Lymphoma	11	2.84E+06	Lymphoma	98.73	
**Unknown 73**	Lymphoma	11	4.40E+06	Lymphoma	98.81	
**Unknown 73**	Lymphoma	7	2.86E+06	Lymphoma	99.21	
**Unknown 73**	Lymphoma	12	3.03E+06	Lymphoma	98.7	
**Unknown 74**	PDX tumour	13	2.20E+06	PDX tumour	99.31	100.00
**Unknown 74**	PDX tumour	13	2.15E+06	PDX tumour	99.38	
**Unknown 74**	PDX tumour	15	1.62E+06	PDX tumour	99.11	
**Unknown 74**	PDX tumour	11	2.73E+06	PDX tumour	99.32	
**Unknown 74**	PDX tumour	13	3.17E+06	PDX tumour	99.31	
**Unknown 74**	PDX tumour	7	1.22E+06	PDX tumour	99.31	
**Unknown 74**	PDX tumour	10	2.13E+06	PDX tumour	99.38	
**Unknown 74**	PDX tumour	15	2.33E+06	PDX tumour	99.3	
**Unknown 74**	PDX tumour	10	2.57E+06	PDX tumour	99.38	
**Unknown 74**	PDX tumour	15	3.38E+06	PDX tumour	99.37	
**Unknown 74**	PDX tumour	11	1.09E+06	PDX tumour	99.19	
**Unknown 74**	PDX tumour	15	1.51E+06	PDX tumour	99.33	
**Unknown 74**	PDX tumour	15	3.54E+06	PDX tumour	99.28	
**Unknown 74**	PDX tumour	6	4.69E+06	PDX tumour	99.28	
**Unknown 75**	Lymphoma	13	3.15E+06	Lymphoma	98.47	100.00
**Unknown 75**	Lymphoma	11	2.46E+06	Lymphoma	97.51	

**Unknown 75**	Lymphoma	10	2.22E+06	Lymphoma	98.61	
**Unknown 75**	Lymphoma	13	2.19E+06	Lymphoma	98.39	
**Unknown 75**	Lymphoma	8	1.81E+06	Lymphoma	99.08	
**Unknown 75**	Lymphoma	9	1.98E+06	Lymphoma	98.95	
**Unknown 75**	Lymphoma	9	2.20E+06	Lymphoma	99.2	
**Unknown 75**	Lymphoma	8	1.63E+06	Lymphoma	98.7	
**Unknown 75**	Lymphoma	10	1.95E+06	Lymphoma	98.59	
**Unknown 75**	Lymphoma	9	1.87E+06	Lymphoma	98.43	
**Unknown 75**	Lymphoma	9	1.15E+06	Unclassifiable	-	
**Unknown 75**	Lymphoma	8	1.78E+06	Lymphoma	98.79	
**Unknown 75**	Lymphoma	11	2.69E+06	Lymphoma	99.25	
**Unknown 76**	PDX tumour	9	3.42E+06	PDX tumour	99.25	100.00
**Unknown 76**	PDX tumour	9	2.20E+06	PDX tumour	99.38	
**Unknown 76**	PDX tumour	10	4.89E+06	PDX tumour	99.38	
**Unknown 76**	PDX tumour	11	2.78E+06	PDX tumour	99.37	
**Unknown 76**	PDX tumour	9	4.26E+06	PDX tumour	99.38	
**Unknown 76**	PDX tumour	13	2.16E+06	PDX tumour	99.38	
**Unknown 76**	PDX tumour	8	2.51E+06	PDX tumour	99.38	
**Unknown 76**	PDX tumour	8	3.00E+06	PDX tumour	99.38	
**Unknown 76**	PDX tumour	8	2.13E+06	PDX tumour	99.38	
**Unknown 76**	PDX tumour	9	1.90E+06	PDX tumour	99.38	
**Unknown 76**	PDX tumour	9	3.40E+06	PDX tumour	99.36	
**Unknown 76**	PDX tumour	6	1.43E+06	PDX tumour	99.28	
**Unknown 77**	PDX tumour	11	4.01E+06	PDX tumour	99.37	100.00
**Unknown 77**	PDX tumour	9	2.03E+06	PDX tumour	99.39	
**Unknown 77**	PDX tumour	11	1.72E+06	PDX tumour	99.37	
**Unknown 77**	PDX tumour	12	2.18E+06	PDX tumour	99.38	
**Unknown 77**	PDX tumour	11	2.72E+06	PDX tumour	99.33	
**Unknown 77**	PDX tumour	11	1.52E+06	PDX tumour	99.23	
**Unknown 77**	PDX tumour	12	8.12E+05	PDX tumour	99.39	
**Unknown 77**	PDX tumour	8	1.47E+06	PDX tumour	99.38	
**Unknown 77**	PDX tumour	9	2.54E+06	PDX tumour	99.39	
**Unknown 77**	PDX tumour	10	3.47E+06	PDX tumour	99.39	
**Unknown 77**	PDX tumour	8	2.36E+06	PDX tumour	99.39	
**Unknown 77**	PDX tumour	2	1.47E+05	bad data	-	
**Unknown 77**	PDX tumour	8	1.44E+06	PDX tumour	99.39	
**Unknown 77**	PDX tumour	10	1.91E+06	PDX tumour	99.36	
**Unknown 77**	PDX tumour	10	2.26E+06	PDX tumour	99.37	
**Unknown 77**	PDX tumour	8	1.45E+06	PDX tumour	99.38	
**Unknown 77**	PDX tumour	7	2.09E+06	PDX tumour	99.39	
**Unknown 77**	PDX tumour	8	2.66E+06	PDX tumour	99.39	
**Unknown 77**	PDX tumour	9	2.80E+06	PDX tumour	99.37	

**Unknown 77**	PDX tumour	12	5.47E+06	PDX tumour	99.39	
**Unknown 77**	PDX tumour	8	1.01E+06	PDX tumour	99.38	
**Unknown 77**	PDX tumour	1	3.05E+05	bad data	-	
**Unknown 78**	PDX tumour	13	4.10E+06	PDX tumour	98.41	100.00
**Unknown 78**	PDX tumour	14	3.95E+06	PDX tumour	99.33	
**Unknown 78**	PDX tumour	14	3.84E+06	PDX tumour	99.08	
**Unknown 78**	PDX tumour	14	5.18E+06	Unclassifiable	-	
**Unknown 78**	PDX tumour	14	3.75E+06	PDX tumour	98.27	
**Unknown 78**	PDX tumour	12	3.37E+06	PDX tumour	99.17	
**Unknown 78**	PDX tumour	10	3.58E+06	PDX tumour	98.96	
**Unknown 78**	PDX tumour	16	6.03E+06	PDX tumour	99.11	
**Unknown 78**	PDX tumour	13	2.20E+06	PDX tumour	99.36	
**Unknown 78**	PDX tumour	9	1.75E+06	PDX tumour	99.3	
**Unknown 78**	PDX tumour	8	1.71E+06	PDX tumour	99.29	
**Unknown 79**	PDX tumour	12	1.91E+06	PDX tumour	99.38	100.00
**Unknown 79**	PDX tumour	11	1.43E+06	PDX tumour	99.34	
**Unknown 79**	PDX tumour	9	1.06E+06	PDX tumour	99.38	
**Unknown 79**	PDX tumour	10	1.41E+06	PDX tumour	99.34	
**Unknown 79**	PDX tumour	11	1.46E+06	PDX tumour	99.38	
**Unknown 79**	PDX tumour	11	1.38E+06	PDX tumour	99.2	
**Unknown 79**	PDX tumour	11	2.31E+06	PDX tumour	99.39	
**Unknown 79**	PDX tumour	11	1.81E+06	PDX tumour	99.36	
**Unknown 79**	PDX tumour	12	1.60E+06	PDX tumour	99.38	
**Unknown 79**	PDX tumour	11	2.05E+06	PDX tumour	99.37	
**Unknown 79**	PDX tumour	11	1.94E+06	PDX tumour	99.34	
**Unknown 79**	PDX tumour	10	2.04E+06	PDX tumour	99.37	
**Unknown 79**	PDX tumour	12	1.92E+06	PDX tumour	99.37	
**Unknown 79**	PDX tumour	10	2.50E+06	PDX tumour	99.38	
**Unknown 79**	PDX tumour	12	1.48E+06	PDX tumour	99.39	
**Unknown 79**	PDX tumour	3	5.88E+05	bad data	-	
**Unknown 79**	PDX tumour	8	2.08E+06	PDX tumour	99.38	
**Unknown 79**	PDX tumour	12	2.89E+06	PDX tumour	99.35	
**Unknown 80**	PDX tumour	10	4.42E+06	PDX tumour	99.38	100.00
**Unknown 80**	PDX tumour	10	2.35E+06	PDX tumour	99.34	
**Unknown 80**	PDX tumour	9	2.11E+06	PDX tumour	99.39	
**Unknown 80**	PDX tumour	9	1.94E+06	PDX tumour	99.36	
**Unknown 80**	PDX tumour	12	3.68E+06	PDX tumour	99.35	
**Unknown 80**	PDX tumour	10	3.68E+06	PDX tumour	99.37	
**Unknown 80**	PDX tumour	10	3.66E+06	PDX tumour	99.33	
**Unknown 80**	PDX tumour	10	3.14E+06	PDX tumour	99.28	
**Unknown 80**	PDX tumour	9	2.72E+06	PDX tumour	99.38	
**Unknown 80**	PDX tumour	10	1.70E+06	PDX tumour	99.38	

**Unknown 80**	PDX tumour	8	1.96E+06	PDX tumour	99.37	
**Unknown 80**	PDX tumour	10	1.37E+06	PDX tumour	99.35	
**Unknown 80**	PDX tumour	12	4.34E+06	PDX tumour	99.31	
**Unknown 80**	PDX tumour	12	6.81E+06	PDX tumour	99.39	
**Unknown 80**	PDX tumour	1	4.02E+05	bad data	-	
**Unknown 81**	PDX tumour	12	2.25E+06	PDX tumour	99.34	100.00
**Unknown 81**	PDX tumour	10	9.46E+05	PDX tumour	99.35	
**Unknown 81**	PDX tumour	7	9.46E+05	PDX tumour	98.88	
**Unknown 81**	PDX tumour	11	1.36E+06	PDX tumour	99.38	
**Unknown 81**	PDX tumour	11	1.09E+06	PDX tumour	99.39	
**Unknown 81**	PDX tumour	11	1.45E+06	PDX tumour	99.39	
**Unknown 81**	PDX tumour	11	1.55E+06	PDX tumour	99.38	
**Unknown 81**	PDX tumour	10	1.66E+06	PDX tumour	99.35	
**Unknown 81**	PDX tumour	10	1.12E+06	PDX tumour	99.38	
**Unknown 81**	PDX tumour	11	2.93E+06	PDX tumour	99.38	
**Unknown 81**	PDX tumour	11	4.85E+05	Unclassifiable	-	
**Unknown 81**	PDX tumour	14	2.05E+06	PDX tumour	99.29	
**Unknown 81**	PDX tumour	9	2.11E+06	PDX tumour	99.22	
**Unknown 81**	PDX tumour	9	3.02E+06	PDX tumour	99.36	
**Unknown 81**	PDX tumour	12	3.41E+06	PDX tumour	99.35	
**Unknown 81**	PDX tumour	14	3.39E+06	PDX tumour	99.38	
**Unknown 81**	PDX tumour	10	4.01E+06	PDX tumour	99.36	
**Unknown 82**	PDX tumour	13	9.02E+06	PDX tumour	99.37	100.00
**Unknown 82**	PDX tumour	14	3.75E+06	PDX tumour	98.96	
**Unknown 82**	PDX tumour	11	3.07E+06	PDX tumour	99.19	
**Unknown 82**	PDX tumour	11	1.08E+06	PDX tumour	98.18	
**Unknown 82**	PDX tumour	16	3.17E+06	PDX tumour	98.51	
**Unknown 82**	PDX tumour	13	3.32E+06	PDX tumour	99.35	
**Unknown 82**	PDX tumour	13	3.19E+06	PDX tumour	99.29	
**Unknown 82**	PDX tumour	13	1.79E+06	PDX tumour	98.69	
**Unknown 82**	PDX tumour	12	1.21E+06	PDX tumour	99.38	
**Unknown 82**	PDX tumour	12	2.51E+06	Unclassifiable	-	
**Unknown 82**	PDX tumour	12	2.48E+06	PDX tumour	98.08	
**Unknown 82**	PDX tumour	9	2.68E+06	PDX tumour	98.68	
**Unknown 82**	PDX tumour	12	2.43E+06	Unclassifiable	-	
**Unknown 83**	PDX tumour	11	5.44E+06	PDX tumour	99.32	100.00
**Unknown 83**	PDX tumour	12	8.59E+06	PDX tumour	99.36	
**Unknown 83**	PDX tumour	11	6.26E+06	PDX tumour	99.36	
**Unknown 83**	PDX tumour	16	3.27E+06	PDX tumour	99.35	
**Unknown 83**	PDX tumour	11	5.32E+06	PDX tumour	99.3	
**Unknown 83**	PDX tumour	11	4.53E+06	PDX tumour	99.35	
**Unknown 83**	PDX tumour	7	1.06E+06	PDX tumour	99.37	

**Unknown 83**	PDX tumour	12	9.95E+06	Unclassifiable	-	
**Unknown 83**	PDX tumour	12	1.33E+07	PDX tumour	99.37	
**Unknown 83**	PDX tumour	15	4.61E+06	PDX tumour	99.36	
**Unknown 83**	PDX tumour	16	3.11E+06	PDX tumour	99.37	
**Unknown 83**	PDX tumour	16	7.25E+06	PDX tumour	99.35	
**Unknown 83**	PDX tumour	16	5.56E+06	PDX tumour	99.37	
**Unknown 83**	PDX tumour	12	9.52E+06	PDX tumour	99.38	
**Unknown 83**	PDX tumour	13	1.22E+07	PDX tumour	99.28	
**Unknown 83**	PDX tumour	10	3.92E+06	PDX tumour	99.38	
**Unknown 83**	PDX tumour	13	5.12E+06	PDX tumour	99.38	
**Unknown 83**	PDX tumour	13	6.41E+06	PDX tumour	99.39	
**Unknown 83**	PDX tumour	11	5.51E+06	PDX tumour	97.74	
**Unknown 83**	PDX tumour	12	5.82E+06	PDX tumour	99.38	
**Unknown 83**	PDX tumour	10	1.03E+07	PDX tumour	99.23	
**Unknown 84**	PDX tumour	15	7.09E+06	PDX tumour	99.37	100.00
**Unknown 84**	PDX tumour	13	5.61E+06	PDX tumour	99.38	
**Unknown 84**	PDX tumour	14	3.44E+06	PDX tumour	99.38	
**Unknown 84**	PDX tumour	12	3.75E+06	PDX tumour	99.39	
**Unknown 84**	PDX tumour	14	3.30E+06	PDX tumour	99.29	
**Unknown 84**	PDX tumour	12	5.08E+06	PDX tumour	99.38	
**Unknown 84**	PDX tumour	12	4.86E+06	PDX tumour	99.38	
**Unknown 84**	PDX tumour	11	4.02E+06	PDX tumour	99.38	
**Unknown 84**	PDX tumour	10	4.48E+06	PDX tumour	99.38	
**Unknown 84**	PDX tumour	10	2.92E+06	PDX tumour	99.36	
**Unknown 84**	PDX tumour	11	4.79E+06	PDX tumour	99.36	
**Unknown 84**	PDX tumour	14	7.14E+06	PDX tumour	99.38	
**Unknown 84**	PDX tumour	11	4.64E+06	PDX tumour	99.39	
**Unknown 84**	PDX tumour	13	3.67E+06	PDX tumour	99.37	
**Unknown 84**	PDX tumour	11	5.57E+06	PDX tumour	99.37	
**Unknown 84**	PDX tumour	10	5.56E+06	PDX tumour	99.34	
**Unknown 84**	PDX tumour	8	5.44E+06	PDX tumour	99.37	
**Unknown 85**	PDX tumour	10	3.90E+06	PDX tumour	99.38	100.00
**Unknown 85**	PDX tumour	10	8.69E+06	PDX tumour	98.97	
**Unknown 85**	PDX tumour	12	5.71E+06	PDX tumour	99.34	
**Unknown 85**	PDX tumour	10	1.55E+06	PDX tumour	99.39	
**Unknown 85**	PDX tumour	15	5.29E+06	PDX tumour	99.39	
**Unknown 85**	PDX tumour	11	3.84E+06	PDX tumour	99.3	
**Unknown 85**	PDX tumour	11	5.84E+06	PDX tumour	99.35	
**Unknown 85**	PDX tumour	10	3.62E+06	PDX tumour	99.37	
**Unknown 85**	PDX tumour	10	2.81E+06	PDX tumour	99.38	
**Unknown 85**	PDX tumour	12	7.01E+06	PDX tumour	99.34	
**Unknown 85**	PDX tumour	11	3.97E+06	PDX tumour	99.25	

**Unknown 85**	PDX tumour	10	3.22E+06	PDX tumour	99.38	
**Unknown 85**	PDX tumour	10	1.90E+06	PDX tumour	99.34	
**Unknown 85**	PDX tumour	10	3.81E+06	PDX tumour	99.38	
**Unknown 85**	PDX tumour	11	5.24E+06	PDX tumour	99.35	
**Unknown 85**	PDX tumour	12	2.66E+06	PDX tumour	99.35	
**Unknown 85**	PDX tumour	15	1.10E+07	PDX tumour	99.35	
**Unknown 85**	PDX tumour	12	8.02E+06	PDX tumour	99.31	
**Unknown 85**	PDX tumour	13	8.34E+06	PDX tumour	99.36	
**Unknown 85**	PDX tumour	10	4.48E+06	PDX tumour	99.29	
**Unknown 85**	PDX tumour	10	3.17E+06	PDX tumour	99.3	
**Unknown 85**	PDX tumour	10	6.39E+06	PDX tumour	99.38	
**Unknown 85**	PDX tumour	11	5.53E+06	PDX tumour	99.39	
**Unknown 85**	PDX tumour	11	6.77E+06	PDX tumour	99.38	
**Unknown 85**	PDX tumour	10	8.27E+06	PDX tumour	99.38	
**Unknown 85**	PDX tumour	10	6.22E+06	PDX tumour	99.38	
**Unknown 85**	PDX tumour	10	4.42E+06	PDX tumour	99.36	
**Unknown 85**	PDX tumour	9	3.41E+06	PDX tumour	99.37	
**Unknown 85**	PDX tumour	9	6.74E+06	PDX tumour	99.37	
**Unknown 85**	PDX tumour	11	8.58E+06	PDX tumour	99.39	
**Unknown 85**	PDX tumour	11	1.25E+07	PDX tumour	99.38	
**Unknown 85**	PDX tumour	15	6.75E+06	PDX tumour	99.3	
**Unknown 85**	PDX tumour	9	4.06E+06	PDX tumour	99.38	
**Unknown 85**	PDX tumour	15	1.76E+06	PDX tumour	99.38	
**Unknown 85**	PDX tumour	9	1.48E+06	PDX tumour	99.37	
**Unknown 85**	PDX tumour	5	3.85E+05	PDX tumour	99.14	
**Unknown 85**	PDX tumour	8	1.15E+06	PDX tumour	99.33	
**Unknown 85**	PDX tumour	11	1.19E+06	PDX tumour	99.39	
**Unknown 85**	PDX tumour	10	1.51E+06	PDX tumour	99.39	
**Unknown 85**	PDX tumour	11	3.14E+06	PDX tumour	99.32	
**Unknown 86**	PDX tumour	11	5.21E+06	PDX tumour	99.36	100.00
**Unknown 86**	PDX tumour	13	4.46E+06	PDX tumour	99.39	
**Unknown 86**	PDX tumour	11	5.53E+06	PDX tumour	99.38	
**Unknown 86**	PDX tumour	12	5.07E+06	PDX tumour	99.38	
**Unknown 86**	PDX tumour	12	4.63E+06	PDX tumour	99.39	
**Unknown 86**	PDX tumour	12	4.21E+06	PDX tumour	99.38	
**Unknown 86**	PDX tumour	11	3.38E+06	PDX tumour	99.35	
**Unknown 86**	PDX tumour	10	4.99E+06	PDX tumour	99.26	
**Unknown 87**	Lymphoma	9	2.84E+06	Lymphoma	99.23	100.00
**Unknown 87**	Lymphoma	10	3.53E+06	Lymphoma	99.34	
**Unknown 87**	Lymphoma	10	4.40E+06	Lymphoma	99.38	
**Unknown 87**	Lymphoma	10	4.02E+06	Lymphoma	99.38	
**Unknown 87**	Lymphoma	9	2.79E+06	Lymphoma	98.46	

**Unknown 87**	Lymphoma	10	3.58E+06	Lymphoma	99.06	
**Unknown 87**	Lymphoma	7	6.10E+05	Lymphoma	99.14	
**Unknown 87**	Lymphoma	9	3.72E+06	Lymphoma	98.57	
**Unknown 87**	Lymphoma	10	3.35E+06	Lymphoma	99.31	
**Unknown 87**	Lymphoma	10	3.24E+06	Lymphoma	99.28	
**Unknown 87**	Lymphoma	10	2.96E+06	Lymphoma	99.34	
**Unknown 87**	Lymphoma	10	4.19E+06	Lymphoma	99.17	
**Unknown 87**	Lymphoma	10	3.63E+06	Lymphoma	99.22	
**Unknown 87**	Lymphoma	10	2.04E+06	Lymphoma	99.39	
**Unknown 87**	Lymphoma	9	2.95E+06	Lymphoma	99.38	
**Unknown 87**	Lymphoma	9	2.58E+06	Lymphoma	99.36	
**Unknown 88**	PDX tumour	14	4.01E+06	PDX tumour	99.38	100.00
**Unknown 88**	PDX tumour	10	1.39E+06	PDX tumour	99.39	
**Unknown 88**	PDX tumour	16	3.10E+06	PDX tumour	99.34	
**Unknown 88**	PDX tumour	15	2.70E+06	PDX tumour	99.36	
**Unknown 88**	PDX tumour	15	4.70E+06	PDX tumour	99.39	
**Unknown 88**	PDX tumour	12	3.13E+06	PDX tumour	99.38	
**Unknown 88**	PDX tumour	12	2.66E+06	PDX tumour	99.37	
**Unknown 88**	PDX tumour	12	3.07E+06	PDX tumour	99.36	
**Unknown 88**	PDX tumour	14	3.71E+06	PDX tumour	99.38	
**Unknown 88**	PDX tumour	16	4.37E+06	PDX tumour	99.38	
**Unknown 88**	PDX tumour	13	3.94E+06	PDX tumour	99.38	
**Unknown 88**	PDX tumour	14	2.23E+06	PDX tumour	99.38	
**Unknown 88**	PDX tumour	14	2.26E+06	PDX tumour	99.35	
**Unknown 89**	PDX tumour	10	3.40E+06	PDX tumour	99.22	100.00
**Unknown 89**	PDX tumour	10	2.79E+06	PDX tumour	99.37	
**Unknown 89**	PDX tumour	11	9.48E+05	PDX tumour	99.35	
**Unknown 89**	PDX tumour	7	1.27E+06	PDX tumour	99.31	
**Unknown 90**	PDX tumour	17	9.18E+06	Unclassifiable	-	85.71
**Unknown 90**	PDX tumour	17	6.50E+06	Unclassifiable	-	
**Unknown 90**	PDX tumour	12	3.25E+06	PDX tumour	97.85	
**Unknown 90**	PDX tumour	13	4.62E+06	Unclassifiable	-	
**Unknown 90**	PDX tumour	14	4.49E+06	Unclassifiable	-	
**Unknown 90**	PDX tumour	14	5.11E+06	PDX tumour	98.33	
**Unknown 90**	PDX tumour	15	4.80E+06	PDX tumour	98.24	
**Unknown 90**	PDX tumour	16	4.95E+06	PDX tumour	98.2	
**Unknown 90**	PDX tumour	15	2.32E+06	Lymphoma	97.58	
**Unknown 90**	PDX tumour	15	1.67E+06	Unclassifiable	-	
**Unknown 90**	PDX tumour	11	2.02E+06	PDX tumour	98.85	
**Unknown 90**	PDX tumour	9	2.15E+06	PDX tumour	99.12	
**Unknown 91**	PDX tumour	11	2.31E+06	PDX tumour	99.38	100.00
**Unknown 91**	PDX tumour	9	6.15E+05	PDX tumour	99.33	

**Unknown 91**	PDX tumour	9	3.54E+06	PDX tumour	99.31	
**Unknown 91**	PDX tumour	11	3.74E+06	PDX tumour	99.24	
**Unknown 91**	PDX tumour	12	3.01E+06	PDX tumour	99.33	
**Unknown 91**	PDX tumour	12	2.08E+06	PDX tumour	99.38	
**Unknown 91**	PDX tumour	11	3.44E+06	PDX tumour	99.21	
**Unknown 91**	PDX tumour	11	2.13E+06	PDX tumour	99.38	
**Unknown 92**	PDX tumour	9	2.02E+06	PDX tumour	99.33	100.00
**Unknown 92**	PDX tumour	9	2.24E+06	PDX tumour	99.21	
**Unknown 92**	PDX tumour	9	4.37E+06	PDX tumour	99.38	
**Unknown 92**	PDX tumour	11	5.00E+06	PDX tumour	99.31	
**Unknown 92**	PDX tumour	10	1.90E+06	PDX tumour	99.23	
**Unknown 92**	PDX tumour	10	2.65E+06	PDX tumour	99.38	
**Unknown 92**	PDX tumour	11	4.22E+06	PDX tumour	99.38	
**Unknown 92**	PDX tumour	10	1.15E+06	PDX tumour	99.38	
**Unknown 92**	PDX tumour	10	1.29E+06	PDX tumour	99.29	
**Unknown 92**	PDX tumour	9	2.98E+06	PDX tumour	99.37	
**Unknown 92**	PDX tumour	11	8.18E+06	PDX tumour	99.39	
**Unknown 92**	PDX tumour	9	5.34E+06	PDX tumour	99.37	
**Unknown 92**	PDX tumour	9	3.10E+06	Unclassifiable	-	
**Unknown 93**	PDX tumour	10	3.27E+06	PDX tumour	98.67	95.83
**Unknown 93**	PDX tumour	9	2.76E+06	PDX tumour	99.24	
**Unknown 93**	PDX tumour	6	9.53E+05	PDX tumour	99.27	
**Unknown 93**	PDX tumour	9	3.03E+06	PDX tumour	99.33	
**Unknown 93**	PDX tumour	9	2.11E+06	PDX tumour	98.76	
**Unknown 93**	PDX tumour	10	2.43E+06	Unclassifiable	-	
**Unknown 93**	PDX tumour	7	1.82E+06	PDX tumour	99.22	
**Unknown 93**	PDX tumour	10	1.73E+06	PDX tumour	99.21	
**Unknown 93**	PDX tumour	8	9.33E+05	Unclassifiable	-	
**Unknown 93**	PDX tumour	9	3.23E+06	PDX tumour	99.25	
**Unknown 93**	PDX tumour	9	4.03E+06	PDX tumour	98.2	
**Unknown 93**	PDX tumour	8	2.71E+06	PDX tumour	99.02	
**Unknown 93**	PDX tumour	9	3.85E+06	PDX tumour	98.76	
**Unknown 93**	PDX tumour	8	3.30E+06	PDX tumour	98.6	
**Unknown 93**	PDX tumour	9	3.54E+06	PDX tumour	98.28	
**Unknown 93**	PDX tumour	8	2.39E+06	PDX tumour	99.25	
**Unknown 93**	PDX tumour	8	5.34E+06	PDX tumour	98.33	
**Unknown 93**	PDX tumour	8	2.82E+06	PDX tumour	97.66	
**Unknown 93**	PDX tumour	9	3.57E+06	PDX tumour	98.56	
**Unknown 93**	PDX tumour	8	1.60E+06	PDX tumour	98.63	
**Unknown 93**	PDX tumour	9	3.88E+06	PDX tumour	98.47	
**Unknown 93**	PDX tumour	9	2.70E+06	PDX tumour	98.64	
**Unknown 93**	PDX tumour	6	8.19E+05	Lymphoma	96.74	

**Unknown 93**	PDX tumour	8	3.72E+06	PDX tumour	97.75	
**Unknown 93**	PDX tumour	7	2.31E+06	PDX tumour	98.98	
**Unknown 93**	PDX tumour	7	1.31E+06	PDX tumour	98.73	
**Unknown 93**	PDX tumour	6	7.16E+05	Unclassifiable	-	
**Unknown 94**	Lymphoma	12	4.71E+06	Lymphoma	99.12	100.00
**Unknown 94**	Lymphoma	6	1.07E+06	bad data	-	
**Unknown 94**	Lymphoma	2	2.15E+05	bad data	-	
**Unknown 94**	Lymphoma	6	1.24E+06	Lymphoma	99.37	
**Unknown 94**	Lymphoma	13	2.00E+06	Lymphoma	99.17	
**Unknown 94**	Lymphoma	11	9.38E+05	bad data	-	
**Unknown 94**	Lymphoma	12	1.57E+06	Lymphoma	99.31	
**Unknown 95**	PDX tumour	10	3.12E+06	PDX tumour	99.38	100.00
**Unknown 95**	PDX tumour	10	1.70E+06	PDX tumour	99.33	
**Unknown 95**	PDX tumour	8	9.48E+05	PDX tumour	99.15	
**Unknown 95**	PDX tumour	10	1.75E+06	PDX tumour	99.13	
**Unknown 95**	PDX tumour	11	3.19E+06	PDX tumour	99.33	
**Unknown 95**	PDX tumour	10	1.75E+06	PDX tumour	99.38	
**Unknown 95**	PDX tumour	9	1.94E+06	PDX tumour	99.39	
**Unknown 95**	PDX tumour	9	2.25E+06	PDX tumour	99.31	
**Unknown 95**	PDX tumour	7	1.55E+06	PDX tumour	99.37	
**Unknown 95**	PDX tumour	10	3.38E+06	PDX tumour	99.37	
**Unknown 96**	Lymphoma	13	9.01E+06	Lymphoma	99.3	100.00
**Unknown 96**	Lymphoma	14	1.14E+07	Lymphoma	99.05	
**Unknown 96**	Lymphoma	14	1.49E+07	Lymphoma	98.99	
**Unknown 96**	Lymphoma	14	1.63E+07	Unclassifiable	-	
**Unknown 96**	Lymphoma	14	1.51E+07	Lymphoma	99.02	
**Unknown 96**	Lymphoma	14	8.44E+06	Lymphoma	99.27	
**Unknown 96**	Lymphoma	14	6.98E+06	Lymphoma	99.28	
**Unknown 96**	Lymphoma	14	5.46E+06	Lymphoma	99.38	
**Unknown 96**	Lymphoma	14	7.90E+06	Lymphoma	99.36	
**Unknown 96**	Lymphoma	14	7.40E+06	Lymphoma	99.38	
**Unknown 96**	Lymphoma	8	4.47E+06	Lymphoma	99.35	
**Unknown 96**	Lymphoma	8	2.40E+06	Lymphoma	99.29	
**Unknown 96**	Lymphoma	13	6.59E+06	Lymphoma	99.32	
**Unknown 96**	Lymphoma	12	3.06E+06	Lymphoma	99.38	
**Unknown 97**	PDX tumour	12	2.86E+06	PDX tumour	99.37	100.00
**Unknown 97**	PDX tumour	11	2.18E+06	PDX tumour	99.38	
**Unknown 97**	PDX tumour	10	2.39E+06	PDX tumour	98.73	
**Unknown 97**	PDX tumour	12	5.68E+06	PDX tumour	99.18	
**Unknown 97**	PDX tumour	15	3.56E+06	PDX tumour	99.14	
**Unknown 97**	PDX tumour	12	2.41E+06	PDX tumour	99.36	
**Unknown 97**	PDX tumour	10	2.17E+06	PDX tumour	99.38	

**Unknown 97**	PDX tumour	12	3.49E+06	PDX tumour	99.39	
**Unknown 97**	PDX tumour	13	3.17E+06	PDX tumour	99.39	
**Unknown 97**	PDX tumour	11	2.78E+06	PDX tumour	99.38	
**Unknown 98**	PDX tumour	10	1.17E+06	PDX tumour	98.78	100.00
**Unknown 98**	PDX tumour	8	8.46E+05	PDX tumour	99.13	
**Unknown 98**	PDX tumour	9	2.43E+06	PDX tumour	99.16	
**Unknown 98**	PDX tumour	8	1.09E+06	PDX tumour	99.05	
**Unknown 98**	PDX tumour	11	1.67E+06	PDX tumour	99.35	
**Unknown 98**	PDX tumour	1	3.45E+05	bad data	-	
**Unknown 98**	PDX tumour	11	2.17E+06	PDX tumour	99.26	
**Unknown 98**	PDX tumour	6	1.31E+06	PDX tumour	99.25	
**Unknown 98**	PDX tumour	10	2.34E+06	PDX tumour	99.17	
**Unknown 98**	PDX tumour	8	2.18E+06	PDX tumour	98.54	
**Unknown 98**	PDX tumour	10	1.40E+06	PDX tumour	99.25	
**Unknown 98**	PDX tumour	11	2.17E+06	PDX tumour	99.38	
**Unknown 98**	PDX tumour	10	5.02E+06	PDX tumour	99.35	
**Unknown 98**	PDX tumour	8	2.83E+06	PDX tumour	99.33	
**Unknown 98**	PDX tumour	10	2.58E+06	PDX tumour	98.08	
**Unknown 98**	PDX tumour	6	1.68E+06	PDX tumour	99.25	
**Unknown 98**	PDX tumour	9	3.86E+06	PDX tumour	99.3	
**Unknown 99**	PDX tumour	8	4.46E+06	PDX tumour	99.31	100.00
**Unknown 99**	PDX tumour	6	3.50E+06	PDX tumour	99.28	
**Unknown 99**	PDX tumour	10	4.38E+06	PDX tumour	99.38	
**Unknown 99**	PDX tumour	11	4.42E+06	PDX tumour	99.24	
**Unknown 99**	PDX tumour	12	3.62E+06	PDX tumour	99.33	
**Unknown 99**	PDX tumour	11	4.38E+06	PDX tumour	99.38	
**Unknown 99**	PDX tumour	9	2.42E+06	PDX tumour	99.37	
**Unknown 99**	PDX tumour	9	2.65E+06	PDX tumour	99.23	
**Unknown 99**	PDX tumour	8	2.15E+06	PDX tumour	99.38	
**Unknown 99**	PDX tumour	8	2.07E+06	PDX tumour	99.36	
**Unknown 100**	PDX tumour	9	2.56E+06	PDX tumour	99.36	100.00
**Unknown 100**	PDX tumour	11	2.82E+06	PDX tumour	99.01	
**Unknown 100**	PDX tumour	10	3.89E+06	PDX tumour	99.39	
**Unknown 100**	PDX tumour	9	2.96E+06	PDX tumour	99.13	
**Unknown 100**	PDX tumour	11	3.25E+06	PDX tumour	99.08	
**Unknown 100**	PDX tumour	10	3.36E+06	PDX tumour	99.23	
**Unknown 100**	PDX tumour	10	2.95E+06	PDX tumour	99.25	
**Unknown 100**	PDX tumour	10	2.99E+06	PDX tumour	99.3	
**Unknown 100**	PDX tumour	9	3.88E+06	PDX tumour	98.32	
**Unknown 100**	PDX tumour	10	2.19E+06	PDX tumour	98.96	
**Unknown 100**	PDX tumour	9	3.16E+06	PDX tumour	99.04	
**Unknown 101**	Lymphoma	9	4.22E+06	Lymphoma	99.27	100.00

**Unknown 101**	Lymphoma	9	3.85E+06	Lymphoma	99.38	
**Unknown 101**	Lymphoma	8	1.70E+06	Lymphoma	<u>61.91</u>	
**Unknown 101**	Lymphoma	8	2.63E+06	Lymphoma	99.35	
**Unknown 101**	Lymphoma	9	4.80E+06	Lymphoma	99.32	
**Unknown 101**	Lymphoma	9	3.81E+06	Lymphoma	99.39	
**Unknown 101**	Lymphoma	10	3.83E+06	Lymphoma	99.38	
**Unknown 101**	Lymphoma	9	3.65E+06	Lymphoma	99.36	
**Unknown 101**	Lymphoma	8	5.03E+06	Lymphoma	99.26	
**Unknown 101**	Lymphoma	8	2.20E+06	Lymphoma	99.33	
**Unknown 101**	Lymphoma	9	5.36E+06	Lymphoma	99	
**Unknown 102**	Lymphoma	10	1.95E+06	Lymphoma	98.19	100.00
**Unknown 102**	Lymphoma	9	1.21E+06	Lymphoma	<u>54.99</u>	
**Unknown 102**	Lymphoma	10	1.88E+06	Lymphoma	<u>57.25</u>	
**Unknown 102**	Lymphoma	9	2.40E+06	Lymphoma	<u>75.03</u>	
**Unknown 102**	Lymphoma	9	2.29E+06	Lymphoma	98.23	
**Unknown 102**	Lymphoma	10	2.56E+06	Lymphoma	<u>80.69</u>	
**Unknown 102**	Lymphoma	11	3.08E+06	Lymphoma	99.08	
**Unknown 102**	Lymphoma	10	3.35E+06	Lymphoma	96.26	
**Unknown 102**	Lymphoma	11	4.52E+06	Lymphoma	97.12	
**Unknown 102**	Lymphoma	10	1.91E+06	Lymphoma	<u>84.36</u>	
**Unknown 102**	Lymphoma	9	3.30E+06	Lymphoma	98.04	
**Unknown 102**	Lymphoma	9	2.44E+06	Lymphoma	97.02	
**Unknown 102**	Lymphoma	1	2.90E+05	bad data	-	
**Unknown 102**	Lymphoma	10	3.05E+06	Lymphoma	97.53	
**Unknown 102**	Lymphoma	9	4.66E+06	Lymphoma	99.25	
**Unknown 102**	Lymphoma	10	5.64E+06	Lymphoma	98.2	
**Unknown 103**	PDX tumour	11	2.51E+06	PDX tumour	99.38	100.00
**Unknown 103**	PDX tumour	8	1.00E+06	PDX tumour	98.66	
**Unknown 103**	PDX tumour	12	4.19E+05	PDX tumour	99.23	
**Unknown 103**	PDX tumour	12	1.29E+06	PDX tumour	99.34	
**Unknown 103**	PDX tumour	12	1.93E+06	PDX tumour	99.29	
**Unknown 103**	PDX tumour	10	3.30E+06	PDX tumour	99.37	
**Unknown 103**	PDX tumour	9	2.12E+06	PDX tumour	99.3	
**Unknown 103**	PDX tumour	10	4.05E+06	PDX tumour	98.24	
**Unknown 103**	PDX tumour	10	3.85E+06	PDX tumour	99.21	
**Unknown 103**	PDX tumour	9	4.24E+06	Unclassifiable	-	
**Unknown 103**	PDX tumour	8	3.07E+06	PDX tumour	99.28	
**Unknown 104**	PDX tumour	9	9.49E+05	PDX tumour	98.7	94.44
**Unknown 104**	PDX tumour	10	6.70E+06	PDX tumour	98.87	
**Unknown 104**	PDX tumour	10	2.74E+06	PDX tumour	99.12	
**Unknown 104**	PDX tumour	8	2.44E+06	PDX tumour	99.31	
**Unknown 104**	PDX tumour	11	1.40E+06	Unclassifiable	-	

**Unknown 104**	PDX tumour	11	1.72E+06	PDX tumour	99.27	
**Unknown 104**	PDX tumour	8	1.53E+06	PDX tumour	99.38	
**Unknown 104**	PDX tumour	12	1.88E+06	Unclassifiable	-	
**Unknown 104**	PDX tumour	8	1.91E+06	PDX tumour	98.97	
**Unknown 104**	PDX tumour	9	5.52E+06	PDX tumour	98.18	
**Unknown 104**	PDX tumour	9	5.00E+06	Lymphoma	71.2	
**Unknown 104**	PDX tumour	8	6.61E+06	PDX tumour	97.63	
**Unknown 104**	PDX tumour	10	4.51E+06	Unclassifiable	-	
**Unknown 104**	PDX tumour	9	5.31E+06	Unclassifiable	-	
**Unknown 104**	PDX tumour	9	4.23E+06	PDX tumour	99.04	
**Unknown 104**	PDX tumour	8	2.56E+06	PDX tumour	99.27	
**Unknown 104**	PDX tumour	9	2.63E+06	Unclassifiable	-	
**Unknown 104**	PDX tumour	7	3.81E+06	Unclassifiable	-	
**Unknown 104**	PDX tumour	10	2.54E+06	PDX tumour	99.15	
**Unknown 104**	PDX tumour	9	1.12E+06	PDX tumour	98.34	
**Unknown 104**	PDX tumour	7	3.30E+06	Unclassifiable	-	
**Unknown 104**	PDX tumour	8	8.62E+05	PDX tumour	99.15	
**Unknown 104**	PDX tumour	9	2.94E+06	Unclassifiable	-	
**Unknown 104**	PDX tumour	8	2.22E+06	PDX tumour	99.06	
**Unknown 104**	PDX tumour	7	3.57E+06	Unclassifiable	-	
**Unknown 104**	PDX tumour	8	1.56E+06	PDX tumour	98.72	
**Unknown 104**	PDX tumour	4	6.74E+05	PDX tumour	98.7	
**Unknown 105**	Lymphoma	11	7.93E+06	Lymphoma	99.39	100.00
**Unknown 105**	Lymphoma	10	4.73E+06	Lymphoma	99.37	
**Unknown 105**	Lymphoma	9	4.46E+06	Lymphoma	99.38	
**Unknown 105**	Lymphoma	11	4.51E+06	Lymphoma	99.33	
**Unknown 105**	Lymphoma	9	6.72E+06	Lymphoma	99.38	
**Unknown 105**	Lymphoma	11	5.40E+06	Lymphoma	98.83	
**Unknown 105**	Lymphoma	10	4.37E+06	Lymphoma	99.14	
**Unknown 105**	Lymphoma	12	4.37E+06	Lymphoma	99.33	
**Unknown 105**	Lymphoma	12	7.42E+06	Lymphoma	99.38	
**Unknown 105**	Lymphoma	1	1.90E+05	bad data	-	
**Unknown 105**	Lymphoma	11	4.94E+06	Lymphoma	99.34	
**Unknown 105**	Lymphoma	12	5.46E+06	Lymphoma	99.23	
**Unknown 105**	Lymphoma	11	2.95E+06	Lymphoma	97.03	
**Unknown 105**	Lymphoma	10	5.96E+06	Lymphoma	98.54	
**Unknown 106**	Lymphoma	10	3.56E+06	Lymphoma	97.91	100.00
**Unknown 106**	Lymphoma	9	2.10E+06	Lymphoma	97.98	
**Unknown 106**	Lymphoma	11	2.07E+06	Lymphoma	97.01	
**Unknown 106**	Lymphoma	10	1.66E+06	Lymphoma	93.55	
**Unknown 106**	Lymphoma	12	2.04E+06	Lymphoma	95	
**Unknown 106**	Lymphoma	11	2.50E+06	Lymphoma	96.6	

**Unknown 106**	Lymphoma	11	1.87E+06	Lymphoma	98.54	
**Unknown 106**	Lymphoma	13	3.78E+06	Lymphoma	98.73	
**Unknown 106**	Lymphoma	9	1.85E+06	Lymphoma	98.49	
**Unknown 106**	Lymphoma	9	1.97E+06	Lymphoma	98.72	
**Unknown 106**	Lymphoma	13	1.67E+06	Lymphoma	98.58	
**Unknown 106**	Lymphoma	10	1.47E+06	Lymphoma	96.77	
**Unknown 106**	Lymphoma	8	9.92E+05	Lymphoma	97.08	
**Unknown 106**	Lymphoma	10	1.23E+06	Lymphoma	71.59	
**Unknown 106**	Lymphoma	10	1.88E+06	Lymphoma	94.4	
**Unknown 106**	Lymphoma	11	2.66E+06	Lymphoma	98.65	
**Unknown 106**	Lymphoma	11	2.67E+06	Lymphoma	98.46	
**Unknown 106**	Lymphoma	10	2.41E+06	Lymphoma	98.69	
**Unknown 106**	Lymphoma	11	1.58E+06	Lymphoma	99.04	
**Unknown 106**	Lymphoma	2	2.92E+05	bad data	-	
**Unknown 107**	PDX tumour	12	5.21E+06	PDX tumour	97.84	87.50
**Unknown 107**	PDX tumour	12	2.99E+06	PDX tumour	99.25	
**Unknown 107**	PDX tumour	11	2.58E+06	PDX tumour	99.17	
**Unknown 107**	PDX tumour	11	2.27E+06	PDX tumour	99.16	
**Unknown 107**	PDX tumour	5	1.86E+06	PDX tumour	99.14	
**Unknown 107**	PDX tumour	11	1.92E+06	PDX tumour	98.51	
**Unknown 107**	PDX tumour	10	1.85E+06	PDX tumour	97.93	
**Unknown 107**	PDX tumour	9	2.16E+06	Unclassifiable	-	
**Unknown 107**	PDX tumour	10	5.34E+06	Lymphoma	98.95	
**Unknown 108**	PDX tumour	12	2.25E+06	PDX tumour	99.34	100.00
**Unknown 108**	PDX tumour	10	9.46E+05	PDX tumour	99.35	
**Unknown 108**	PDX tumour	7	9.46E+05	PDX tumour	98.88	
**Unknown 108**	PDX tumour	11	1.36E+06	PDX tumour	99.38	
**Unknown 108**	PDX tumour	11	1.09E+06	PDX tumour	99.39	
**Unknown 108**	PDX tumour	11	1.45E+06	PDX tumour	99.39	
**Unknown 108**	PDX tumour	11	1.55E+06	PDX tumour	99.38	
**Unknown 108**	PDX tumour	10	1.66E+06	PDX tumour	99.35	
**Unknown 108**	PDX tumour	10	1.12E+06	PDX tumour	99.38	
**Unknown 108**	PDX tumour	11	2.93E+06	PDX tumour	99.38	
**Unknown 108**	PDX tumour	11	4.85E+05	Unclassifiable	-	
**Unknown 108**	PDX tumour	14	2.05E+06	PDX tumour	99.29	
**Unknown 108**	PDX tumour	9	2.11E+06	PDX tumour	99.22	
**Unknown 108**	PDX tumour	9	3.02E+06	PDX tumour	99.36	
**Unknown 108**	PDX tumour	12	3.41E+06	PDX tumour	99.35	
**Unknown 108**	PDX tumour	14	3.39E+06	PDX tumour	99.38	
**Unknown 108**	PDX tumour	10	4.01E+06	PDX tumour	99.36	
**Unknown 109**	Lymphoma	12	2.82E+06	Lymphoma	91.3	100.00
**Unknown 109**	Lymphoma	12	3.22E+06	Unclassifiable	-	

**Unknown 109**	Lymphoma	12	6.28E+06	Lymphoma	98.99	
**Unknown 109**	Lymphoma	8	3.07E+06	Unclassifiable	-	
**Unknown 109**	Lymphoma	9	3.96E+06	Unclassifiable	-	
**Unknown 109**	Lymphoma	9	5.11E+06	Lymphoma	99.21	
**Unknown 109**	Lymphoma	9	6.21E+06	Lymphoma	99.28	
**Unknown 109**	Lymphoma	9	2.50E+06	Unclassifiable	-	
**Unknown 109**	Lymphoma	10	4.83E+06	Lymphoma	98.7	
**Unknown 109**	Lymphoma	11	4.70E+06	Lymphoma	96.98	
**Unknown 109**	Lymphoma	12	2.96E+06	Lymphoma	98.38	
**Unknown 109**	Lymphoma	4	1.30E+06	Unclassifiable	-	
**Unknown 109**	Lymphoma	9	3.63E+06	Lymphoma	88.48	
**Unknown 109**	Lymphoma	10	4.88E+06	Lymphoma	98.85	
**Unknown 110**	PDX tumour	11	7.79E+06	PDX tumour	99.34	100.00
**Unknown 110**	PDX tumour	12	3.06E+06	PDX tumour	99.38	
**Unknown 110**	PDX tumour	13	2.22E+06	PDX tumour	99.38	
**Unknown 110**	PDX tumour	8	2.05E+06	PDX tumour	99.38	
**Unknown 110**	PDX tumour	13	1.90E+06	PDX tumour	99.39	
**Unknown 110**	PDX tumour	11	3.67E+06	PDX tumour	99.33	
**Unknown 110**	PDX tumour	11	4.64E+06	PDX tumour	99.37	
**Unknown 110**	PDX tumour	10	3.44E+06	PDX tumour	99.38	
**Unknown 110**	PDX tumour	9	2.38E+06	PDX tumour	99.37	
**Unknown 110**	PDX tumour	9	3.53E+06	PDX tumour	99.36	
**Unknown 110**	PDX tumour	10	4.45E+06	PDX tumour	99.36	
**Unknown 110**	PDX tumour	10	4.16E+06	PDX tumour	99.36	
**Unknown 111**	PDX tumour	10	3.29E+06	PDX tumour	98.57	100.00
**Unknown 111**	PDX tumour	11	4.25E+06	PDX tumour	98.37	
**Unknown 111**	PDX tumour	12	3.20E+06	PDX tumour	99.29	
**Unknown 111**	PDX tumour	10	1.67E+06	PDX tumour	99.37	
**Unknown 111**	PDX tumour	11	1.56E+06	PDX tumour	99.35	
**Unknown 111**	PDX tumour	6	1.09E+06	PDX tumour	99.32	
**Unknown 111**	PDX tumour	11	1.24E+06	PDX tumour	99.38	
**Unknown 111**	PDX tumour	10	3.02E+06	PDX tumour	99.39	
**Unknown 111**	PDX tumour	9	3.41E+06	PDX tumour	99.23	
**Unknown 111**	PDX tumour	9	2.58E+06	PDX tumour	99.31	
**Unknown 111**	PDX tumour	10	4.41E+06	PDX tumour	99.28	
**Unknown 111**	PDX tumour	9	4.08E+06	PDX tumour	99.25	
**Unknown 111**	PDX tumour	12	6.26E+06	PDX tumour	99.38	
**Unknown 111**	PDX tumour	1	5.10E+05	bad data	-	
**Unknown 112**	Lymphoma	9	9.43E+06	Lymphoma	99.33	100.00
**Unknown 112**	Lymphoma	9	9.39E+06	Lymphoma	99.38	
**Unknown 112**	Lymphoma	9	6.29E+06	Lymphoma	99.38	
**Unknown 112**	Lymphoma	10	6.87E+06	Lymphoma	99.39	

**Unknown 112**	Lymphoma	10	6.12E+06	Lymphoma	99.39	
**Unknown 112**	Lymphoma	9	6.26E+06	Lymphoma	99.35	
**Unknown 112**	Lymphoma	10	1.12E+07	Lymphoma	99.32	
**Unknown 112**	Lymphoma	9	6.78E+06	Lymphoma	99.32	
**Unknown 112**	Lymphoma	9	6.63E+06	Lymphoma	99.39	
**Unknown 112**	Lymphoma	8	6.37E+06	Lymphoma	99.38	
**Unknown 112**	Lymphoma	8	1.03E+07	Lymphoma	99.37	
**Unknown 112**	Lymphoma	9	8.95E+06	Lymphoma	99.38	
**Unknown 112**	Lymphoma	9	8.59E+06	Lymphoma	99.35	
**Unknown 112**	Lymphoma	13	8.93E+06	Lymphoma	99.38	
**Unknown 112**	Lymphoma	10	9.72E+06	Lymphoma	99.35	
**Unknown 112**	Lymphoma	10	1.09E+07	Lymphoma	99.35	
**Unknown 112**	Lymphoma	9	9.90E+06	Lymphoma	99.33	
**Unknown 112**	Lymphoma	9	6.63E+06	Lymphoma	99.37	
**Unknown 112**	Lymphoma	9	9.01E+06	Lymphoma	99.27	
**Unknown 113**	PDX tumour	8	1.13E+06	PDX tumour	99.38	100.00
**Unknown 113**	PDX tumour	7	1.24E+06	PDX tumour	99.29	
**Unknown 113**	PDX tumour	6	7.23E+05	PDX tumour	99.38	
**Unknown 113**	PDX tumour	7	1.77E+06	PDX tumour	99.37	
**Unknown 113**	PDX tumour	8	1.60E+06	PDX tumour	99.38	
**Unknown 113**	PDX tumour	10	1.60E+06	PDX tumour	99.39	
**Unknown 113**	PDX tumour	9	3.57E+06	PDX tumour	99.38	
**Unknown 113**	PDX tumour	9	2.94E+06	PDX tumour	99.37	
**Unknown 113**	PDX tumour	11	3.75E+06	PDX tumour	99.39	
**Unknown 113**	PDX tumour	9	2.45E+06	PDX tumour	99.38	
**Unknown 113**	PDX tumour	11	5.29E+06	PDX tumour	99.33	
**Unknown 113**	PDX tumour	11	3.64E+06	PDX tumour	99.38	
**Unknown 113**	PDX tumour	10	2.96E+06	PDX tumour	99.34	
**Unknown 113**	PDX tumour	8	1.05E+06	PDX tumour	99.34	
**Unknown 113**	PDX tumour	10	1.83E+06	PDX tumour	99.39	
**Unknown 113**	PDX tumour	11	3.60E+06	PDX tumour	99.36	
**Unknown 113**	PDX tumour	8	3.39E+06	PDX tumour	99.39	
**Unknown 114**	PDX tumour	10	1.22E+07	Unclassifiable	-	100.00
**Unknown 114**	PDX tumour	11	9.24E+06	PDX tumour	99.01	
**Unknown 114**	PDX tumour	10	1.10E+07	PDX tumour	99.31	
**Unknown 114**	PDX tumour	11	1.01E+07	PDX tumour	99.27	
**Unknown 114**	PDX tumour	12	1.11E+07	PDX tumour	99.14	
**Unknown 114**	PDX tumour	9	5.44E+06	PDX tumour	99.01	
**Unknown 114**	PDX tumour	10	5.54E+06	PDX tumour	99.22	
**Unknown 114**	PDX tumour	11	8.48E+06	PDX tumour	98.32	
**Unknown 114**	PDX tumour	10	7.76E+06	Unclassifiable	-	
**Unknown 114**	PDX tumour	10	8.61E+06	PDX tumour	98.37	

**Unknown 114**	PDX tumour	11	6.86E+06	PDX tumour	99.18	
**Unknown 114**	PDX tumour	11	4.95E+06	PDX tumour	98.58	
**Unknown 114**	PDX tumour	11	4.43E+06	PDX tumour	98.95	
**Unknown 114**	PDX tumour	10	7.09E+06	PDX tumour	99.35	
**Unknown 114**	PDX tumour	10	6.87E+06	Unclassifiable	-	
**Unknown 114**	PDX tumour	10	1.06E+07	PDX tumour	98.55	
**Unknown 114**	PDX tumour	11	8.75E+06	PDX tumour	98.83	
**Unknown 114**	PDX tumour	10	1.18E+07	Unclassifiable	-	
**Unknown 114**	PDX tumour	10	1.02E+07	PDX tumour	97.85	
**Unknown 114**	PDX tumour	10	1.08E+07	PDX tumour	97.92	
**Unknown 114**	PDX tumour	10	9.11E+06	PDX tumour	98.92	
**Unknown 114**	PDX tumour	10	1.09E+07	PDX tumour	98.64	
**Unknown 114**	PDX tumour	9	9.23E+06	PDX tumour	98.6	
**Unknown 114**	PDX tumour	10	1.05E+07	PDX tumour	98.94	
**Unknown 114**	PDX tumour	11	5.45E+06	PDX tumour	99.12	
**Unknown 114**	PDX tumour	10	7.20E+06	PDX tumour	98.64	
**Unknown 114**	PDX tumour	10	9.71E+06	PDX tumour	99.1	
**Unknown 115**	PDX tumour	11	3.86E+06	PDX tumour	99.32	100.00
**Unknown 115**	PDX tumour	12	4.82E+06	Unclassifiable	-	
**Unknown 115**	PDX tumour	10	4.37E+06	Unclassifiable	-	
**Unknown 115**	PDX tumour	12	4.76E+06	PDX tumour	99.19	
**Unknown 115**	PDX tumour	10	5.32E+06	PDX tumour	99.32	
**Unknown 115**	PDX tumour	9	3.41E+06	PDX tumour	99.2	
**Unknown 115**	PDX tumour	10	2.73E+06	PDX tumour	99.36	
**Unknown 115**	PDX tumour	11	4.54E+06	PDX tumour	99.31	
**Unknown 115**	PDX tumour	12	4.28E+06	PDX tumour	99.24	
**Unknown 115**	PDX tumour	12	7.88E+06	Unclassifiable	-	
**Unknown 116**	PDX tumour	12	1.38E+06	PDX tumour	99.35	100.00
**Unknown 116**	PDX tumour	9	1.95E+06	PDX tumour	99.14	
**Unknown 116**	PDX tumour	3	2.28E+05	bad data	-	
**Unknown 116**	PDX tumour	9	1.80E+06	PDX tumour	99.38	
**Unknown 116**	PDX tumour	12	2.34E+06	PDX tumour	99.28	
**Unknown 116**	PDX tumour	12	2.11E+06	PDX tumour	99.26	
**Unknown 116**	PDX tumour	9	1.72E+06	PDX tumour	99.25	
**Unknown 117**	PDX tumour	14	2.93E+06	PDX tumour	99.37	100.00
**Unknown 117**	PDX tumour	11	1.35E+06	PDX tumour	99.38	
**Unknown 117**	PDX tumour	10	1.09E+06	PDX tumour	99.38	
**Unknown 117**	PDX tumour	15	1.09E+06	PDX tumour	99.38	
**Unknown 117**	PDX tumour	10	3.49E+06	PDX tumour	99.35	
**Unknown 117**	PDX tumour	14	5.21E+06	PDX tumour	99.39	
**Unknown 117**	PDX tumour	15	1.15E+06	PDX tumour	99.36	
**Unknown 117**	PDX tumour	9	1.87E+06	PDX tumour	99.33	

**Unknown 117**	PDX tumour	12	2.90E+06	PDX tumour	99.38	
**Unknown 117**	PDX tumour	13	2.32E+06	PDX tumour	99.19	
**Unknown 117**	PDX tumour	14	3.19E+06	PDX tumour	99.38	
**Unknown 117**	PDX tumour	12	2.27E+06	PDX tumour	99.37	
**Unknown 117**	PDX tumour	6	4.94E+05	bad data	-	
**Unknown 117**	PDX tumour	15	1.79E+06	Unclassifiable	-	
**Unknown 117**	PDX tumour	12	4.25E+06	PDX tumour	99.37	
**Unknown 117**	PDX tumour	11	3.53E+06	PDX tumour	99.38	
**Unknown 117**	PDX tumour	12	9.81E+05	bad data	-	
**Unknown 117**	PDX tumour	13	1.16E+06	PDX tumour	99.39	
**Unknown 117**	PDX tumour	12	7.53E+06	PDX tumour	99.35	
**Unknown 117**	PDX tumour	10	5.32E+06	PDX tumour	99.14	
**Unknown 118**	PDX tumour	10	3.64E+06	PDX tumour	99.38	100.00
**Unknown 118**	PDX tumour	10	2.58E+06	PDX tumour	99.27	
**Unknown 118**	PDX tumour	11	4.21E+06	PDX tumour	99.34	
**Unknown 118**	PDX tumour	10	2.69E+06	PDX tumour	99.37	
**Unknown 118**	PDX tumour	8	4.82E+05	PDX tumour	99.38	
**Unknown 118**	PDX tumour	9	2.42E+06	PDX tumour	99.38	
**Unknown 118**	PDX tumour	11	2.18E+06	PDX tumour	99.38	
**Unknown 118**	PDX tumour	10	3.81E+06	PDX tumour	99.26	
**Unknown 119**	PDX tumour	11	4.84E+06	PDX tumour	99.37	100.00
**Unknown 119**	PDX tumour	14	4.67E+06	PDX tumour	99.39	
**Unknown 119**	PDX tumour	12	4.96E+06	PDX tumour	99.38	
**Unknown 119**	PDX tumour	10	4.22E+06	PDX tumour	99.32	
**Unknown 119**	PDX tumour	11	3.70E+06	PDX tumour	99.39	
**Unknown 119**	PDX tumour	10	3.43E+06	PDX tumour	99.37	
**Unknown 119**	PDX tumour	13	5.23E+05	PDX tumour	98.73	
**Unknown 119**	PDX tumour	11	5.48E+06	PDX tumour	99.38	
**Unknown 119**	PDX tumour	14	1.31E+06	PDX tumour	99.36	
**Unknown 119**	PDX tumour	8	1.30E+06	PDX tumour	99.29	
**Unknown 119**	PDX tumour	13	3.08E+06	PDX tumour	99.38	
**Unknown 120**	PDX tumour	12	2.90E+06	PDX tumour	99.29	100.00
**Unknown 120**	PDX tumour	10	2.15E+06	PDX tumour	99.24	
**Unknown 120**	PDX tumour	8	1.76E+06	PDX tumour	99.38	
**Unknown 120**	PDX tumour	12	2.46E+06	PDX tumour	99.28	
**Unknown 121**	PDX tumour	9	5.30E+06	PDX tumour	97.67	100.00
**Unknown 121**	PDX tumour	8	1.80E+06	PDX tumour	99.2	
**Unknown 121**	PDX tumour	7	1.23E+06	PDX tumour	99.23	
**Unknown 121**	PDX tumour	6	9.24E+05	PDX tumour	98.62	
**Unknown 121**	PDX tumour	8	4.91E+06	PDX tumour	98.74	
**Unknown 121**	PDX tumour	9	5.29E+06	PDX tumour	98.99	
**Unknown 121**	PDX tumour	9	4.48E+06	Unclassifiable	-	

**Unknown 121**	PDX tumour	9	4.97E+06	Unclassifiable	-	
**Unknown 121**	PDX tumour	1	1.41E+05	bad data	-	
**Unknown 122**	Lymphoma	10	6.05E+06	Lymphoma	99.18	100.00
**Unknown 122**	Lymphoma	9	4.95E+06	Lymphoma	99.08	
**Unknown 122**	Lymphoma	11	6.79E+06	Lymphoma	99.06	
**Unknown 122**	Lymphoma	10	6.49E+06	Unclassifiable	-	
**Unknown 122**	Lymphoma	9	9.27E+06	Lymphoma	99.16	
**Unknown 122**	Lymphoma	10	8.45E+06	Lymphoma	99.14	
**Unknown 122**	Lymphoma	11	8.51E+06	Lymphoma	99.24	
**Unknown 122**	Lymphoma	3	3.65E+06	bad data	-	
**Unknown 122**	Lymphoma	11	1.19E+07	Lymphoma	99.18	
**Unknown 122**	Lymphoma	10	7.84E+06	Lymphoma	99.19	
**Unknown 122**	Lymphoma	10	5.27E+06	Lymphoma	99.14	
**Unknown 122**	Lymphoma	7	1.75E+06	Lymphoma	99.38	
**Unknown 122**	Lymphoma	8	4.39E+06	Lymphoma	99.34	
**Unknown 122**	Lymphoma	10	6.45E+06	Lymphoma	99.36	
**Unknown 122**	Lymphoma	10	1.59E+07	Lymphoma	99.28	
**Unknown 123**	PDX tumour	9	1.48E+06	PDX tumour	99.38	100.00
**Unknown 123**	PDX tumour	11	1.51E+06	PDX tumour	99.27	
**Unknown 123**	PDX tumour	9	1.03E+06	PDX tumour	99.34	
**Unknown 123**	PDX tumour	12	1.48E+06	PDX tumour	99.35	
**Unknown 123**	PDX tumour	10	2.37E+06	PDX tumour	99.38	
**Unknown 123**	PDX tumour	9	1.19E+06	PDX tumour	99.08	
**Unknown 123**	PDX tumour	9	2.16E+06	PDX tumour	99.38	
**Unknown 123**	PDX tumour	11	2.61E+06	PDX tumour	99.38	
**Unknown 123**	PDX tumour	10	3.13E+06	PDX tumour	99.13	
**Unknown 123**	PDX tumour	9	2.34E+06	PDX tumour	99.39	
**Unknown 123**	PDX tumour	10	2.22E+06	PDX tumour	99.39	
**Unknown 123**	PDX tumour	9	4.34E+06	PDX tumour	99.38	
**Unknown 123**	PDX tumour	9	4.45E+06	PDX tumour	99.31	
**Unknown 123**	PDX tumour	9	4.91E+06	PDX tumour	99.38	
**Unknown 123**	PDX tumour	9	4.45E+06	PDX tumour	99.36	
**Unknown 123**	PDX tumour	8	3.22E+06	PDX tumour	99.23	
**Unknown 123**	PDX tumour	8	4.53E+06	PDX tumour	99.34	
**Unknown 123**	PDX tumour	9	5.14E+06	PDX tumour	99.39	
**Unknown 123**	PDX tumour	9	3.35E+06	PDX tumour	99.38	
**Unknown 123**	PDX tumour	8	3.53E+06	PDX tumour	99.38	
**Unknown 123**	PDX tumour	7	3.16E+06	PDX tumour	99.32	
**Unknown 123**	PDX tumour	8	3.58E+06	PDX tumour	99.38	
**Unknown 123**	PDX tumour	10	7.47E+06	PDX tumour	99.39	
**Unknown 124**	PDX tumour	13	3.71E+06	PDX tumour	99.38	100.00
**Unknown 124**	PDX tumour	13	2.09E+06	PDX tumour	99.37	

**Unknown 124**	PDX tumour	9	1.01E+06	PDX tumour	99.26	
**Unknown 124**	PDX tumour	13	1.21E+06	PDX tumour	99.3	
**Unknown 124**	PDX tumour	10	2.74E+06	PDX tumour	99.25	
**Unknown 124**	PDX tumour	11	2.79E+06	PDX tumour	99.38	
**Unknown 124**	PDX tumour	13	3.42E+06	PDX tumour	99.37	
**Unknown 124**	PDX tumour	10	4.23E+06	PDX tumour	99.35	
**Unknown 125**	PDX tumour	11	5.85E+06	PDX tumour	99.28	100.00
**Unknown 125**	PDX tumour	9	5.43E+06	PDX tumour	99.35	
**Unknown 125**	PDX tumour	6	3.81E+06	PDX tumour	99.33	
**Unknown 125**	PDX tumour	8	4.38E+06	PDX tumour	99.37	
**Unknown 125**	PDX tumour	12	4.90E+06	PDX tumour	99.36	
**Unknown 125**	PDX tumour	7	3.39E+06	PDX tumour	99.27	
**Unknown 125**	PDX tumour	9	5.23E+06	PDX tumour	99.28	
**Unknown 125**	PDX tumour	8	5.70E+06	PDX tumour	99.31	
**Unknown 126**	PDX tumour	12	5.41E+05	Unclassifiable	-	100.00
**Unknown 126**	PDX tumour	11	3.51E+05	PDX tumour	99.3	
**Unknown 126**	PDX tumour	12	6.97E+05	PDX tumour	99.24	
**Unknown 126**	PDX tumour	12	1.83E+06	PDX tumour	99.24	
**Unknown 126**	PDX tumour	11	6.41E+05	PDX tumour	98.59	
**Unknown 126**	PDX tumour	11	1.53E+06	PDX tumour	98.74	
**Unknown 126**	PDX tumour	12	8.75E+05	PDX tumour	99.22	
**Unknown 127**	PDX tumour	8	6.93E+06	PDX tumour	99.18	100.00
**Unknown 127**	PDX tumour	9	4.56E+06	PDX tumour	99.38	
**Unknown 127**	PDX tumour	10	4.72E+06	PDX tumour	99.35	
**Unknown 127**	PDX tumour	9	7.31E+06	PDX tumour	99.37	
**Unknown 127**	PDX tumour	11	8.83E+06	PDX tumour	99.35	
**Unknown 127**	PDX tumour	10	9.04E+06	PDX tumour	99.36	
**Unknown 127**	PDX tumour	9	8.06E+06	PDX tumour	99.34	
**Unknown 127**	PDX tumour	10	8.42E+06	PDX tumour	99.35	
**Unknown 127**	PDX tumour	10	6.66E+06	PDX tumour	99.36	
**Unknown 127**	PDX tumour	10	7.04E+06	PDX tumour	99.28	
**Unknown 127**	PDX tumour	10	6.74E+06	PDX tumour	99.33	
**Unknown 127**	PDX tumour	11	3.16E+06	PDX tumour	99.31	
**Unknown 127**	PDX tumour	10	2.73E+06	PDX tumour	99.35	
**Unknown 127**	PDX tumour	10	2.33E+06	PDX tumour	99.28	
**Unknown 127**	PDX tumour	9	7.83E+06	PDX tumour	99.32	
**Unknown 127**	PDX tumour	10	3.03E+06	PDX tumour	99.31	
**Unknown 127**	PDX tumour	11	4.11E+06	PDX tumour	99.29	
**Unknown 128**	PDX tumour	11	3.26E+06	PDX tumour	99.34	100.00
**Unknown 128**	PDX tumour	11	2.86E+06	PDX tumour	99.38	
**Unknown 128**	PDX tumour	12	3.04E+06	PDX tumour	99.39	
**Unknown 128**	PDX tumour	14	2.74E+06	PDX tumour	99.35	

**Unknown 128**	PDX tumour	14	1.21E+06	PDX tumour	99.39	
**Unknown 128**	PDX tumour	8	1.13E+06	PDX tumour	99.34	
**Unknown 128**	PDX tumour	10	2.52E+06	PDX tumour	99.32	
**Unknown 128**	PDX tumour	10	2.29E+06	PDX tumour	99.33	
**Unknown 128**	PDX tumour	10	2.68E+06	PDX tumour	99.32	
**Unknown 128**	PDX tumour	11	2.50E+06	PDX tumour	99.35	
**Unknown 128**	PDX tumour	14	1.84E+06	PDX tumour	99.31	
**Unknown 128**	PDX tumour	14	1.46E+06	PDX tumour	99.38	
**Unknown 128**	PDX tumour	13	2.84E+06	PDX tumour	99.38	
**Unknown 128**	PDX tumour	11	4.46E+06	PDX tumour	99.29	
**Unknown 128**	PDX tumour	13	4.12E+06	PDX tumour	99.36	
**Unknown 128**	PDX tumour	2	2.76E+05	bad data	-	
**Unknown 129**	PDX tumour	11	9.15E+05	PDX tumour	99.39	100.00
**Unknown 129**	PDX tumour	12	1.44E+06	PDX tumour	99.37	
**Unknown 129**	PDX tumour	10	4.68E+06	PDX tumour	99.39	
**Unknown 129**	PDX tumour	10	4.04E+06	PDX tumour	99.38	
**Unknown 129**	PDX tumour	10	2.09E+06	PDX tumour	99.38	
**Unknown 129**	PDX tumour	11	1.74E+06	PDX tumour	99.32	
**Unknown 129**	PDX tumour	7	5.88E+05	PDX tumour	99.38	
**Unknown 129**	PDX tumour	8	3.88E+06	PDX tumour	99.39	
**Unknown 129**	PDX tumour	9	3.93E+06	PDX tumour	99.37	
**Unknown 129**	PDX tumour	10	2.31E+06	PDX tumour	99.38	
**Unknown 129**	PDX tumour	11	2.79E+06	PDX tumour	99.38	
**Unknown 129**	PDX tumour	10	7.11E+06	PDX tumour	99.38	
**Unknown 129**	PDX tumour	9	8.19E+06	PDX tumour	99.28	
**Unknown 130**	Lymphoma	12	1.46E+07	Lymphoma	99.11	100.00
**Unknown 130**	Lymphoma	13	1.26E+07	Lymphoma	99.13	
**Unknown 130**	Lymphoma	11	9.69E+06	Lymphoma	99.15	
**Unknown 130**	Lymphoma	12	9.65E+06	Lymphoma	99.19	
**Unknown 130**	Lymphoma	12	1.29E+07	Lymphoma	99.1	
**Unknown 130**	Lymphoma	13	1.28E+07	Unclassifiable	-	
**Unknown 130**	Lymphoma	12	1.43E+07	Unclassifiable	-	
**Unknown 130**	Lymphoma	11	4.80E+06	Lymphoma	99.21	
**Unknown 130**	Lymphoma	13	9.00E+06	Unclassifiable	-	
**Unknown 130**	Lymphoma	14	1.07E+07	Unclassifiable	-	
**Unknown 130**	Lymphoma	15	8.87E+06	Unclassifiable	-	
**Unknown 131**	Lymphoma	11	1.46E+06	Lymphoma	99.39	100.00
**Unknown 131**	Lymphoma	12	1.68E+06	Lymphoma	95.91	
**Unknown 131**	Lymphoma	12	1.97E+06	Lymphoma	99.14	
**Unknown 131**	Lymphoma	13	1.24E+06	Lymphoma	99.13	
**Unknown 131**	Lymphoma	9	8.89E+05	Lymphoma	98.99	
**Unknown 131**	Lymphoma	10	1.58E+06	Lymphoma	99.25	

**Unknown 131**	Lymphoma	13	1.31E+06	Lymphoma	99.1	
**Unknown 131**	Lymphoma	13	1.23E+06	Lymphoma	98.89	
**Unknown 131**	Lymphoma	10	6.52E+05	Unclassifiable	-	
**Unknown 131**	Lymphoma	10	5.65E+05	Unclassifiable	-	
**Unknown 131**	Lymphoma	12	1.16E+06	Lymphoma	98.7	
**Unknown 132**	PDX tumour	10	5.74E+06	PDX tumour	99.13	100.00
**Unknown 132**	PDX tumour	9	1.61E+06	PDX tumour	99.25	
**Unknown 132**	PDX tumour	10	3.96E+06	PDX tumour	99.32	
**Unknown 132**	PDX tumour	11	2.49E+06	PDX tumour	99.37	
**Unknown 132**	PDX tumour	13	5.40E+06	PDX tumour	99.28	
**Unknown 132**	PDX tumour	10	3.62E+06	PDX tumour	99.35	
**Unknown 132**	PDX tumour	13	1.83E+06	PDX tumour	99.35	
**Unknown 132**	PDX tumour	10	8.50E+06	PDX tumour	99.17	
**Unknown 132**	PDX tumour	12	3.70E+06	PDX tumour	99.31	
**Unknown 132**	PDX tumour	9	2.99E+06	PDX tumour	99.23	
**Unknown 132**	PDX tumour	10	5.72E+06	PDX tumour	99.31	
**Unknown 132**	PDX tumour	10	3.15E+06	PDX tumour	99.37	
**Unknown 132**	PDX tumour	9	3.53E+06	PDX tumour	99.37	
**Unknown 132**	PDX tumour	9	6.00E+06	PDX tumour	99.25	
**Unknown 132**	PDX tumour	10	5.27E+06	PDX tumour	99.24	
**Unknown 132**	PDX tumour	9	6.37E+06	Unclassifiable	-	
**Unknown 132**	PDX tumour	13	6.39E+06	PDX tumour	99.16	
**Unknown 132**	PDX tumour	13	5.85E+06	PDX tumour	99.18	
**Unknown 132**	PDX tumour	8	5.73E+06	PDX tumour	99.3	
**Unknown 132**	PDX tumour	10	3.80E+06	PDX tumour	99.33	
**Unknown 132**	PDX tumour	8	1.82E+06	PDX tumour	99.38	
**Unknown 132**	PDX tumour	9	4.78E+06	PDX tumour	99.39	
**Unknown 132**	PDX tumour	13	6.80E+06	PDX tumour	99.37	
**Unknown 132**	PDX tumour	7	3.66E+06	PDX tumour	99.38	
**Unknown 132**	PDX tumour	8	4.41E+06	PDX tumour	99.38	
**Unknown 133**	PDX tumour	10	4.30E+06	PDX tumour	99.38	100.00
**Unknown 133**	PDX tumour	9	2.87E+06	PDX tumour	99.35	
**Unknown 133**	PDX tumour	10	2.07E+06	PDX tumour	99.27	
**Unknown 133**	PDX tumour	8	3.64E+06	PDX tumour	99.38	
**Unknown 133**	PDX tumour	10	1.97E+06	PDX tumour	99.39	
**Unknown 134**	PDX tumour	10	3.29E+06	PDX tumour	99.38	100.00
**Unknown 134**	PDX tumour	9	3.84E+06	PDX tumour	99.38	
**Unknown 134**	PDX tumour	8	1.76E+06	PDX tumour	99.38	
**Unknown 134**	PDX tumour	12	3.90E+06	PDX tumour	99.3	
**Unknown 134**	PDX tumour	12	2.92E+06	PDX tumour	99.39	
**Unknown 134**	PDX tumour	8	2.53E+06	PDX tumour	99.37	
**Unknown 134**	PDX tumour	10	2.93E+06	PDX tumour	99.37	

**Unknown 134**	PDX tumour	11	3.21E+06	PDX tumour	99.39	
**Unknown 134**	PDX tumour	10	2.17E+06	PDX tumour	99.37	
**Unknown 134**	PDX tumour	11	3.09E+06	PDX tumour	99.38	
**Unknown 134**	PDX tumour	10	1.98E+06	PDX tumour	99.38	
**Unknown 134**	PDX tumour	10	2.61E+06	PDX tumour	99.33	
**Unknown 134**	PDX tumour	9	3.22E+06	PDX tumour	99.39	
**Unknown 135**	PDX tumour	9	7.39E+06	PDX tumour	99.38	100.00
**Unknown 135**	PDX tumour	8	8.44E+06	PDX tumour	99.37	
**Unknown 135**	PDX tumour	9	4.36E+06	PDX tumour	99.38	
**Unknown 135**	PDX tumour	9	5.54E+06	PDX tumour	99.36	
**Unknown 135**	PDX tumour	10	5.86E+06	PDX tumour	99.39	
**Unknown 135**	PDX tumour	9	7.40E+06	PDX tumour	99.37	
**Unknown 135**	PDX tumour	10	4.79E+06	PDX tumour	99.34	
**Unknown 136**	PDX tumour	10	3.62E+06	PDX tumour	97.6	100.00
**Unknown 136**	PDX tumour	11	2.69E+06	Unclassifiable	-	
**Unknown 136**	PDX tumour	12	2.09E+06	PDX tumour	99.19	
**Unknown 136**	PDX tumour	12	1.69E+06	Unclassifiable	-	
**Unknown 136**	PDX tumour	15	2.24E+06	PDX tumour	98.31	
**Unknown 137**	Lymphoma	15	6.41E+06	Lymphoma	99.36	100.00
**Unknown 137**	Lymphoma	11	7.76E+06	Lymphoma	99.28	
**Unknown 137**	Lymphoma	11	8.11E+06	Lymphoma	99.29	
**Unknown 137**	Lymphoma	12	7.93E+06	Lymphoma	99.37	
**Unknown 137**	Lymphoma	11	3.21E+06	Lymphoma	99.34	
**Unknown 137**	Lymphoma	14	2.09E+06	Lymphoma	99.23	
**Unknown 137**	Lymphoma	12	7.23E+06	Lymphoma	99.39	
**Unknown 137**	Lymphoma	11	5.82E+06	Lymphoma	99.33	
**Unknown 137**	Lymphoma	12	5.74E+06	Lymphoma	99.18	
**Unknown 137**	Lymphoma	12	5.39E+06	Lymphoma	99.38	
**Unknown 137**	Lymphoma	12	6.49E+06	Lymphoma	99.38	
**Unknown 137**	Lymphoma	11	7.07E+06	Lymphoma	99.39	
**Unknown 137**	Lymphoma	11	6.48E+06	Lymphoma	99.38	
**Unknown 137**	Lymphoma	11	4.75E+06	Unclassifiable	-	
**Unknown 137**	Lymphoma	12	4.12E+06	Unclassifiable	-	
**Unknown 137**	Lymphoma	12	8.21E+06	Lymphoma	99.38	
**Unknown 137**	Lymphoma	11	7.24E+06	Lymphoma	99.25	
**Unknown 137**	Lymphoma	12	1.00E+07	Lymphoma	99.38	
**Unknown 137**	Lymphoma	11	6.23E+06	Lymphoma	99.38	
**Unknown 137**	Lymphoma	11	4.52E+06	Lymphoma	99.36	
**Unknown 137**	Lymphoma	13	5.98E+06	Lymphoma	98.58	
**Unknown 137**	Lymphoma	10	8.59E+06	Lymphoma	99.3	
**Unknown 138**	PDX tumour	11	4.15E+06	Unclassifiable	-	100.00
**Unknown 138**	PDX tumour	9	2.86E+06	PDX tumour	99.18	

**Unknown 138**	PDX tumour	10	2.92E+06	PDX tumour	99.26	
**Unknown 138**	PDX tumour	9	3.41E+06	PDX tumour	99.38	
**Unknown 138**	PDX tumour	8	3.33E+06	PDX tumour	99.29	
**Unknown 138**	PDX tumour	10	4.08E+06	PDX tumour	99.35	
**Unknown 138**	PDX tumour	11	2.38E+06	PDX tumour	99.36	
**Unknown 138**	PDX tumour	12	4.07E+06	PDX tumour	98.91	
**Unknown 138**	PDX tumour	10	1.60E+06	PDX tumour	99.31	
**Unknown 138**	PDX tumour	9	6.04E+05	Unclassifiable	-	
**Unknown 138**	PDX tumour	10	1.50E+06	PDX tumour	98.85	
**Unknown 138**	PDX tumour	12	2.92E+06	PDX tumour	98.29	
**Unknown 138**	PDX tumour	10	1.57E+06	PDX tumour	99.28	
**Unknown 138**	PDX tumour	10	2.29E+06	PDX tumour	99.37	
**Unknown 138**	PDX tumour	10	2.65E+06	PDX tumour	98.99	
**Unknown 138**	PDX tumour	10	3.06E+06	PDX tumour	99.34	
**Unknown 138**	PDX tumour	9	1.92E+06	PDX tumour	98.96	
**Unknown 138**	PDX tumour	10	4.11E+06	PDX tumour	99.38	
**Unknown 139**	PDX tumour	11	3.67E+06	PDX tumour	99.26	100.00
**Unknown 139**	PDX tumour	11	3.32E+06	PDX tumour	99.38	
**Unknown 139**	PDX tumour	14	3.39E+06	PDX tumour	99.31	
**Unknown 139**	PDX tumour	14	2.86E+06	PDX tumour	99.17	
**Unknown 139**	PDX tumour	11	1.37E+06	PDX tumour	99.33	
**Unknown 139**	PDX tumour	12	2.64E+06	PDX tumour	99.35	
**Unknown 140**	PDX tumour	9	4.42E+06	PDX tumour	99.33	100.00
**Unknown 140**	PDX tumour	10	7.01E+06	PDX tumour	99.38	
**Unknown 140**	PDX tumour	10	6.77E+06	PDX tumour	99.28	
**Unknown 140**	PDX tumour	10	5.89E+06	PDX tumour	99.39	
**Unknown 140**	PDX tumour	15	7.37E+06	PDX tumour	99.31	
**Unknown 140**	PDX tumour	10	6.65E+06	PDX tumour	99.05	
**Unknown 140**	PDX tumour	10	6.55E+06	PDX tumour	99.31	
**Unknown 140**	PDX tumour	12	1.11E+07	PDX tumour	99.19	
**Unknown 140**	PDX tumour	10	4.41E+06	PDX tumour	98.9	
**Unknown 140**	PDX tumour	10	7.26E+06	PDX tumour	98.63	
**Unknown 140**	PDX tumour	10	8.13E+06	PDX tumour	99.28	
**Unknown 140**	PDX tumour	10	3.20E+06	PDX tumour	98.74	
**Unknown 140**	PDX tumour	10	7.72E+06	PDX tumour	98.48	
**Unknown 140**	PDX tumour	10	3.11E+06	PDX tumour	99.2	
**Unknown 140**	PDX tumour	10	1.86E+06	PDX tumour	99.21	
**Unknown 140**	PDX tumour	11	6.98E+06	PDX tumour	98.64	
**Unknown 140**	PDX tumour	11	5.01E+06	PDX tumour	98.03	
**Unknown 140**	PDX tumour	11	6.15E+06	PDX tumour	98.36	
**Unknown 140**	PDX tumour	11	9.17E+06	PDX tumour	99.04	
**Unknown 140**	PDX tumour	11	9.59E+06	PDX tumour	98.4	

**Unknown 140**	PDX tumour	11	1.15E+07	Unclassifiable	-	
**Unknown 140**	PDX tumour	12	1.23E+07	PDX tumour	99.29	
**Unknown 140**	PDX tumour	12	9.60E+06	PDX tumour	99.37	
**Unknown 140**	PDX tumour	13	8.37E+06	PDX tumour	99.39	
**Unknown 140**	PDX tumour	10	1.59E+07	PDX tumour	99.28	
**Unknown 141**	Lymphoma	11	5.12E+06	PDX tumour	98.94	75.00
**Unknown 141**	Lymphoma	10	6.61E+06	Lymphoma	97.71	
**Unknown 141**	Lymphoma	10	1.17E+07	Lymphoma	<u>84.92</u>	
**Unknown 141**	Lymphoma	14	6.58E+06	Unclassifiable	-	
**Unknown 141**	Lymphoma	7	7.64E+06	Unclassifiable	-	
**Unknown 141**	Lymphoma	11	1.01E+07	Lymphoma	<u>92.94</u>	
**Unknown 142**	Lymphoma	10	3.15E+06	Lymphoma	<u>92.75</u>	100.00
**Unknown 142**	Lymphoma	12	2.54E+06	Lymphoma	99.14	
**Unknown 142**	Lymphoma	12	5.02E+06	Lymphoma	99.29	
**Unknown 142**	Lymphoma	12	3.78E+06	Lymphoma	99.13	
**Unknown 142**	Lymphoma	13	5.22E+06	Lymphoma	99.35	
**Unknown 142**	Lymphoma	12	3.89E+06	Lymphoma	99.37	
**Unknown 142**	Lymphoma	11	3.12E+06	Lymphoma	99.38	
**Unknown 142**	Lymphoma	12	2.78E+06	Lymphoma	99.08	
**Unknown 142**	Lymphoma	14	3.08E+06	Lymphoma	99.3	
**Unknown 142**	Lymphoma	14	2.46E+06	Lymphoma	99.17	
**Unknown 142**	Lymphoma	14	2.21E+06	Lymphoma	97.82	
**Unknown 142**	Lymphoma	6	9.50E+05	Lymphoma	96.52	
**Unknown 142**	Lymphoma	11	3.53E+06	Lymphoma	99.1	
**Unknown 142**	Lymphoma	10	3.22E+06	Lymphoma	98.91	
**Unknown 142**	Lymphoma	11	2.56E+06	Lymphoma	99.32	
**Unknown 142**	Lymphoma	10	5.33E+06	Lymphoma	99.31	
**Unknown 142**	Lymphoma	11	4.07E+06	Lymphoma	99.35	
**Unknown 142**	Lymphoma	11	5.37E+06	Lymphoma	99.39	
**Unknown 142**	Lymphoma	10	4.88E+06	Lymphoma	99.37	
**Unknown 142**	Lymphoma	11	3.40E+06	Lymphoma	96.44	
**Unknown 142**	Lymphoma	6	1.14E+06	Lymphoma	99.06	
**Unknown 142**	Lymphoma	11	2.10E+06	Lymphoma	99.39	
**Unknown 142**	Lymphoma	9	2.34E+06	Lymphoma	99.38	
**Unknown 142**	Lymphoma	11	4.03E+06	Lymphoma	99.34	
**Unknown 142**	Lymphoma	10	4.49E+06	Lymphoma	99.35	
**Unknown 143**	PDX tumour	12	2.06E+06	PDX tumour	98.71	100.00
**Unknown 143**	PDX tumour	12	1.45E+06	PDX tumour	99.38	
**Unknown 143**	PDX tumour	11	1.72E+06	PDX tumour	99.38	
**Unknown 143**	PDX tumour	10	1.29E+06	PDX tumour	99.38	
**Unknown 143**	PDX tumour	9	2.10E+06	PDX tumour	99.37	
**Unknown 143**	PDX tumour	11	1.73E+06	PDX tumour	99.39	

**Unknown 143**	PDX tumour	10	1.17E+06	PDX tumour	99.38	
**Unknown 143**	PDX tumour	11	1.35E+06	PDX tumour	99.37	
**Unknown 143**	PDX tumour	14	1.53E+06	PDX tumour	99.34	
**Unknown 143**	PDX tumour	14	1.60E+06	PDX tumour	99.33	
**Unknown 143**	PDX tumour	14	1.63E+06	PDX tumour	99.29	
**Unknown 143**	PDX tumour	11	1.85E+06	PDX tumour	99.38	
**Unknown 143**	PDX tumour	10	1.57E+06	PDX tumour	99.39	
**Unknown 143**	PDX tumour	8	1.05E+06	PDX tumour	99.39	
**Unknown 143**	PDX tumour	14	1.23E+06	PDX tumour	99.35	
**Unknown 144**	PDX tumour	10	2.52E+06	PDX tumour	99.38	100.00
**Unknown 144**	PDX tumour	11	1.84E+06	PDX tumour	99.27	
**Unknown 144**	PDX tumour	13	2.51E+06	PDX tumour	99.37	
**Unknown 144**	PDX tumour	12	1.22E+06	PDX tumour	99.36	
**Unknown 144**	PDX tumour	10	1.37E+06	PDX tumour	99.36	
**Unknown 144**	PDX tumour	14	1.07E+06	PDX tumour	99.38	
**Unknown 144**	PDX tumour	8	1.27E+06	PDX tumour	99.36	
**Unknown 144**	PDX tumour	14	2.98E+06	PDX tumour	99.23	
**Unknown 144**	PDX tumour	14	1.59E+06	Unclassifiable	-	
**Unknown 144**	PDX tumour	14	1.27E+06	PDX tumour	99.31	
**Unknown 144**	PDX tumour	14	2.99E+06	PDX tumour	99.23	
**Unknown 144**	PDX tumour	14	3.62E+06	PDX tumour	99.3	
**Unknown 144**	PDX tumour	12	2.67E+06	PDX tumour	99.28	
**Unknown 144**	PDX tumour	11	2.47E+06	PDX tumour	99.33	
**Unknown 144**	PDX tumour	13	3.54E+06	PDX tumour	99.25	
**Unknown 144**	PDX tumour	14	4.85E+05	PDX tumour	99.29	
**Unknown 144**	PDX tumour	11	3.60E+06	PDX tumour	99.3	
**Unknown 144**	PDX tumour	10	2.79E+06	PDX tumour	99.2	
**Unknown 144**	PDX tumour	8	3.25E+06	PDX tumour	99.2	
**Unknown 145**	Lymphoma	7	4.70E+06	Lymphoma	83.26	100.00
**Unknown 145**	Lymphoma	11	5.56E+06	Lymphoma	98.05	
**Unknown 145**	Lymphoma	10	5.49E+06	Lymphoma	98.05	
**Unknown 145**	Lymphoma	12	5.81E+06	Lymphoma	81.91	
**Unknown 145**	Lymphoma	10	2.75E+06	Unclassifiable	-	
**Unknown 145**	Lymphoma	11	2.96E+06	Unclassifiable	-	
**Unknown 145**	Lymphoma	11	1.60E+06	Lymphoma	55.02	
**Unknown 145**	Lymphoma	11	1.20E+07	Lymphoma	98.93	
**Unknown 145**	Lymphoma	13	1.46E+07	Lymphoma	98.71	
**Unknown 145**	Lymphoma	11	8.80E+06	Lymphoma	99.04	
**Unknown 145**	Lymphoma	11	7.96E+06	Lymphoma	98.8	
**Unknown 145**	Lymphoma	12	1.52E+07	Lymphoma	98.94	
**Unknown 145**	Lymphoma	13	1.32E+07	Lymphoma	98.64	
**Unknown 145**	Lymphoma	11	1.29E+07	Lymphoma	98.43	

**Unknown 145**	Lymphoma	11	1.81E+07	Lymphoma	99.34	
**Unknown 145**	Lymphoma	11	9.25E+06	Lymphoma	99.13	
**Unknown 145**	Lymphoma	11	1.09E+07	Lymphoma	99.22	
**Unknown 145**	Lymphoma	11	6.82E+06	Lymphoma	92.4	
**Unknown 145**	Lymphoma	11	4.94E+06	Lymphoma	96.59	
**Unknown 145**	Lymphoma	11	6.66E+06	Lymphoma	98.72	
**Unknown 145**	Lymphoma	12	3.62E+06	Lymphoma	93.44	
**Unknown 146**	PDX tumour	12	2.06E+06	PDX tumour	98.03	93.75
**Unknown 146**	PDX tumour	9	1.01E+06	PDX tumour	97.94	
**Unknown 146**	PDX tumour	12	1.83E+06	Lymphoma	83.32	
**Unknown 146**	PDX tumour	14	3.35E+06	PDX tumour	97.59	
**Unknown 146**	PDX tumour	12	3.45E+06	PDX tumour	98.78	
**Unknown 146**	PDX tumour	12	2.75E+06	PDX tumour	98	
**Unknown 146**	PDX tumour	11	1.92E+06	PDX tumour	98.03	
**Unknown 146**	PDX tumour	11	2.55E+06	PDX tumour	98.59	
**Unknown 146**	PDX tumour	12	2.80E+06	PDX tumour	98.65	
**Unknown 146**	PDX tumour	12	1.89E+06	PDX tumour	99.01	
**Unknown 146**	PDX tumour	12	3.54E+06	PDX tumour	99.2	
**Unknown 146**	PDX tumour	12	4.65E+06	Unclassifiable	-	
**Unknown 146**	PDX tumour	13	3.45E+06	PDX tumour	98.29	
**Unknown 146**	PDX tumour	13	2.84E+06	PDX tumour	98.69	
**Unknown 146**	PDX tumour	11	3.73E+06	PDX tumour	98.68	
**Unknown 146**	PDX tumour	11	2.15E+06	Unclassifiable	-	
**Unknown 146**	PDX tumour	11	1.78E+06	PDX tumour	99.05	
**Unknown 146**	PDX tumour	10	3.89E+06	PDX tumour	98.6	
**Unknown 146**	PDX tumour	11	3.81E+06	Unclassifiable	-	
**Unknown 147**	PDX tumour	11	1.16E+06	PDX tumour	99.31	100.00
**Unknown 147**	PDX tumour	10	6.06E+05	PDX tumour	99.38	
**Unknown 147**	PDX tumour	10	2.24E+06	PDX tumour	99.2	
**Unknown 147**	PDX tumour	9	9.49E+05	PDX tumour	99.39	
**Unknown 147**	PDX tumour	12	1.83E+06	PDX tumour	99.37	
**Unknown 147**	PDX tumour	10	1.77E+06	PDX tumour	99.39	
**Unknown 147**	PDX tumour	4	5.36E+05	PDX tumour	99.14	
**Unknown 147**	PDX tumour	12	2.25E+06	PDX tumour	99.28	
**Unknown 147**	PDX tumour	9	1.52E+06	PDX tumour	99.36	
**Unknown 147**	PDX tumour	8	2.48E+06	PDX tumour	99.31	
**Unknown 147**	PDX tumour	10	1.18E+06	PDX tumour	99.34	
**Unknown 147**	PDX tumour	8	1.21E+06	PDX tumour	99.36	
**Unknown 147**	PDX tumour	10	4.05E+06	PDX tumour	99.1	
**Unknown 147**	PDX tumour	11	4.20E+06	PDX tumour	98.61	
**Unknown 147**	PDX tumour	12	5.00E+06	PDX tumour	98.36	
**Unknown 148**	PDX tumour	12	6.83E+06	PDX tumour	99.27	100.00

**Unknown 148**	PDX tumour	13	2.75E+06	PDX tumour	99.26
**Unknown 148**	PDX tumour	12	2.53E+06	PDX tumour	99.27
**Unknown 148**	PDX tumour	13	2.57E+06	PDX tumour	99.34
**Unknown 148**	PDX tumour	12	1.90E+06	PDX tumour	99.38
**Unknown 148**	PDX tumour	11	3.34E+06	PDX tumour	99.27
**Unknown 148**	PDX tumour	10	1.14E+06	PDX tumour	99.36
**Unknown 148**	PDX tumour	13	2.84E+06	PDX tumour	99.29
**Unknown 148**	PDX tumour	14	3.31E+06	PDX tumour	99.37
**Unknown 148**	PDX tumour	13	2.58E+06	PDX tumour	99.25
**Unknown 148**	PDX tumour	14	2.16E+06	PDX tumour	99.37
**Unknown 148**	PDX tumour	13	4.39E+06	PDX tumour	99.2
**Unknown 148**	PDX tumour	14	4.75E+06	PDX tumour	99.3
**Unknown 148**	PDX tumour	14	3.53E+06	PDX tumour	99.34
**Unknown 148**	PDX tumour	13	4.47E+06	PDX tumour	99.38
**Unknown 148**	PDX tumour	14	3.19E+06	PDX tumour	99.33
**Unknown 148**	PDX tumour	14	3.17E+06	PDX tumour	99.32
**Unknown 148**	PDX tumour	12	3.20E+06	PDX tumour	99.3
**Unknown 148**	PDX tumour	13	4.39E+06	PDX tumour	99.31
**Unknown 148**	PDX tumour	13	3.26E+06	PDX tumour	99.27
**Unknown 148**	PDX tumour	14	3.49E+06	PDX tumour	99.33
**Unknown 148**	PDX tumour	12	2.36E+06	PDX tumour	99.35
**Unknown 148**	PDX tumour	14	3.39E+06	PDX tumour	99.35
**Unknown 148**	PDX tumour	13	3.59E+06	PDX tumour	99.38
**Unknown 148**	PDX tumour	14	2.35E+06	PDX tumour	99.32
**Unknown 148**	PDX tumour	12	4.77E+06	PDX tumour	99.31

Table S2. The PIRL-MS-based predictions of *n*=2,079 attempted classifications from *n*=148 independent unknown specimens. This table summarizes the classification results generated with AMX by using the Fig. 1A PCA-LDA model to classify *n*=2,079 data points from *n*=148 independent unknown specimens (*n*=30 lymphoma with *n*=446 spectral data points and *n*=118 true PDX across lung, ovarian, head & neck, colon pancreas, mesothelial and esophageal types with a total of *n*=1,633 spectral data points). Out of the total *n*=2,079 sampling attempts, *n*=11 data points possessed lock-mass error, *n*=23 data points possessed signal duration of less than or equal to 3 seconds, and *n*=115 data points were unclassifiable using the parameters selected for AMX[1] recognition (see the methods section). Therefore, the total number of classifiable data points for calculations of sensitivity and specificity of the method at 93% duty cycle (ratio of unclassifiable and bad data to total, *n*=149 to *n*=2,079), was *n*=1,930. This table also summarizes signal intensity and signal duration which for this dataset (± 0.5 standard deviation) were $(4\pm2)\times10^6$ and 10±1 seconds, respectively (values rounded based on scientific figures). This table also suggests that (98±2)% of the sampling data were spatially concordant and that the average probability in prediction for assessments made using Fig. 1A model was high at (98±2)%, calculated as average ± 0.5 standard deviation. A total of *n*=33 sampling events

possessed less than 95% confidence in probability threshold. However, we did not use probability thresholding to improve the performance of the model, as done previously[2].

Full Group Out Cross Validation - Python Model

Group	PIRL-MS spectra data points	Correctly classified data points	Misclassified data points	Correct Classification Rate
1	275	275	0	100%
2	252	248	4	98.41%
3	269	265	4	98.51%
4	235	232	3	98.72%
5	283	277	6	97.88%
**Total**	**1314**	**1297**	**17**	**98.70%**

	Lymphoma	PDX tumour	**Total**
Lymphoma	258	11	**269**
PDX tumour	6	1039	**1045**
**Total**	**264**	**1050**	**1314**

Table S3. The results of 5-fold cross-validation leave out test of Fig. S4 PCA-LDA model (Python). This table uses the same 'data matrix' used to generate Fig. 1A (and Fig. S4), but instead was subjected to PCA-LDA modeling in Python using SciKit-learn package. The results shown in this table (with the caveats reported in the legend of Fig. S4 regarding 'outlier detection') are comparable to those obtained on AMX (reported in Table 1). Therefore, proprietary access to AMX is not required for future implementation of the method.

Full Group Out Cross Validation - SMOTE Model

Group	PIRL-MS spectra data points	Correctly classified data points	Misclassified data points	Correct Classification Rate
1	275	275	0	100%
2	252	246	6	97.62%
3	269	265	4	98.51%
4	235	230	5	97.87%
5	283	279	4	98.59%
**Total**	**1314**	**1295**	**19**	**98.52%**

	Lymphoma	PDX tumour	**Total**
Lymphoma	259	10	**269**
PDX tumour	9	1036	**1045**
**Total**	**268**	**1046**	**1314**

Table S4. The results of 5-fold cross validation leave out test of Fig. S4 PCA-LDA model using Synthetic Minority Oversampling TEchnique (SMOTE). This table shows the cross-validation accuracies from a 5-fold leave out test utilizing the same dataset used in Fig. 1A/S4 (and cross validation results reported in Tables 1 and S3). As seen here, the SMOTE algorithm, which tries to offset the imbalanced nature of the dataset (269 lymphoma events vs. 1,045 PDX), renders comparable cross-validation accuracy results to Table S3 indicating the robustness of previously described models. The SMOTE algorithm was implemented in Python using the imblearn package.

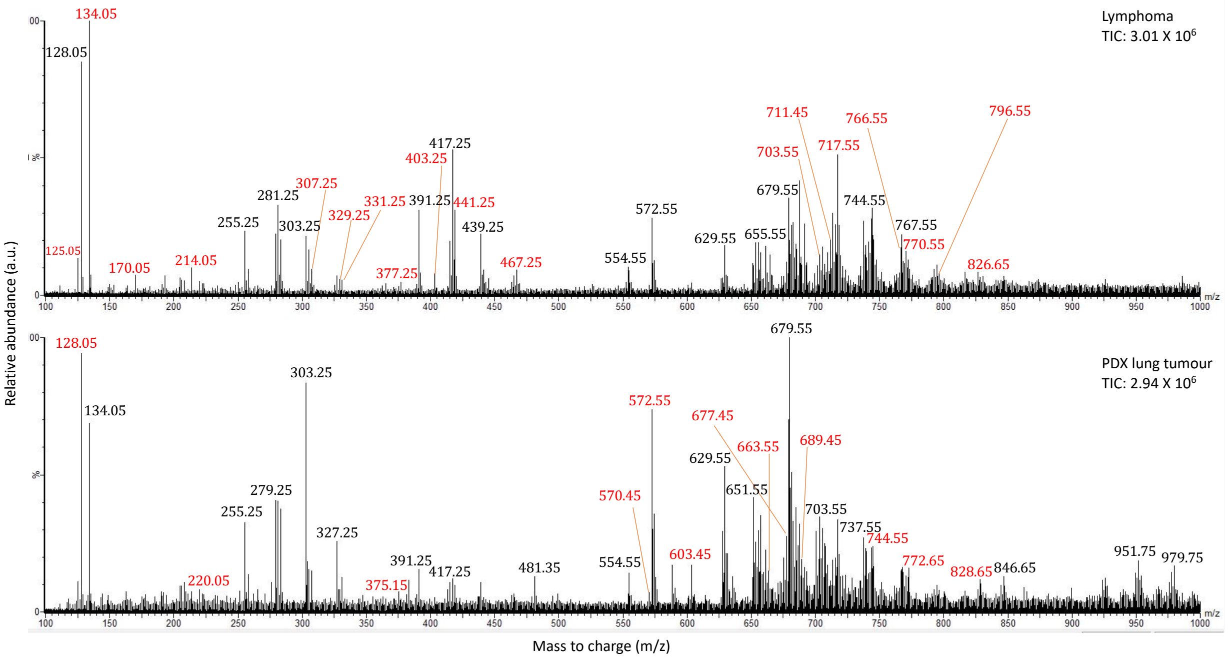

Figure S1. Representative 10-second PIRL-MS spectra of lymphoma PDX and a lung cancer PDX. Here we show the representative PIRL-MS spectra (binned values shown, to 0.1 Da) of a lymphoma PDX and that of a lung cancer representative alongside the total ion count (TIC) values for these spectra. We have highlighted the abundant *m/z* values seen in these spectra (**black** font). Additionally, a fraction (~30) of the most important *m/z* values for the differentiation of lymphoma outgrowths from true solid tumour PDXs (through loading plot analysis of Fig. 1A PCA-LDA model, listed in Table S1) visible in these representative spectra are highlighted in **red** font. The 10-second PIRL-MS spectra across the mass range of 100-1,000 Da (Daltons) possesses distinct molecular profiles for these two tumour types.

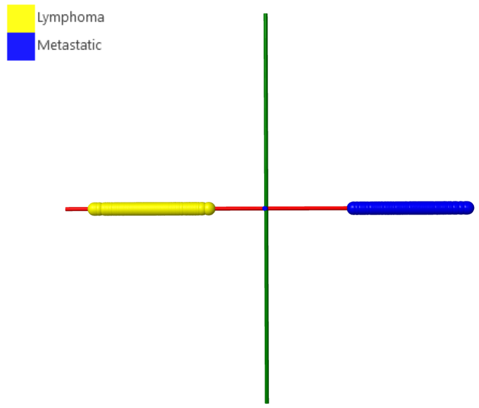

Figure S2. PCA-LDA plot of human primary lymphoma versus human solid tumours. This PCA-LDA plot was created on AMX using *n*=22 human primary lymphoma and *n*=22 human metastatic (to the brain) epithelioid tumours of 16 lung, 5 colon and 1 ovarian origin. The raw data reanalyzed for this plot is presented in a submitted manuscript [3]. The data shows separation (*n*=235, 10-second sampling events from lymphoma and *n*=191 events from epithelioid cancers). Only tumour cores were analyzed here. This PCA-LDA model possessed 100% cross-validation accuracy (from 5-fold leave-out test). Fig. S3 shows the 'loading plot' associated with the PCA-LDA model.

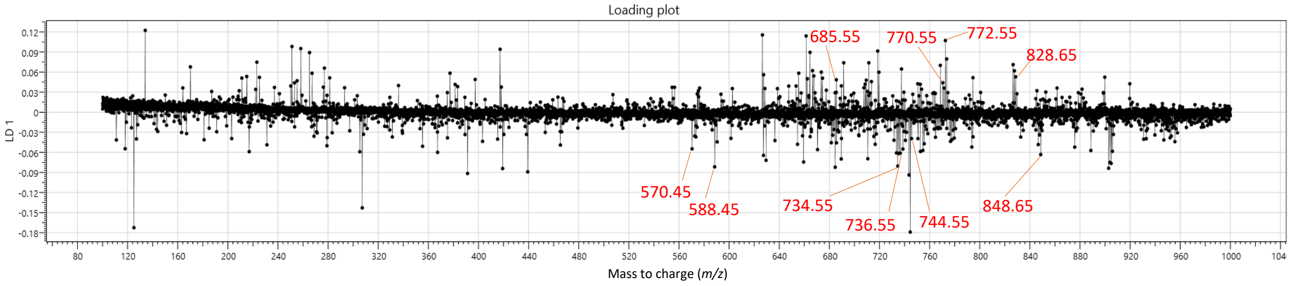

Figure S3. The loading plot of Fig. S2 PCA-LDA, highlighting ten *m/z* values with known identities from our parallel research work[3]. The loading plot shows the rank order with which each m/z value contributes to the statistical separation seen between data groups in Fig. S2 PCA-LDA model. While several high-ranking *m/z* values remain to be identified (e.g., *m/z* values 125.05, 134.05, 307.25, 417.25 and 626.55 and 661.45 among others), the previous work on human tissue[3] establishes the identities of *m/z* 828.65 as PE(42:1); *m/z* 796.55 as PE(40:3); *m/z* 770.55 as PC(36:2); *m/z* 744.55 as PE(36:1); *m/z* 685.55 as SM(d34:2) and *m/z* 570.45 as Cer(d34:2). For this manuscript, the raw mass spectral data described in[3] was subjected to new modeling to generate the Fig. S2 PCA-LDA plot and the Fig. S3 loading plot. A subset of ion identity assignments relevant to the Fig. S2 model is provided in this legend. It must be understood that said work[3] has been cited to address reviewer comments at the revision stage requiring a discussion on the identities of molecular drivers behind mass spectral differences seen between lymphoma and solid tumours, albeit in a PDX model. We strived to address this comment using existing patient tissue data included in this submitted manuscript[3] per reviewer suggestion. Since patient tissues are devoid of mouse stroma (present in PDX models), this approach simplified the analysis and enhanced interpretability of results.

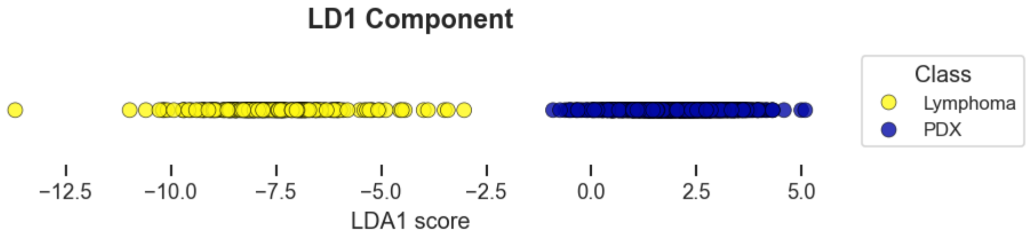

Figure S4. PCA-LDA scores plot of a model based on Fig. 1A data (AMX) created by Python. This figure shows the scores plot of the same dataset that was processed by PCA-LDA in AMX and used the SciKit-learn package as detailed in the experimental section. Here, consistent with AMX results of Fig. 1A, a separate grouping between the lymphoma and tumour classes is seen. Table S3 shows the accuracy of this model calculated from 5-fold cross validation. One caveat is that our current Python platform does not consider 'unclassifiable data' (i.e., does not perform outlier detection). As such, it slightly underperforms compared to AMX (Table 1) results, with cross-validation accuracy of 98.70% compared to 100% on AMX. This slight discrepancy warrants further implementation of 'outlier detection' using Mahalanobis distances as done on AMX. However, given that the mass spectra of lymphoproliferative tumours are drastically different from those of the epithelioid tumours, an outlier detection algorithm is unlikely to be needed for future implementation.

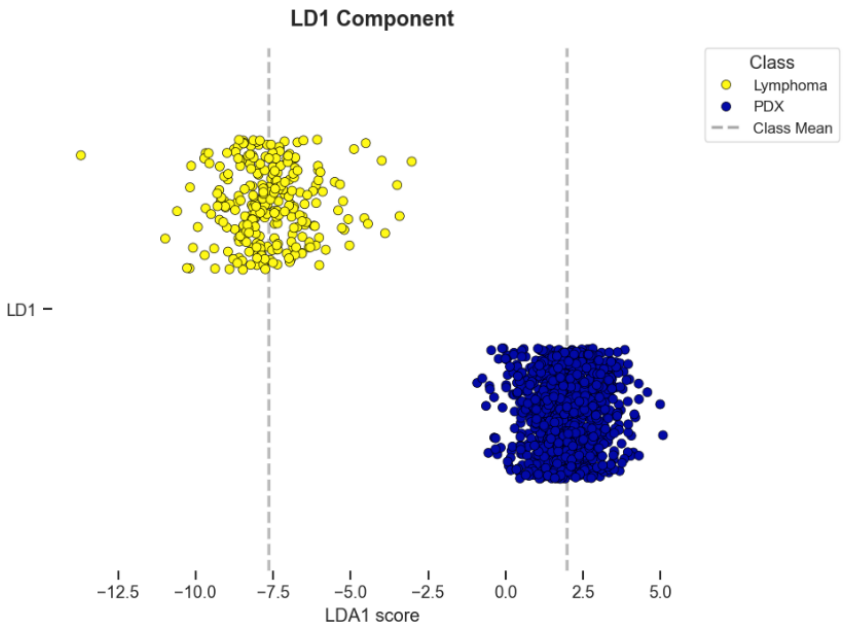

Figure S5. A Jitter plot corresponding to Fig. S4/Fig.1A PCA-LDA scores plot. A jitter plot shows the degree of spectral heterogeneity that is otherwise collapsed in the 1-dimensional projections provided along Linear Discriminant axis 1 (LDA1) in Fig. 1A/S4. This feat is not achievable on AMX. A deeper interpretation of the jitter plot is beyond the scope of this work. However, it is immediately evident that lymphoproliferative tumours exhibit significant heterogeneity. This is not surprising given that in our dataset we have not distinguished between lymphoproliferative lesions of murine and/or human origin. For clarity, the mean LDA1 score for each class is also indicated on the plot.

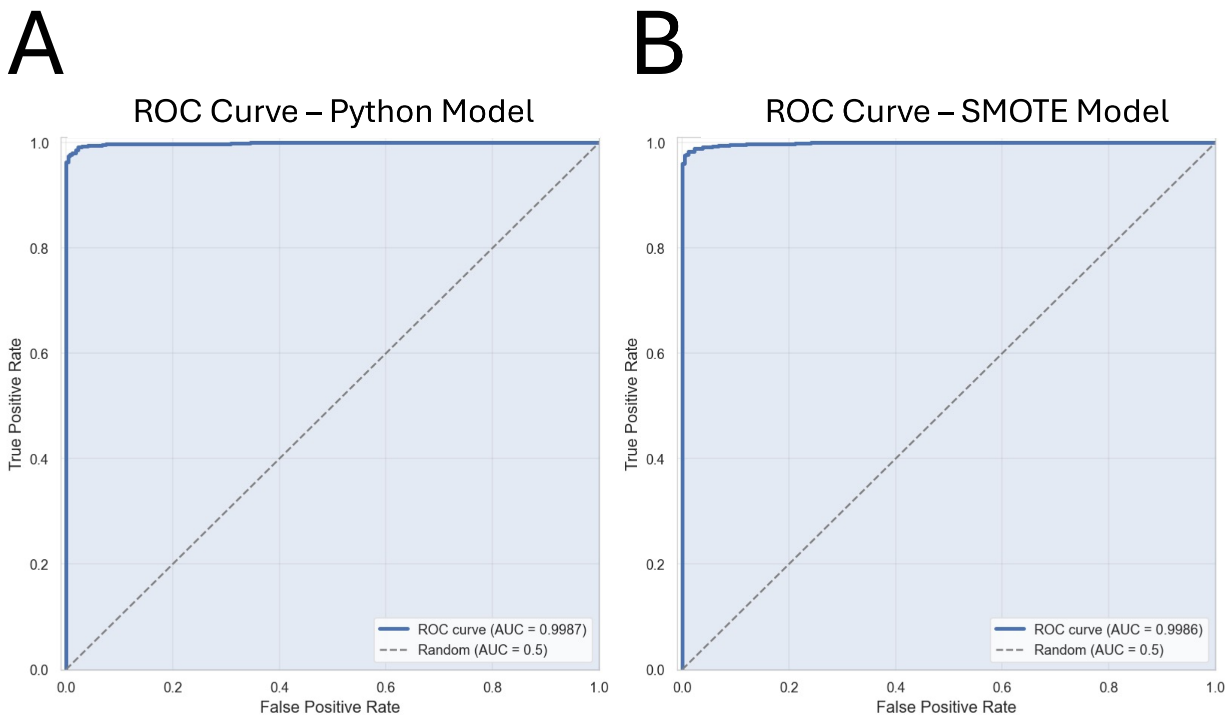

Figure S6. Area under the Receiver Operating Characteristic (ROC) curves [AUC] presented for both PCA-LDA (Python) and PCA-LDA SMOTE (Python) models described in Tables S3 and S4, respectively. Panel A shows the area under the ROC for Table S3 model that uses PCA-LDA, and the previously described imbalanced dataset and panel B shows the same for Table S4 model (PCA-LDA augmented with SMOTE). As shown here, both models suggest a significant difference between lymphoproliferative lesions and solid tumour PDXs of epithelioid origin, consistent with multivariate analysis results presented and used to validate the model throughout this manuscript. It must be emphasized that area under ROC analysis is a threshold independent performance metric and orthogonal to PCA-LDA classifications.

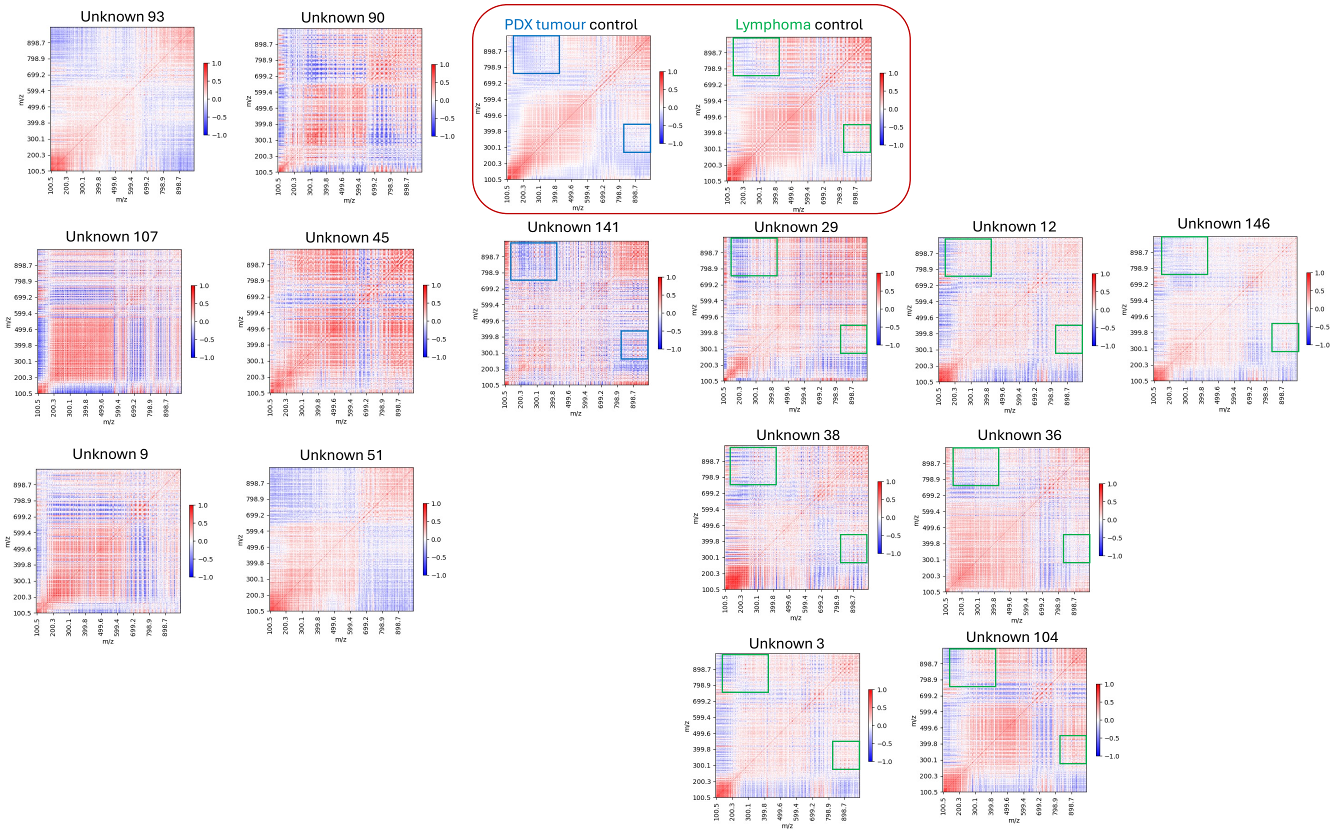

Figure S7. Spectral correlation maps to explain the origin of misclassifications. In this figure, we show the Pearson correlation maps for each of the features using mass spectra binned to 1 Da (900 x 900 feature matrix). For each of the standard (control) correlation maps created from the 100% correctly classified unknowns (27 lymphomas and 107 PDXs), we have highlighted the 'fingerprint' regions unique to each tumour type (indicated with boxes shown). Visual inspection of these maps allows some insights into the presence of mass spectral correlations typical of the misclassified entities. As shown, unknowns 3, 12, 29, 36, 38, 104, and 146 possessed lymphoma-like signatures, and correlation patterns typical of PDX were seen in unknown 141. Here, we failed to detect clear indications supporting misclassification behaviour of unknowns 9, 45, 51, 90, 93, and 107. These, however, possessed correlation maps that were drastically different from either lymphoma control or PDX standard.

References

1 Bodai, Z. *et al.* Effect of Electrode Geometry on the Classification Performance of Rapid Evaporative Ionization Mass Spectrometric (REIMS) Bacterial Identification. *J Am Soc Mass Spectrom* **29**, 26–33 (2018). https://doi.org/10.1007/s13361-017-1818-5

2 Woolman, M. *et al.* Picosecond Infrared Laser Desorption Mass Spectrometry Identifies Medulloblastoma Subgroups on Intrasurgical Timescales. *Cancer Res* **79**, 2426–2434 (2019). https://doi.org/10.1158/0008-5472.CAN-18-3411

3 Fiorante, A. V., D.; Woolman, M.; Munoz, D.; Kiyota, T.; Wu, M.; Ye, L.A.; Talbot, F.O.; T, K.; Das, S.; Kellett, S.; Giuffrida, C.; Mehta, D.; Zadeh, G.; Kridel, R.; Nassiri, F.; Ginsberg, H.; Aman, A.; Zarrine-Afsar, A. Non-subjective diagnosis of lymphoma, glioma and metastatic carcinoma on intrasurgical timescales to optimize resection strategies. **Submitted** (2025).
